# 5-Chloro-8-nitro-1-naphthoyl (NNap): A Selective Protective Group for Amines and Amino Acids

**DOI:** 10.1021/acs.orglett.3c01334

**Published:** 2023-05-26

**Authors:** Asmaa Habib, José J. Garrido-González, Estela Sánchez-Santos, Irene Boya del Teso, Francisca Sanz, Victoria Alcázar, Ángel L. Fuentes de Arriba, Joaquín R. Morán

**Affiliations:** † Organic Chemistry Department, University of Salamanca, Plaza de los Caídos s/n, Salamanca 37008, Spain; ‡ X-Ray Diffraction Service, University of Salamanca, Plaza de los Caídos s/n, Salamanca 37008, Spain; ∥ Environmental and Industrial Chemical Engineering Department, Polytechnic University of Madrid, C/José Gutiérrez Abascal, 2, Madrid 28006, Spain

## Abstract

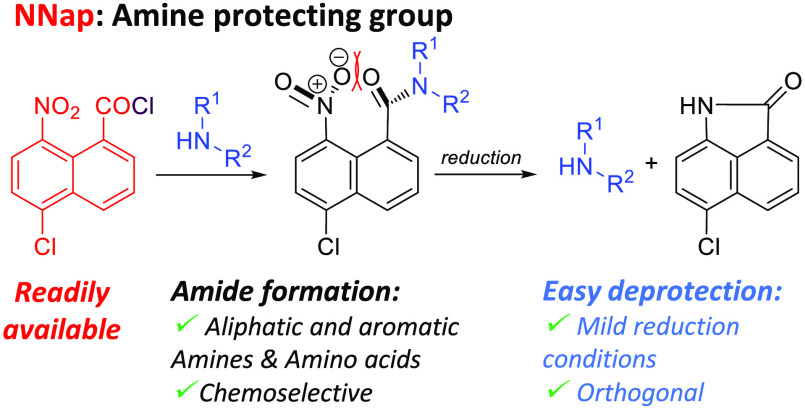

The synthesis of 5-chloro-8-nitro-1-naphthoyl chloride and its use as a protective group for amines is described. Protection is carried out with an auxiliary amine or under mild Schotten-Baumann conditions in high yield (>86%), while deprotection can be achieved easily under gentle reducing conditions due to the large steric tension between C-1 and C-8 naphthalene substituents. The reaction has been successfully tested in dipeptide synthesis and amino alcohols protection, and it has proved selective for the ε-amine group of lysine.

The protection of amino groups is a highly studied synthetic strategy.^1^ Nevertheless the complexity of organic synthesis usually requires several different protecting groups with orthogonal properties, and therefore, new amine protective groups are welcome.

Amides are very stable functional groups, and thus, cleavage of the amide bonds usually requires strong acid or base-catalyzed hydrolysis to release the amines. To overcome this problem a popular solution is to use carbamates as the amine protecting groups.^2^ Release of CO_2_ is the main driving force of the deprotection reaction, which favors liberation of the amine. However, carbamates also present some drawbacks, such as their high toxicity or the presence of two different conformations, which, in many cases, might complicate the NMR spectra. Besides, chlorocarbonates are also unstable compounds which can decompose with generation of CO_2_, creating high pressure inside the vessel that can lead to explosions.

In this paper we explore an alternative solution which implies the formation of a sterically stressed amide that can be later deprotected under unusual mild reductive conditions based on a strong release of steric strain. The protective group is the 5-chloro-8-nitro-1-naphthoyl (NNap) that can be introduced as an acid chloride under standard or Schotten-Baumann conditions^3^ affording the corresponding amides in high yield (Table 1).

**Table 1:** Acylation of Amines with 5-Chloro-8-nitro-1-naphthoyl chloride 4 (NNapCl)^a^

entry	amine	amide (%)^b^
1	*n*-octylamine (**5**)	**12** (86%)
2	*n*-decylamine (**6**)	**13** (95%)
3	benzylamine (**7**)	**14** (87%)
4	*tert*-octylamine (**8**)	**15** (90%)
5	di-*n*-butylamine (**9**)	**16** (87%)
6	4-*tert*-butylaniline (**10**)	**17** (91%)
7	(1*S*,2*S*)-(+)-*trans*-1-amino-2-indanol (**11**)	**18** (86%)

a Reaction conditions: amine (1 equiv) in CH_2_Cl_2_ was added dropwise to a solution of the acid chloride NNapCl, **4** (1.0 equiv) in CH_2_Cl_2_ at 0 °C. After stirring 5 min, an aqueous Na_2_CO_3_ (5.0 equiv) solution was added and the mixture was allowed to warm to rt, stirring for an additional 25 min.

b Isolated yields.

The large steric strain in 1,8-disubstituted naphthalenes is well-known,^4^ and this steric strain is usually relieved by bond bending with the substituents twisted out of the plane of the aromatic rings. Thus, 1,8-bis(dimethylamino)naphthalene (p*K*_a_ = 12.1)^5^ is a stronger base than dimethylaniline (p*K*_a_ = 5.15), because protonation reduces the electrostatic and steric strain (Figure 1A). The X-ray crystal structure of 8-(dimethylamino)-1-naphthyl methyl ketone^6^ illustrates the Burgi-Dünitz trajectory in which the combination of the steric compression and the bonding interaction between the lone pair of the amine and the carbonyl group deforms the molecule (Figure 1B).^7^ Another example is the high reactivity of 8-dimethylamino-1-naphthaldehyde, in which treatment with benzoyl chloride^8^ leads to benzoylation of the aldehyde oxygen and addition of the *peri*-dimethylamino group to the former carbonyl group, setting a new N–C bond (Figure 1C).

**Figure 1:**
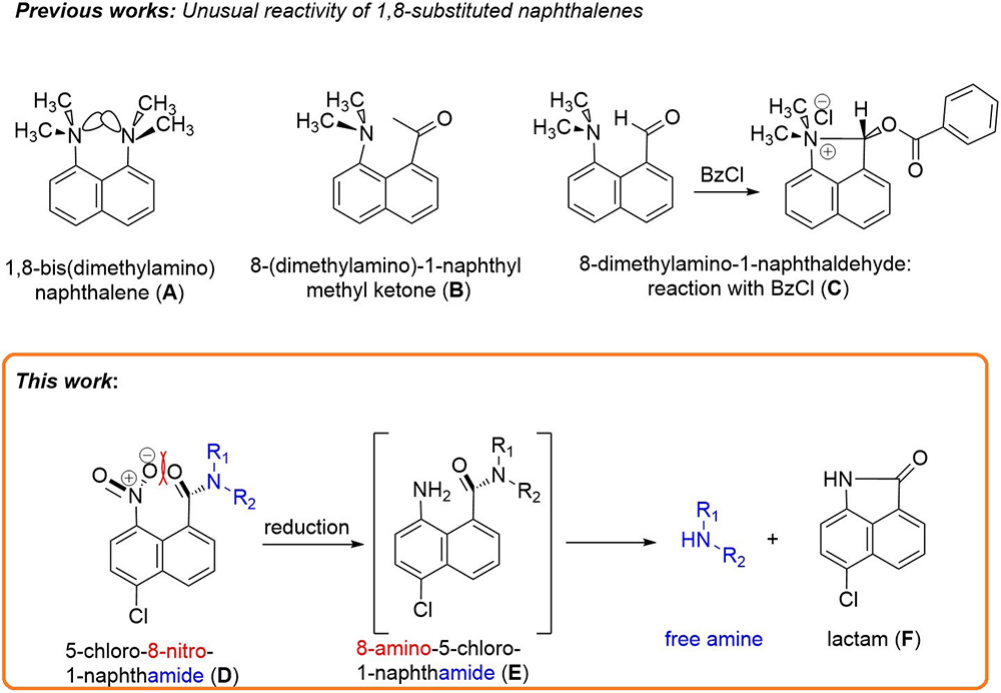
Unusual reactivity of some 1,8-substituted naphthalenes and sterically hindered amides synthesized in this work.

In our case and in a similar way, reduction of the 8-nitro group to amine (**E**) leads to a decrease in the steric strain, due to the formation of a N–C bond between the *peri* substituents, the amine nitrogen at C-8, and the amide carbonyl group at C-1. Thus, formation of the five-membered lactam (**F**) is accompanied by release of the protected amine (Figure 1).

The preparation of the protecting group started from the commercially available 1-naphthoic acid **1** (Scheme 1). Direct nitration of this compound takes place in the C-5 position; therefore, previous bromination was carried out to block this position. Nitration in acetic anhydride using *p*-TsOH as catalyst yielded a clean reaction in the expected C-8 position. Treatment of the acid **3** with thionyl chloride afforded the acid chloride **4** with concomitant halogen exchange. The replacement of bromine by chlorine was experimentally confirmed: hydrolysis of the acid chloride **4** with water produced a carboxylic acid (**3a**), whose spectral properties (NMR and MS) showed that chlorine had replaced the C-5 bromine.

**Scheme 1:**
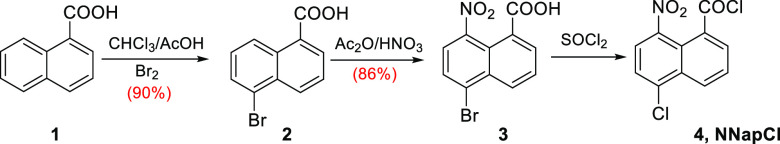
Synthesis of the Protective Group (NNapCl)

Both the carboxylic acid **3** and its potassium salt are highly crystalline compounds. Slow evaporation of an aqueous equimolar solution of potassium hydroxide and the acid **3** allowed us to obtain high-quality crystals suitable for X-ray diffraction analysis. The structure shows a strong steric interaction between substituents at C-1 and C-8 positions that are placed outside the plane of the naphthalene ring with torsion angles of 40°–48° (Figure 2).

**Figure 2:**
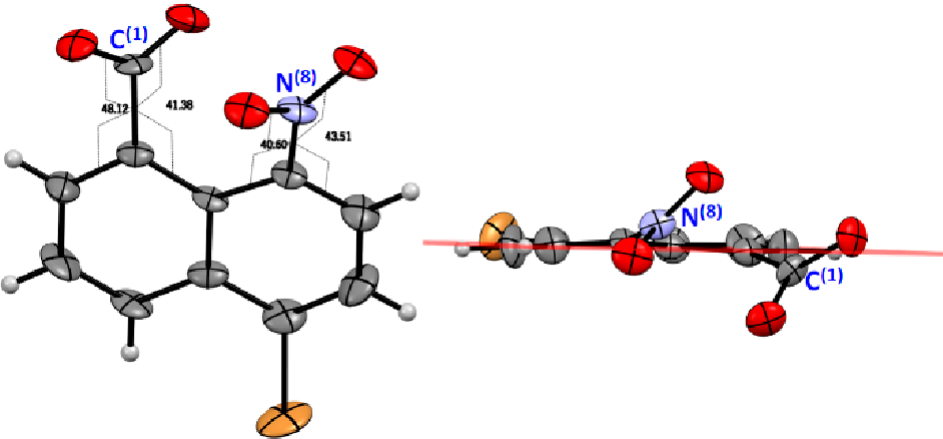
ORTEP diagram of the potassium salt of the carboxylic acid **3**. K^+^ counterion and a crystallization water molecule have been omitted for clarity.

Amine protection was carried out under Schotten-Baumann conditions in a two-phase reaction using methylene chloride and aqueous sodium carbonate solution; easy workup using liquid–liquid extraction was enough to obtain the amides (**D**) with high purity, and usually no chromatography was needed. Thus, reaction of the amine with 1.0 equiv of the acyl chloride **4** and in the presence of Na_2_CO_3_ (5.0 equiv) afforded the corresponding amide in good to excellent yields (Table 1, entries 1–6). Primary and secondary amines were tested: *n*-octylamine (**5**), *n*-decylamine (**6**), and benzylamine (**7**) afforded, in 30 min, a quantitative NMR yield of the desired amides (86–95% isolated yields). The more hindered primary amine *tert-*octylamine (**8**) and the secondary amine di-*n*-butylamine (**9**) also gave the corresponding amides in high yields (87–90% isolated yields). To further explore the substrate scope, an aromatic amine, 4-*tert*-butylaniline (**10**), was tested; as shown in Table 1 (entry 6), the aniline **10** was easily transformed to the target amide in 91% isolated yield under the same reaction conditions.

Pleasingly, single crystals of the amide **17** were obtained by slow evaporation of a methanol solution. The X-ray structure showed the same features as those of the potassium salt of acid **3**; the nitro and amide groups are twisted from the plane of the naphthalene ring, lying on opposite sides of the plane. Additionally, the replacement of bromine by chlorine at the C-5 position was corroborated (Figure 3).

**Figure 3:**
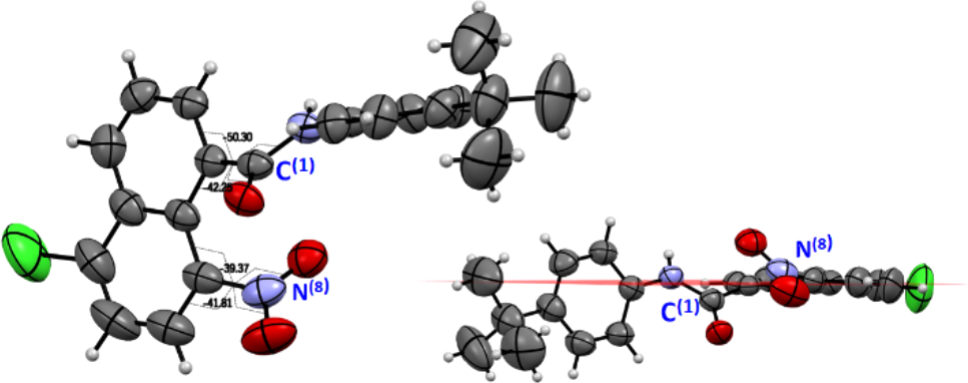
ORTEP diagram of amide **17**. The crystallization methanol molecule has been omitted for clarity.

Removal of the protecting group was studied using a variety of reducing conditions with amide **16** as model substrate (Table 2). The reduction of the nitro group is a widely used transformation, and many methods are available.^9^ In our case, due to the unique reactivity of the *peri*-disubstituted naphthalenes, the reduction of the nitro group occurs under mild conditions producing cleanly the desired amine in high yields, along with the expected lactam **F**. Although our attempts to grow single crystals of the lactam **F** were not successful, the crystal structure of the closely related 1,8-naphtholactam is known.^10^ The steric hindrance between *peri* positions in compounds **3** and **16**, twisted on either side of the naphthalene plane, is no longer present in the 1,8-naphtholactam, which possesses a nearly absolute planar configuration.

**Table 2:** Removal of the Protecting Group

entry	conditions	yield of 9 (%)^a^
1	Zn/AcOH; 60 °C, 10 min	95%
2	SnCl_2_/MeOH; 60 °C, 10 min	80%
3	H_2_/Pd (C)/AcOEt; 20 °C, 12 h	90%

a Isolated yields.

The best results in the reduction reaction were obtained using Zn/AcOH (Table 2, entry 1): a solution of the amide **16** in acetic acid was added to a preheated suspension of Zn in acetic acid affording di-*n*-butylamine **9** in 95% yield and lactam **F**.

The progress of the reduction could be monitored in an NMR tube (Figure 4). Starting from the 5-chloro-8-nitro-1-naphthamide **16** (spectrum at the bottom), nitro group reduction to amine (**E16**) took place immediately following mixing (second spectrum from the bottom); however, in these dilute conditions (CD_3_OD as solvent), intramolecular cyclization to lactam **F** required heating in a water bath at 60 °C for several hours. Thus, ^1^H NMR signals corresponding to both the amine (**E16**) and lactam (**F**) could be easily identified in the different spectra until total conversion was achieved (lactam **F** spectrum at the top).

**Figure 4:**
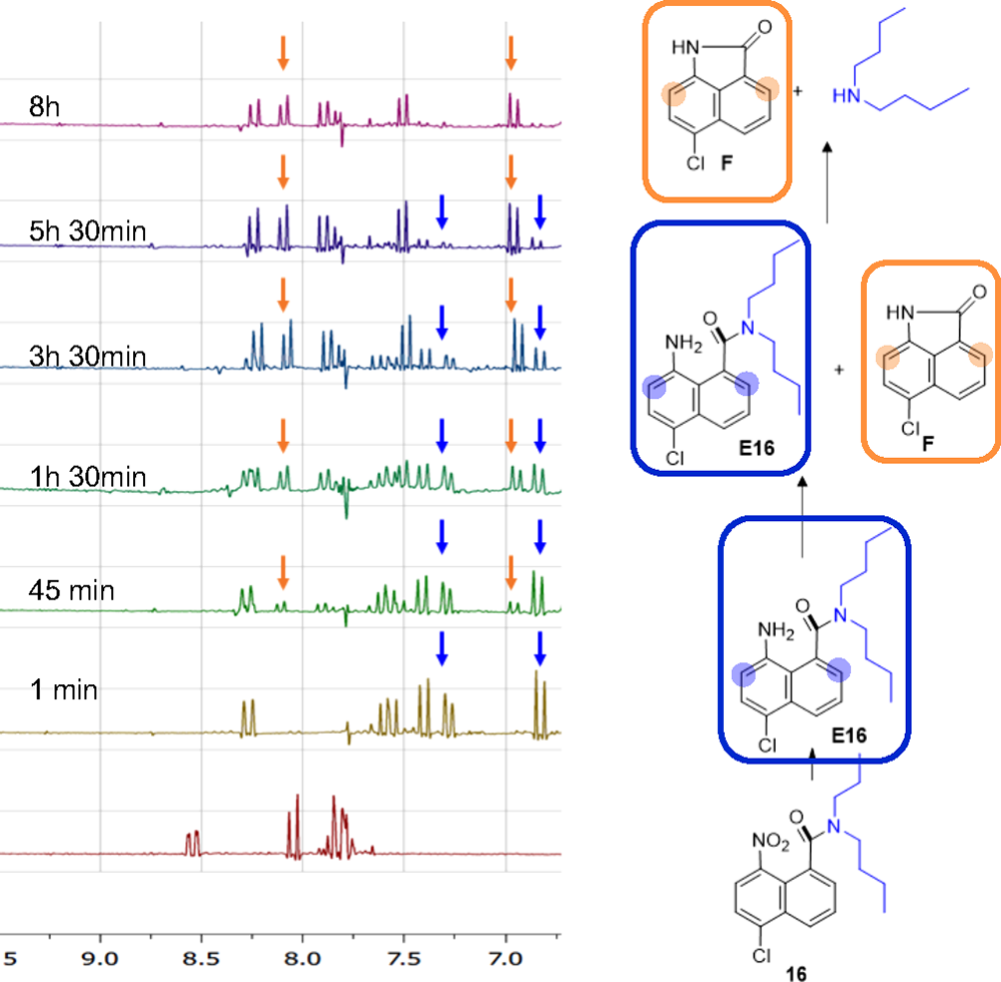
^1^H NMR spectra (aromatic region) of the progress of the reduction of amide **16** with Zn/AcOH: from the intermediate amine **E16** to the lactam **F**. Reaction conditions: amide **16** (10.0 mg), Zn (80.0 mg), and acetic acid (20 mg) were dissolved in 1.0 mL of CD_3_OD at 20 °C.

Attempts to isolate the intermediate aromatic amine (**E16**) were unsuccessful, but when the reaction mixture was treated with acetic anhydride, the amine (**E16**) was trapped as the acetamide (**Ac16**). Interestingly, one of the butyl chains of the acetamide **Ac16** is highly shielded: methyl triplet (4-Bu) at 0.53 ppm, probably because it is over the naphthalene ring (Figure 5). Rotating-frame nuclear Overhauser effect spectroscopy (ROESY) also shows a correlation of the methylene protons (1-Bu and 2-Bu) with the aromatic C-2 proton (7.28 ppm) of the naphthalene ring (Figure 5, inset). The absorption of the C-2 proton at 7.28 ppm is also very unusual, since it is *ortho* to a carboxylic group: the twist of the amide carbonyl group from the plane of the aromatic ring explains this unusual shift. On the other hand, one of the methylene protons of the other butyl chain (1′-Bu) is strongly deshielded (4.10 ppm) due to the proximity with the amide carbonyl group. These effects show a rigid conformation in this part of the molecule, probably due to an angular H-bond between the acetamide and the amide carbonyl group (Figure 5). Two-dimensional (2D) NMR spectra and molecular modeling studies allowed us to propose a possible geometry for the molecule with one butyl chain pointing toward the shielding zone of the naphthalene ring. A similar effect was observed for the nonacetylated compound, the amine **E16**.

**Figure 5:**
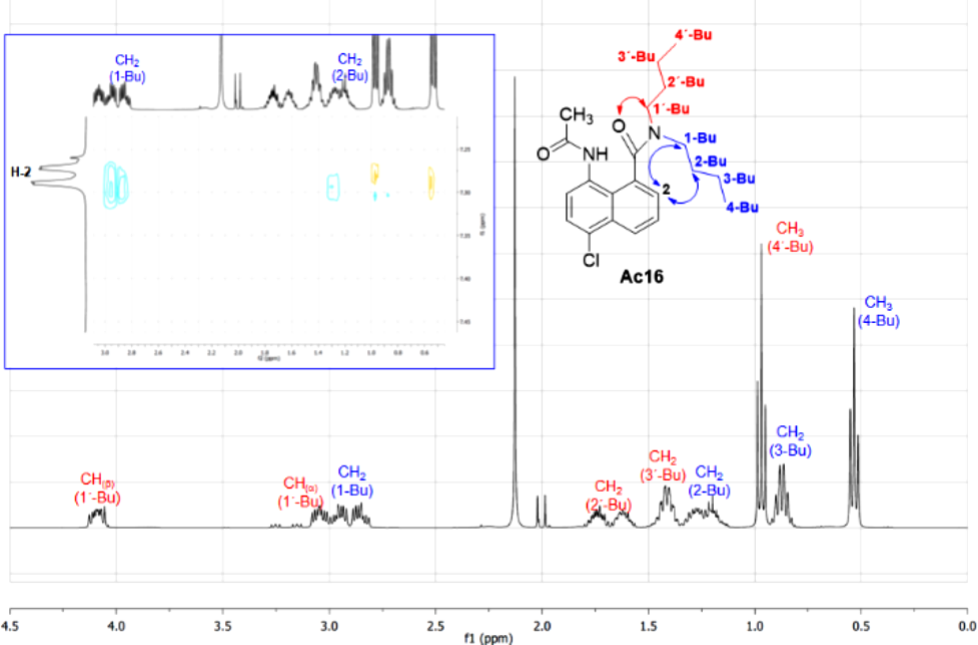
^1^H NMR spectra (aliphatic region) of acetamide **Ac16** in CDCl_3_ at 20 °C and ROESY spectrum (inset) showing correlations between naphthalene C-2 proton and methylenes (1-Bu and 2-Bu).

Having optimized the reaction conditions, we explored the scope of the deprotection step with amides **12** (*n*-octylamide), **15** (*tert*-octylamide), and **17** (4-*tert*-butylanilide); in all cases, deprotection proceeded in excellent yields (>90%).

Finally, deprotection was tested using a reducing agent which mimics the physiological NADPH. Hantzsch ester reduced successfully the nitro group of amide **12** in the presence of eosin Y under 40 W white light irradiation. While the amine **5** (70% yield) is nicely liberated from the protecting group, surprisingly the lactam **F** was not obtained. Analysis of the reaction products showed 8-amino-5-chloro-1-naphthoic acid as the main product. Since this is not the expected compound the structure was confirmed through comparison with a synthesized compound obtained from the reduction of the nitroderivative **3a** under basic conditions.

As the protection of the amino functionality plays an essential role in peptide synthesis, we explored the utility of the NNap protective group in the synthesis of l-leucyl-l-leucine. Hence, l-leucine was first reacted with NNapCl **4** affording amide **19** in high yield (Scheme 2). The synthesis of the dipeptide **20** was completed by *i*-Pr_2_NEt/DCC mediated coupling between H-Leu-O*t*Bu·HCl and **19** (Scheme 2). No racemization was observed.

**Scheme 2:**
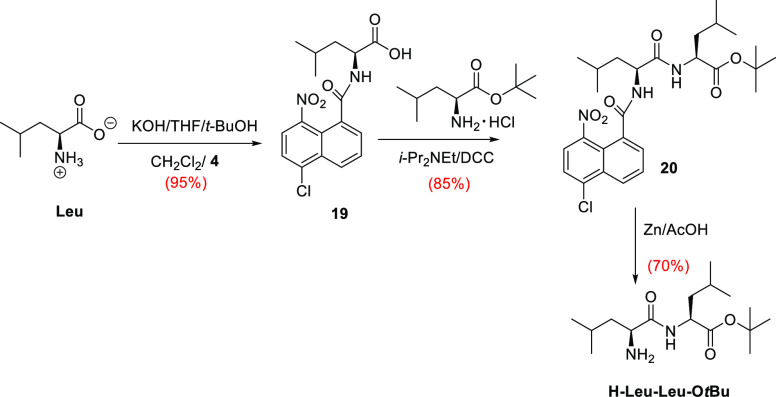
Application to H-Leu-Leu-O*t*Bu Synthesis

One of the main challenges in peptide synthesis is related to selective protection of the ε- and α-amino groups of l-lysine.^11^ Common protective groups such as benzyloxycarbonyl (Cbz) or fluorenylmethoxycarbonyl (Fmoc) are not selective, and protection of the ε-amino lysine usually requires blocking the α-amino group using copper salts.^12^ Likewise, benzyloxycarbonylation of the α-amino group involves temporary blocking of the ε-amino by condensation with benzaldehyde.^13^ These procedures are time-consuming and imply the use of additional reagents, so the development of new methods for orthogonal selective protection of the amino groups of lysine is highly desirable. A supramolecular approach using cyclodextrins (β-CDs) has shown strong regioselectivity and good yields in the Cbz protection of the ε- and α-amino groups.^14^ The reaction has been performed on 1 mmol scale (1 mmol Lys; 0.1 mmol β-CD), and its selectivity appears to be limited to the Cbz group and the β-CD.

Prompted by our previous results, we explored the reaction with lysine; and after finding the optimal conditions, to our delight, treatment of the l-lysine methyl ester dihydrochloride (H-Lys-OMe 2HCl) with NNapCl **4** resulted in selective and high yield protection of the ε-amino group (Scheme 3).

**Scheme 3:**
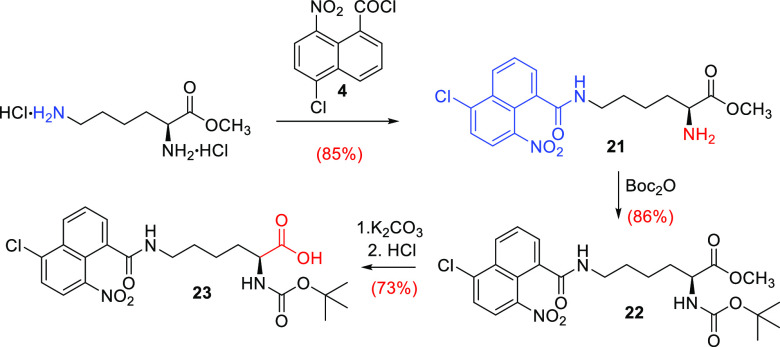
Application to Orthogonal Protection of l-Lysine

The observed regioselectivity in favor of the ε-amino group yielding compound **21** might be justified by the highly sterically hindered naphthoyl chloride **4**. The amino ester **21** was amenable to further synthetic elaborations, such as protection of the α-amino group (**22**) or hydrolysis to carboxylic acid (**23**). Applying the strategy of orthogonal functional group protection, the free α-amino group was acylated with Boc_2_O to afford **22** in good yield. Finally, the deprotected acid **23** was obtained by hydrolysis of the methyl ester under extremely mild conditions: aqueous potassium carbonate in methanol at room temperature. No sign of racemization was observed.

A further example showing the potential of this protecting group is demonstrated using indanolamines. First, indanolamine **11** was chemoselectively protected by treatment with NNapCl **4** (Table 1, entry 7), and next, the less nucleophilic hydroxyl group was esterified with benzoyl chloride. Finally, compound **24** was reacted with Zn/AcOH, and the amine functionality was deprotected without affecting the more sensitive ester group. No transposition to the amino group, neither racemization, was observed in compound **25** (Scheme 4).

**Scheme 4:**
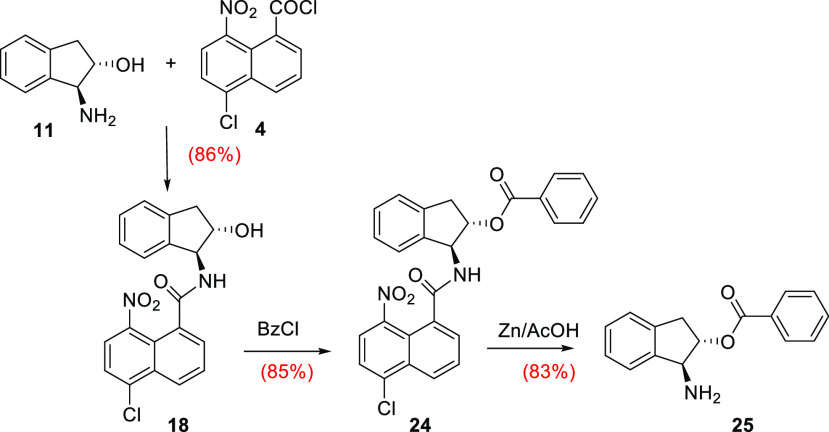
Application to Indanolamines

In conclusion, we have developed a readily available and efficient protective group for amines, the 5-chloro-8-nitro-1-naphthoyl (NNap). The steric hindrance between *peri* positions in the naphthalene ring allows deprotection under mild reduction conditions and selectivity in the case of lysine. This protective group is also suitable for aminoalcohols. The yields of the protection and deprotection steps are excellent, and both reactions proceed under mild reaction conditions. These promising results suggest that this new protective group has great potential to be further developed and included as an alternative for amine protection.

## Data Availability

The data underlying this study are available in the published article and its Supporting Information.

## Supporting Information Available

Complete experimental procedures and characterization data for compounds, including ^1^H, ^13^C, 2D NMR, X-ray crystal analysis, and modeling studies (PDF)
