# Supplementary material for: 5-Chloro-8-nitro-1-naphthoyl (NNap): A Selective Protective Group for Amines and Amino Acids
Source: Org Lett. 2023 May 26;25(22):4103–7. doi: 10.1021/acs.orglett.3c01334 (PMC10262267; doi:10.1021/acs.orglett.3c01334)
Supplement: Supplementary file 1 — ol3c01334_si_001.pdf [file ol3c01334_si_001.pdf]

Supporting Information

5-chloro-8-nitro-1-naphthoyl (NNap): A selective protective group for amines and amino acids

Asmaa Habib, José J. Garrido-González, Estela Sánchez-Santos, Irene Boya del Teso, Francisca Sanz, Victoria Alcázar, Ángel L. Fuentes de Arriba, and Joaquín R. Morán

Table of Contents

1. General information 4

2. Synthesis and Characterization 6

2.1. Synthesis of the protective group 6

- 5-bromo-1-naphthoic acid (**2**) 6
- 5-bromo-8-nitro-1-naphthoic acid (**3**) 6
- 5-chloro-8-nitro-1-naphthoyl chloride NNapCl (**4**) 6
- 5-chloro-8-nitro-1-naphthoic acid (**3a**) 7

2.2. General procedure (A) for amine (**5-11**) protection using NNapCl 7

- 5-chloro-8-nitro-*N*-octyl-1-naphthamide (**12**) 7
- 5-chloro-*N*-decyl-8-nitro-1-naphthamide (**13**) 8
- *N*-benzyl-5-chloro-8-nitro-1-naphthamide (**14**) 8
- 5-chloro-8-nitro-*N*-(2,4,4-trimethylpentan-2-yl)-1-naphthamide (**15**) 8
- *N,N*-dibutyl-5-chloro-8-nitro-1-naphthamide (**16**) 9
- *N*-(4-(*tert*-butyl)phenyl)-5-chloro-8-nitro-1-naphthamide (**17**) 9
- 5-chloro-*N*-((1*S*,2*S*)-2-hydroxy-2,3-dihydro-1*H*-inden-1-yl)-8-nitro-1-naphthamide (**18**) 9

2.3. Procedure for deprotection 10

- Zn/AcOH (B) 10
- $SnCl_2$/MeOH (C) 10
- H_2/Pd (C)/ EtOAc (D) 10
- 6-chlorobenzo[*cd*]indol-2(1*H*)-one (**F**) 11

2.4. Capture of the intermediate amine 12

- 8-acetamido-*N,N*-dibutyl-5-chloro-1-naphthamide (**Ac16**) 12

2.5. Synthesis de H-Leu-Leu-O*t*Bu 13

- (5-chloro-8-nitro-1-naphthoyl)-L-leucine (**19**) 13
- *tert*-butyl (5-chloro-8-nitro-1-naphthoyl)-L-leucyl-L-leucinate (**20**) 14
- *tert*-butyl L-leucyl-L-leucinate (**H-Leu-Leu-O*t*Bu**) 14

2.6. Orthogonal protection of lysine 15

- Methyl N^6-(5-chloro-8-nitro-1-naphthoyl)-L-lysinate (**21**) 15
- Methyl N^2-(tert-butoxycarbonyl)-N^6-(5-chloro-8-nitro-1-naphthoyl)-L-lysinate (**22**) 15
- N^2-(*tert*-butoxycarbonyl)-N^6-(5-chloro-8-nitro-1-naphthoyl)-L-lysine (**23**) 16

2.7. Reaction with 1-amino-2-indanol (**11**) 17

- (1*S*,2*S*)-1-(5-chloro-8-nitro-1-naphthamido)-2,3-dihydro-1*H*-inden-2-yl benzoate (**24**) 17
- (1*S*,2*S*)-1-amino-2,3-dihydro-1*H*-inden-2-yl benzoate (**25**) 18

2.8. NMR spectra 19

3. Crystallographic data 45
3.1. *Potassium carboxylate* (K•**3**) 45
3.2. Amide **17** 49
4. Modelling studies 51
5. References 53

1. General information

Reagents

Reagents were purchased and used without further purification unless otherwise noted. The solvents used were purified and dried following the standard methods. [1]

Purification of reaction crudes

Reactions were monitored by analytical thin layer chromatography using pre-coated aluminium-backed plates (0.2 mm silica gel 60 F254, Merck®) and visualized by UV light. Purification of compounds was performed using silica gel column chromatography (Chromagel 60A SdS. C.C. 70-200 μm) with solvent mixtures of increasing polarity as eluents and crystallization using different solvent mixtures.

Melting Points (m.p.)

Melting points were measured in a Leica Galen III microscope and are reported in ºC.

IR spectroscopy

IR spectra were recorded using a Nicolet IR100 with Nujol as a suspension.

Optical activity

The optical activity was measured with a Perkin-Elmer 341 spectropolarimeter in cells of 10 cm length.

NMR spectroscopy

^{1}H and ^{13}C NMR spectra were recorded at room temperature using Bruker models WP-200-SY and Bruker Avance NEO 400 MHz with a Prodigy CPPBBO BB-H&F z-gradient cryo-probe (400 MHz to ^{1}H and 100 MHz to ^{13}C) spectrometers. Chemical shifts were reported in ppm with the solvent signal (^{1}H/^{13}C: deuterated chloroform $CDCl_3$ 7.26/77.2 ppm, dimethylsulfoxide DMSO-d_6 2.50/39.5 ppm, methanol- d_4 3.30/49.0 ppm or water D_2O-d_2 4.79 ppm) and tetramethylsilane (TMS 0.00 ppm) as an internal standard using reported shifts. [2] Coupling constants (*J*) were reported in Hertz (Hz). The following abbreviations were used to explain the multiplicities: s, singlet; d, doublet; dd, doublet of doublets; t, triplet; q, quartet; quin, quintet; sext, sextet; m, multiplet; br, broad.
Structural assignments were made with additional information from gCOSY, gHSQC, gHMBC and gROESY experiments.

Mass spectrometry

Mass spectra were recorded on a quadrupole-TOF Applied Biosystems QSTAR XL and Waters ZQ 4000 spectrometers using electrospray ionization (ESI) or electronic impact (EI).

X-ray diffraction studies

- *Procedure for the single crystal preparation*

Potassium carboxylate **(K•3)**. Single crystals suitable for X-ray diffraction measurements were obtained by slowly evaporating aqueous solutions of stoichiometric mixtures of the carboxylic acid **3** (50 mg) and the equimolar amount of potassium hydroxide in 3 mL of water in a clean and dry 10 mL glass vial. The mouth of the glass vial was covered with a cap having a small hole and kept it for slow evaporation at room temperature. At the end of the process, single crystals were obtained after 5-6 days.
Amide **17**. Single crystals suitable for X-ray diffraction measurements were obtained by slowly evaporating solutions of the amide **17** (50 mg) in 2 mL of methanol in a clean and dry 10 mL glass vial. The mouth of the glass vial was covered with a cap having a small hole and kept it for slow evaporation at room temperature. At the end of the process, single crystals were obtained after 5-6 days.

- *X-Ray Crystallography*

Suitable single crystals of **K•3** and **17** were mounted on glass fibre for data collection on a Bruker Kappa APEX II diffractometer. Data were collected at 298(2) K using Cu K_{α} radiation (λ = 1.54178 Å) and ω scan technique and were corrected for Lorentz and polarization effects. The detector was placed at approximately 37.5 mm from the crystal.
A series of narrow frames of data were collected with a scan width of 0.5º in ω and an exposure time of 10 s per frame. The data were integrated with SAINT [3] to a resolution of 0.78 Å using a narrow-frame algorithm. Data were corrected for absorption effects using the multi-scan method using SADABS. [4]
Subsequent structure solution and refinement were carried out with SHELXT and SHELXL, respectively. [5, 6] The structures were solved by direct methods combined with difference Fourier synthesis and refined by full-matrix least-squares procedures, with anisotropic thermal parameters in the last cycles of refinement for all non-hydrogen atoms. The refinement was based on F^2 for all reflections, weighted R factors (*wR*) and goodness-of-fit (GoF) values are based on F^2, while conventional R factors (R) are based on F. The $Fo^2 > 2\sigma(Fo^2)$ criterion was used only for calculating R factors and it is not relevant to the choice of reflections for the refinement. The R factors based on F^2 are about twice as large as those based on F. Scattering factors were taken from the International Tables for Crystallography. [7] Hydrogen atom positions were calculated by geometrical methods and refined as a riding model. The potassium salt of the carboxylic acid (**3**) crystallizes with one molecule of water; it should be noticed that hydrogen atoms of water molecules are not shown in the structural formula of the complex, as they were not located by X-ray diffraction. The amide **17** crystallizes with one molecule of methanol.
Mercury 4.2.0 program was used for analysis and molecular and crystal structure drawings preparation. [8]
The CCDC numbers assigned for the crystal structures are: 2252216 (**K•3**, potassium salt of carboxylic acid **3**) and 2252212. (amide **17**).

Modelling studies
Theorical studies were carried out using GAMESS interface for Chem3D 19.1 software [9] using the RHF/3-21G.

2. Synthesis and Characterization

2.1. Synthesis of the protective group

- **5-bromo-1-naphthoic acid (2)**

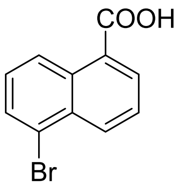

2

This compound was prepared according to a modified published literature. [10]. 1-naphthoic acid (20.0 g, 116.2 mmol) was placed in a 250 mL round bottom flask equipped with a reflux condenser, and $CHCl_3$ (80.0 mL) and acetic acid (10.0 mL) were added. The suspension was refluxed until a homogeneous solution was obtained. Bromine (12.0 mL, 234 mmol) was then added dropwise and after 3 minutes the brominated compound **2** started to precipitate. The thick solution was further refluxed for 2 hours. Cooling down and filtering the precipitate yielded the brominated compound (**2**) (26.0 g, 90% yield) as a white powder. The spectroscopic properties of compound **2** were in agreement with the published data [10]. ^{1}H NMR (DMSO-*d*$_6$, 400 MHz): 8.87 (d, 1H, J = 8.8 Hz), 8.42 (d, 1H, J = 8.5 Hz), 8.22 (dd, 1H, J = 7.2 and 0.8 Hz,), 7.99 (dd, 1H, J = 7.3 and 0.8 Hz), 7.77 (dd, 1H, J = 8.5 and 7.2 Hz), 7.57 (dd, 1H, J = 8.8 and 7.3 Hz).^{13}C NMR (DMSO-*d*$_6$, 100 MHz): 168.3 (C), 131.9 (C), 131.4 (C), 130.9 (CH), 130.6 (CH), 130.4 (CH), 128.9 (C), 128.1 (CH), 126.8 (CH), 125.8 (CH), 122.4 (C). HRMS (ESI) m/z: [M - H]$^-$ Calcd for $C_{11}H_6O_2{}^{79}Br$ 248.9557; Found 248.9559.

- **5-bromo-8-nitro-1-naphthoic acid (3)**

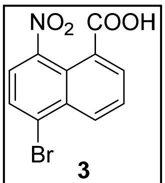

3

5-bromo-1-naphthoic acid, **2** (20.0 g, 79.7 mmol) was placed in a round bottom flask with acetic anhydride (200 mL), *p*-toluene sulfonic acid (4.0 g) and P_2O_5 (5.0 g). Fuming nitric acid (20.0 mL) was carefully added to this suspension, keeping the temperature below 30ºC. After 30 minutes the reaction was poured over water and ice. Once the acetic anhydride was hydrolysed (2 hours), the precipitate was filtered to yield, after drying, the nitro compound (**3**). This crude compound can be further purified by recrystallization from THF/EtOH, affording pure compound **3** in 86% yield (20.3 g) as a pale cream crystalline solid. m.p.: 211-214 ºC. IR (neat, ν in cm^{-1}): 3351, 2929, 2843, 1670, 1521, 1346. ^{1}H NMR (DMSO-*d*$_6$, 400 MHz): 8.50 (dd, 1H, J = 8.4 and 1.2 Hz), 8.20 (dd, 1H, J = 7.2 and 1.2 Hz,), 8.16 (ABq, 2H, J_{AB} = 8.4 Hz), 7.91 (dd, 1H, J = 8.4 and 7.2 Hz).^{13}C NMR (DMSO-*d*$_6$, 100 MHz): 167.6 (C), 146.8 (C), 132.3 (C), 131.8 (CH), 130.5 (CH), 130.0 (CH), 129.6 (C), 128.7 (CH), 128.5 (C), 125.4 (CH), 122.1 (C). HRMS (ESI) m/z: [M - H]$^-$ Calcd for $C_{11}H_5O_4N^{79}Br$ 293.9407; Found 293.9410.

- **5-chloro-8-nitro-1-naphthoyl chloride (4, NNapCl)**

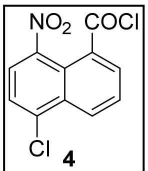

4

5-bromo-8-nitro-1-naphthoic acid (1.0 g, 3.38 mmol) was placed in a round bottom flask and thionyl chloride (5.0 mL) and a trace of pyridine were added. The round bottom flask was equipped with a condenser and a paraffin bubbler. The reaction mixture was stirred and heated in a water bath at 40ºC until a homogeneous solution was obtained. Once the acid was completely dissolved, the reaction was finished and the thionyl chloride was removed under vacuum. The crude acid chloride was used without further purification in the next step. Due to its instability, the acid chloride **4** is not characterized.

- **5-chloro-8-nitro-1-naphthoic acid (3a)**

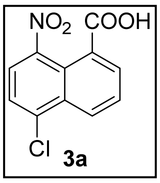

5-bromo-8-nitro-1-naphthoyl chloride **4** (500 mg, 1.85 mmol) was stirred in a 1:1 mixture of THF and water overnight. The solvent was evaporated under vacuum yielding the acid **3a** (440 mg, 95% yield) as a yellow crystalline solid. m.p.>200 ºC. IR (neat, ν in cm^{-1}) 3303, 2953, 1678, 1530, 1391, 730.^{1}H NMR (DMSO-d_6, 400 MHz,) δ 8.55 (dd, 1H, J = 8.5 and 1.2 Hz), 8.24 (d, 1H, J = 8.2 Hz), 8.23 (dd, 1H, J = 7.2 and 1.2 Hz, 1H), 8.00 (d, 1H, J = 8.2 Hz), 7.94(dd, 1H, J = 8.5 and 7.2 Hz).^{13}C NMR (DMSO-d_6, 100 MHz): 167.8 (C), 146.2 (C), 136.8 (C), 131.8 (CH), 131.1(C), 129.5 (C) 128.5 (CH), 127.7 (CH), 126.2 (CH), 125.3 (CH), 122.1 (C). HRMS (ESI) m/z: $[M - H]^-$ Calcd for $C_{11}H_5ClNO_4$ 249.9902; Found 249.9913.

2.2. General Procedure (A) for amine (5-11) protection using NNapCl

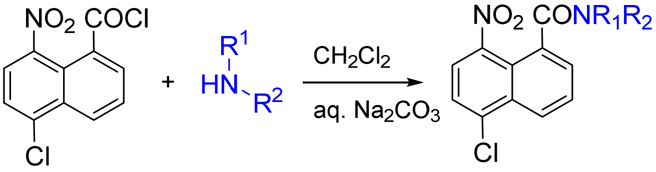

4 amines (**5-11**) amides **D** (**12-18**)

4 was prepared according to the above-described procedure by reacting 5-bromo-8-nitro-1-naphthoic acid **3** (500 mg; 1.69 mmol; 1 eq.) with thionyl chloride (5 ml; 69 mmol; 40 eq.). The crude acid chloride **4** was dissolved in methylene chloride (15 mL) and cooled down in an ice bath. Then, a solution of the amine (1.69 mmol; 1 eq.) dissolved in CH_2Cl_2 (3.0 mL) was added dropwise. After stirring for 5 minutes, a solution of Na_2CO_3 (8.45 mmol, 890 mg) in water (4 mL) was added and the mixture allowed to warm to room temperature, stirring for an additional 25 minutes. Water (20 mL) was then added, and the layers were separated in a separatory funnel. The organic layer was washed with HCl 2N and dried over anhydrous Na_2SO_4. The solvent was removed by vacuum and the crude residue was purified.

- **5-chloro-8-nitro-*N*-octyl-1-naphthamide (12)**

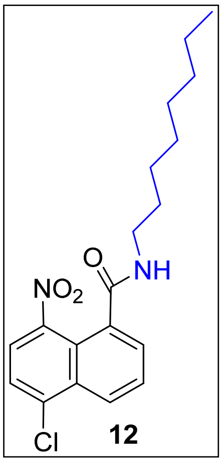

Purified by crystallization: 530 mg (86% yield) of a pale cream crystalline solid.

m.p. 182ºC-184ºC. IR (neat, ν in cm^{-1}) 3249, 3100,2919, 1519, 1424, 836, 759.^{1}H NMR ($CDCl_3$, 400 MHz): 8.47 (dd, 1H, J = 8.4 and 1.2 Hz,), 7.97 (d, 1H, J = 8.2 Hz,), 7.84 (dd, 1H, J = 7.2 and 1.2 Hz,), 7.68 (dd, 1H, J = 8.4 and 7.2 Hz), 7.67 (d, 1H, J = 8.2 Hz), 6.18 (m, 1H), 3.46 – 3.41 (m, 2H), 1.70 – 1.63 (m, 2H), 1.43 – 1.23 (m, 10H), 0.90 – 0.87 (m, 3H). ^{13}C NMR ($CDCl_3$, 100 MHz): 168.2 (C), 146.8 (C), 137.7 (C), 133.5 (C), 132.2 (C), 129.3 (CH), 127.5 (CH), 127.4 (CH), 125.5 (CH), 124.3 (CH), 123.0 (C), 40.3 (CH_2), 31.8 (CH_2), 29.5 (CH_2), 29.3 (CH_2), 29.2 (CH_2), 27.0 (CH_2), 22.6 (CH_2), 14.1 (CH_3). HRMS (ESI) m/z: $[M+H]^+$ Calcd for $C_{19}H_{24}O_3N_2Cl$ 363.1470; Found 363.1464.

- **5-chloro-*N*-decyl-8-nitro-1-naphthamide (13)**

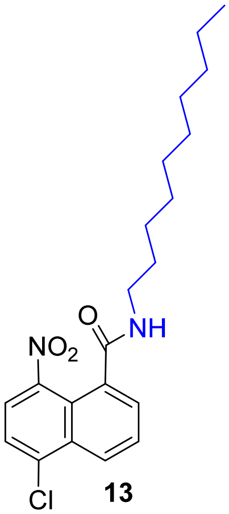

13

Purified by crystallization: 630 mg (95% yield) of a pale crystalline solid. m.p. 149ºC-151ºC. IR (neat, ν in cm^{-1}) 3243, 3095, 2921,1579,1465, 722. ^{1}H NMR ($CDCl_3$, 400 MHz): 8.47 (dd, 1H, J = 8.4 and 1.2 Hz,), 7.97 (d, 1H, J = 8.0 Hz,), 7.84 (dd, 1H, J = 7.0 and 1.2 Hz,), 7.68 (dd, 1H, J = 8.4 and 7.0 Hz), 7.67 (d, 1H, J = 8.0 Hz), 6.18 (m, 1H), 3.46 – 3.41 (m, 2H), 1.70 – 1.62 (m, 2H), 1.43 – 1.27 (m, 14H), 0.90 – 0.86 (m, 3H). ^{13}C NMR ($CDCl_3$, 100 MHz): 168.2 (C), 146.9 (C), 137.7 (C), 133.5 (C), 132.2 (C), 129.3 (CH), 127.5 (CH), 127.4 (CH), 125.5 (CH), 124.3 (CH), 122.9 (C), 40.3 (CH_2), 31.9 (CH_2), 29.6 (CH_2), 29.5 (CH_2), 29.5 (CH_2), 29.3 (CH_2), 29.3 (CH_2), 27.0 (CH_2), 22.7 (CH_2), 14.1 (CH_3). HRMS (ESI) m/z: $[M+H]^+$ Calcd for $C_{21}H_{28}O_3N_2Cl$ 391.1793; Found 391.1777.

- ***N*-benzyl-5-chloro-8-nitro-1-naphthamide (14)**

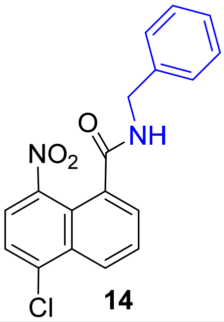

14

Purified by crystallization: 500 mg (87% yield) of a pale cream crystalline solid.
m.p.>200ºC IR (neat, ν in cm^{-1}) 3257, 3089, 1530, 1357, 845, 756, 700. ^{1}H NMR (DMSO-d_6, 400 MHz): 9.37 (t, 1H, J = 5.6 Hz), 8.48 (dd, 1H, J = 8.4 and 1.2 Hz,), 8.17 (d, 1H, J = 8.4 Hz,), 8.03 (dd, 1H, J = 7.2 and 1.2 Hz), 7.95 (d, 1H, J = 8.4 Hz), 7.91 (dd, 1H, J = 8.4 and 7.2 Hz), 7.41 (d, 2H, J =7.2Hz), 7.35 (t, 2H, J = 7.2 Hz), 7.26 (t, 1H J = 7.2 Hz), 4.46 (d, 2H, J = 5.6 Hz). ^{13}C NMR (DMSO-d_6, 100 MHz): 167.3 (C), 146.7 (C), 139.1 (C), 136.4 (C), 132.8 (C), 131.1 (C), 130.5 (CH), 128.4 (CH), 128.3 (2CH), 127.5 (2CH), 126.9 (CH), 126.6 (CH), 126.0 (CH), 124.8 (CH), 122.1 (C), 42.6 (CH_2). HRMS (ESI) m/z: $[M+H]^+$ Calcd for $C_{18}H_{14}O_3N_2Cl$ 341.0688; Found 341.0683.

- **5-chloro-8-nitro-*N*-(2,4,4-trimethylpentan-2-yl)-1-naphthamide (15)**

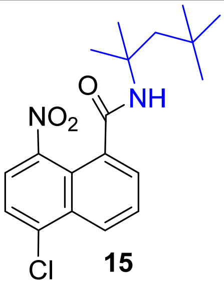

15

Purified by crystallization: 550 mg (90% yield) of a pale cream powder. m.p.=150ºC-152ºC. IR (neat, ν in cm^{-1}) 3306, 3066, 2953, 1539, 1351, 836, 753. ^{1}H NMR ($CDCl_3$, 400 MHz): 8.44 (dd, 1H, J = 8.4 and 1.2 Hz,), 7.96 (d, 1H, J = 8.4 Hz,), 7.80 (dd, 1H, J = 7.2 and 1.2 Hz), 7.65 (d, 1H, J = 8.4 Hz), 7.65 (dd, 1H, J = 8.4 and 7.2 Hz), 6.07 (s, 1H), 1.83 (s, 2H), 1.57 (s, 6H), 1.09 (s, 9H). ^{13}C NMR ($CDCl_3$, 100 MHz): 166.8 (C), 147.0 (C), 137.5 (C), 134.7 (C), 132.3 (C), 128.9 (CH), 127.4 (CH), 127.1 (CH), 125.4 (CH), 124.1 (CH), 123.0 (C), 56.4 (C), 52.8 (CH_2), 31.7 (C), 31.6 ($2CH_3$) 28.7 ($3CH_3$). HRMS (ESI) m/z: $[M+H]^+$ Calcd for $C_{19}H_{24}O_3N_2Cl$ 363.1470; Found 363.1465.

- ***N,N*-dibutyl-5-chloro-8-nitro-1-naphthamide (16)**

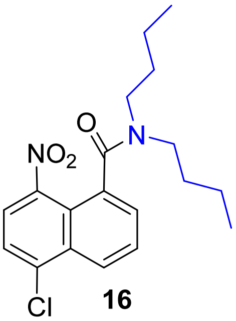
16

Purified by chromatography: 530 mg (87% yield) of a deep orange oil. IR (neat, ν in cm^{-1}) 3754, 2961, 2872, 1633, 1465, 762. ^{1}H NMR ($CDCl_3$, 400 MHz): 8.45 (dd, 1H, J = 8.4 and 1.2 Hz,), 7.92 (d, 1H, J = 8.0 Hz,), 7.73 – 7.63 (m, 3H), 3.58 – 3.31 (m, 4H), 1.82 – 1.64 (m, 4H), 1.47 – 1.36 (m, 2H), 1.31 – 1.21 (m, 2H), 0.99 (t, 3H, J = 7.2 Hz) 0.89 (t, 3H, J = 7.2 Hz).^{13}C NMR ($CDCl_3$, 100 MHz): 169.5 (C), 146.7 (C), 137.7 (C), 133.0 (C), 132.4 (C), 128.8 (CH), 127.2 (CH), 126.4 (CH), 125.2 (CH), 123.9 (CH), 123.5 (C), 49.7 (CH_2), 44.8 (CH_2), 30.4 (CH_2), 29.2 (CH_2), 20.5 (CH_2), 20.2 (CH_2), 14.0 (CH_3), 13.8 (CH_3). HRMS (ESI) m/z: $[M+H]^+$ Calcd for $C_{19}H_{24}O_3N_2Cl$ 363.1470; Found 363.1463.

- ***N*-(4-(*tert*-butyl)phenyl)-5-chloro-8-nitro-1-naphthamide (17)**

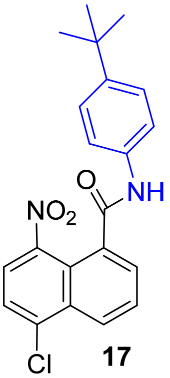
17

Purified by crystallization: 586 mg (91% yield) of an orange crystalline solid. m.p.>200 ºC. IR (neat, ν in cm^{-1}) 3749, 2961, 1595, 1527, 1348, 836. ^{1}H NMR ($CDCl_3$, 400 MHz): 8.49 (d, 1H, J = 8.4 Hz), 8.01 (d, 1H, J = 8.4 Hz), 7.96 (d, 1H, J = 7.2 Hz), 7.91 (broad s, 1H), 7.72 – 7.68 (m, 2H), 7.54 (d, 2H, J = 8.4 Hz), 7.38 (d, 2H, J = 8.4 Hz), 1.33 (s, 9H). ^{13}C NMR ($CDCl_3$, 100 MHz): 166.0 (C), 148.0 (C), 146.7 (C), 137.9(C), 134.8 (C), 133.4 (C), 132.1 (C), 129.3 (CH), 127.7 (CH), 127.5 (CH), 126.0 (2CH), 125.7 (CH), 124.6 (CH), 122.8 (C), 120.4 (2CH), 34.4 (C), 31.4 ($3CH_3$). HRMS (ESI) m/z: $[M+H]^+$ Calcd for $C_{21}H_{20}O_3N_2Cl$ 383.1157; Found 383.1150.

- **5-chloro-*N*-((1*S*,2*S*)-2-hydroxy-2,3-dihydro-1*H*-inden-1-yl)-8-nitro-1-naphthamide (18)**

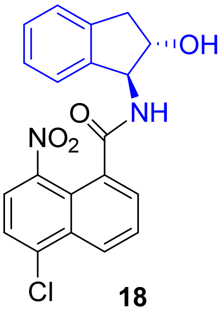
18

Purified by crystallization: 550 mg (86% yield) of a yellow powder. m.p.>200 ºC. IR (neat, ν in cm^{-1}) 3502, 3285 ,2920, 1737 ,1634 ,1514, 1353, 1277, 1079. ^{1}H NMR ($CDCl_3$, 400 MHz): 8.56 (dd, 1H, J = 8.4 and 1.2 Hz), 8.05 (d, 1H, J = 8.2 Hz), 7.99 (dd, 1H, J = 7.2 and 1.2 Hz), 7.76 (dd, 1H, J = 8.4 and 7.2 Hz), 7.74 (d, 1H, J = 8.2 Hz), 7.38 – 7.36 (m, 1H), 7.32 – 7.28 (m, 3H), 6.57 (d, 1H, J = 6.4 Hz), 5.40 (t, 1H, J = 6.4 Hz), 4.69 (td, 1H, J = 8.0 and 6.4 Hz), 3.38 (dd, 1H, J = 16 and 8.0 Hz), 3.03 (dd, 1H, J = 16 and 8.0 Hz). ^{13}C NMR ($CDCl_3$, 100 MHz): 170.0 (C), 146.6 (C), 140.8 (C), 139.0 (C), 138.2 (C), 132.4 (C), 132.3 (C), 129.6 (CH), 128.8 (CH), 128.1 (CH), 127.5 (CH), 127.3 (CH), 125.8 (CH), 125.4 (CH), 124.7 (CH), 123.1 (CH), 123.0 (C), 81.6 (CH), 64.3 (CH), 38.3 (CH_2). HRMS (ESI) m/z: $[M+H]^+$ Calcd for $C_{20}H_{16}O_4N_2Cl$ 383.0793; Found 383.0792. $[\alpha]_D^{20}$ +78.4 (c 0.25, MeCN).

2.3. Procedure for deprotection

Removal of the protective group was explored using a variety of reducing conditions: Zn/AcOH, $SnCl_2$/MeOH, and H_2/Pd (C)/AcOEt. Next, the different procedures are described for amide **16**. Reduction of amines (**12**, **15**, **17** and **18**) proceeded under similar conditions. Lactam **F** was purified by column chromatography on silica gel using 100% CH_2Cl_2 to a mixture CH_2Cl_2: EtOAc (9:1).

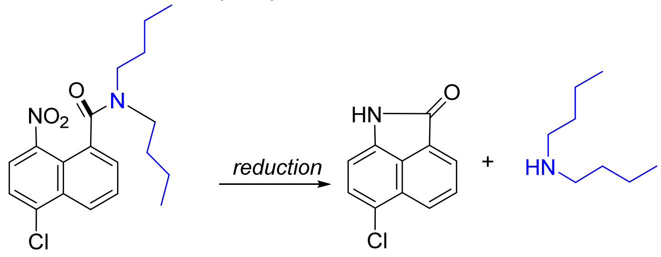

16   *lactam* **F**   **9**

- **Zn/AcOH (B)**

A solution of the amide **16** (300 mg, 0.83 mmol, 1 eq.) in acetic acid (3 mL) was added to an Erlenmeyer flask containing a preheated (60ºC) and well-stirred suspension of Zn (3.0 g, 46 mmol, 55 eq.) in acetic acid (6 mL). The reaction mixture was stirred for 10 minutes and ethyl acetate (20 mL) and water (20 mL) were then added. Zn was removed by filtration (or careful decantation), the two phases separated, and the organic layer acidified with 2N HCl. The acidic aqueous layer and the ethyl acetate layer (containing the lactam **F**) were separated, and the acidic extract basified with solid Na_2CO_3. The basic aqueous layer was finally extracted with ethyl acetate, the combined organic layers dried over anhydrous Na_2SO_4, filtered, and the ethyl acetate was evaporated under reduced pressure, to afford di-*n*-butylamine **9** in 95% yield (160 mg).

- **$SnCl_2$/MeOH (C)**

The amide **16** (140 mg, 0.39 mmol, 1 eq.) was dissolved in methanol (10 mL) and excess of $SnCl_2$ was added (300 mg, 1.58 mmol, 4 eq.). The reaction mixture was heated in a water bath at 60ºC for 10 minutes. Next, 2.0 mL of an aqueous 2M Na_2CO_3 solution, solid Na_2CO_3 (500 mg, 4.72 mmol) and ethyl acetate (20 mL) were added, and the reaction mixture was stirred for additional 10 minutes, until a basic pH was obtained. The reaction mixture was filtered, the phases separated, and the combined organic layers acidified with 2N HCl. Lactam **F** could be isolated from the organic layer, after drying over anhydrous Na_2SO_4, filtering and evaporating the solvent under reduced pressure. The acidic extracts (containing the protonated amine) were basified with solid Na_2CO_3 and extracted with ethyl acetate, dried over anhydrous Na_2SO_4, filtered, and evaporated under reduced pressure to afford di-*n*-butylamine **9** in 80% yield (63 mg).

- **H_2/Pd (C)/ EtOAc (D)**

The amide **16** (140 mg, 0.39 mmol, 1eq.) was dissolved in ethyl acetate (7.0 mL). Then 100 mg of Pd/C 5% was added and the mixture was reacted with hydrogen under pressure (3.5 atm) overnight at 20ºC. The catalyst was removed by filtration and the solvent was evaporated under reduced pressure, but lactam cyclization was incomplete according to ^{1}H NMR spectrum. The crude residue was then dissolved in acetic acid and heated for 2 minutes at 100ºC to complete the amine liberation. The reaction mixture was worked up with ethyl acetate and water as described previously to afford di-*n*-butylamine **9** in 90% yield (71 mg).

- **6-chlorobenzo[*cd*]indol-2(1H)-one (F)**

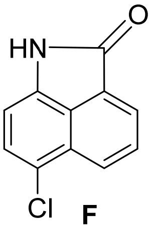

F

Orange crystalline solid

m.p.> 200ºC. IR (neat, ν in cm^{-1}) 3755, 2927, 2859, 2369, 2338, 1354. ^1H NMR (CD_3OD, 400 MHz): 8.26 (dd, 1H, J = 8.4 and 0.4 Hz), 8.10 (d, 1H, J = 6.8 Hz,), 7.88 (dd, 1H, J = 8.4 and 6.8 Hz), 7.51 (d, 1H, J = 7.2 Hz), 6.94 (d, 1H, J = 7.2 Hz). ^{13}C NMR (CD_3OD, 100 MHz): 171.4 (C), 138.4 (C), 130.9 (CH), 129.4 (CH), 129.3 (CH), 129.0 (C), 128.7 (C), 128. 3 (C), 126.1 (CH), 125.6 (C), 108.5 (CH). HRMS (ESI) m/z: $[M+H]^+$ Calcd for $C_{11}H_7ONCl$ 204.0210; Found 204.0210.

2.4. Capture of the intermediate amine

Amide **16** (0.83 mmol, 300 mg) in acetic acid (3 mL) was added to a suspension of Zn (46 mmol, 3.0 g) in acetic acid (6 mL). The reaction mixture was stirred for 10 minutes. Then, acetic anhydride (10 mL) was added, and the reaction mixture was stirred at room temperature until acetylation was complete. Ethyl acetate (20 mL) and 4% (w/v) aqueous solution of Na_2CO_3 (20 mL) were then added and Zn was removed by filtration (or careful decantation). The two phases were separated, and the organic layer dried over anhydrous Na_2SO_4 and filtered. The solvent was evaporated under reduced pressure and the crude residue was purified by silica gel column chromatography using CH_2Cl_2: EtOAc (95:5) to afford 255 mg (82%) of the desired compound **Ac16**.

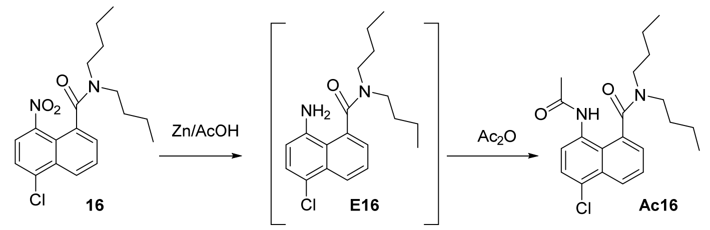

- **8-acetamido-*N,N*-dibutyl-5-chloro-1-naphthamide (Ac16)**

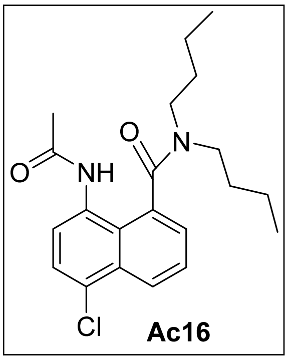

^{1}H NMR ($CDCl_3$, 400 MHz): 8.75 (s, 1H), 8.31 (dd, 1H, J = 8.4 and 1.2 Hz), 7.69 (d, 1H, J = 8.0 Hz), 7.55 (d, J = 8.0 Hz, 1H), 7.50 (dd, 1H, J = 8.4 and 7.2 Hz), 7.28 (dd, 1H, J = 7.2 and 1.2 Hz,), 4.13 – 4.01 (m, 1H), 3.08 – 2.99 (m, 1H), 2.98 – 2.81 (m, 2H), 2.13 (s, 3H), 1.82 – 1.56 (m, 2H), 1.48– 1.35 (m, 2H), 1.35 – 1.11 (m, 2H), 0.97 (t, 3H, J = 7.2 Hz), 0.92 – 0.83 (m, 2H), 0.53 (t, 3H J = 7.2 Hz). ^{13}C NMR ($CDCl_3$, 100 MHz): 173.8 (C), 168.7 (C), 131.8 (2C), 131.6 (C), 129.5 (C), 126.8 (CH), 126.5 (CH), 126.5 (CH), 125.8 (CH), 125.2 (C), 124.4 (CH), 49.6 (CH_2), 45.3 (CH_2), 30.3 (CH_2), 30.1 (CH_2), 24.1(CH_3), 20.4 (CH_2), 19.6 (CH_2), 13.9 (CH_3), 13.3 (CH_3). HRMS (ESI) m/z: $[M+H]^+$ Calcd for $C_{21}H_{28}O_2N_2Cl$ 375.1834; Found 375.1831.

2.5. Synthesis de H-Leu-Leu-O*t*Bu

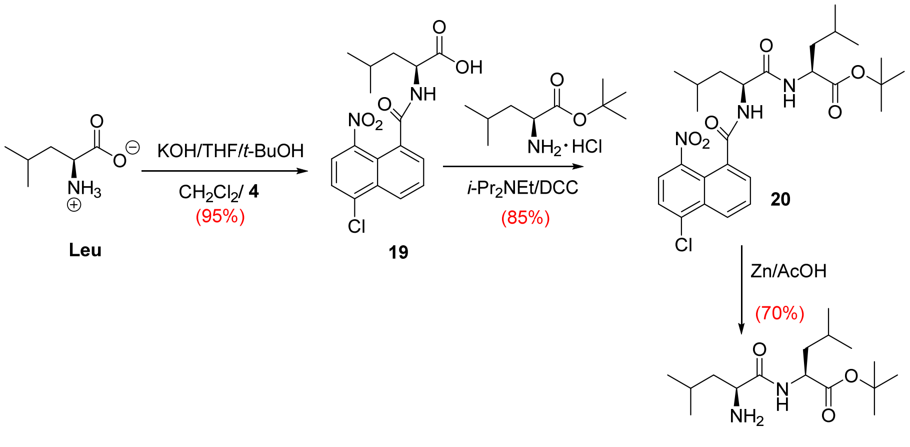

- **(5-chloro-8-nitro-1-naphthoyl)-L-leucine (19)**

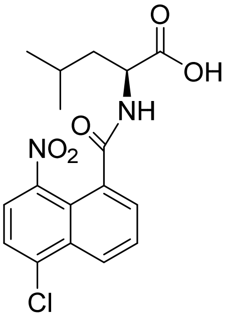

19

The acid chloride **4** was prepared according to the described procedure by reacting 5-bromo-8-nitro-1-naphthoic acid **3** (500 mg, 1.69 mmol) with thionyl chloride (5 mL). Meanwhile, L-Leucine (540 mg, 4.12 mmol) was suspended in 10 mL of a tetrahydrofuran-*tert*-butanol (1:1) mixture containing potassium hydroxide (230 mg, 4.12 mmol) and ultrasonic treatment was applied until total dissolution. Next, the leucine solution was added to a solution of the acid chloride **4** in CH_2Cl_2 (10 mL) at 0ºC and the reaction was stirred for 15 minutes. The reaction mixture was then poured onto a 2N HCl solution and extracted with CH_2Cl_2, dried over anhydrous Na_2SO_4, filtered, and evaporated under reduced pressure to affording the protected L-leucine **19** as a cream solid (585 mg, 95%). No further purification was required.

m.p.= 142ºC-144ºC. IR (neat, ν in cm^{-1}) 3294, 2961, 1724, 1641, 1530, 1350, 765. ^1H NMR ($CDCl_3$, 400 MHz): 8.50 (d, 1H, J = 8.4 Hz,), 7.99 (d, 1H, J = 8.0 Hz), 7.95 (d, 1H, J = 6.8 Hz), 7.73 – 7.68 (m, 1H), 7.69 (d, 1H, J = 8.0 Hz), 6.60 (d, 1H, J = 8.0 Hz), 4.86 (td, 1H, J = 8.4 and 5.2 Hz), 1.98 – 1.74 (m, 3H), 1.06 (d, 3H, J = 6.4 Hz), 1.03 (d, 3H, J = 6.4 Hz). ^{13}C NMR ($CDCl_3$, 100 MHz): 176.1 (C), 168.0 (C), 146.7 (C), 137.9 (C), 132.5 (C), 132.2 (C), 129.8 (CH), 127.9 (CH), 127.5 (CH), 125.7(CH), 124.5 (CH), 122.8 (C), 51.1 (CH), 41.4 (CH_2), 24.8 (CH), 22.8 (CH_3), 21.6 (CH_3). HRMS (ESI) m/z: $[M+Na]^+$ Calcd for $C_{17}H_{17}O_5N_2ClNa$ 387.0718; Found 387.0713. $[\alpha]_D^{20}$ - 44.3 (c 1, $CHCl_3$)

- ***tert*-butyl (5-chloro-8-nitro-1-naphthoyl)-L-leucyl-L-leucinate (20)**

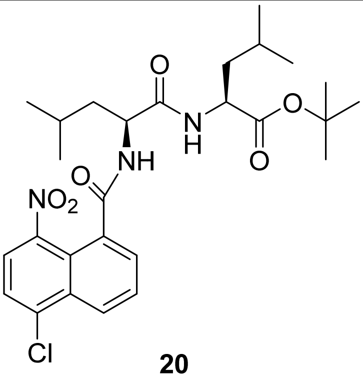

20

The protected L-leucine **19** (105 mg, 0.30 mmol), H-Leu-O*t*Bu• HCl (67 mg, 0.30 mmol) and *i*-Pr_2NEt (51 μL, 0.30 mmol) were dissolved in methylene chloride (10.0 mL) under stirring. Then DCC (64 mg, 0.30 mmol) was added, and the reaction mixture was stirred at room temperature for 30 minutes. The dicyclohexyl urea was removed by filtration, and the reaction mixture was poured into a dilute solution of 2N HCl. The two phases were separated, the combined organic layers dried over anhydrous Na_2SO_4, filtered, and concentrated under reduced pressure. The crude residue was purified by silica gel column chromatography using methylene chloride/ethyl acetate (9:1 to 7:3) to afford 135 mg of the desired dipeptide **20** as a brown oil in 85% yield.

IR (neat, ν in cm^{-1}) 3280, 2958, 1638, 1536, 1351, 1155. ^1H NMR ($CDCl_3$, 400 MHz): 8.51 (dd, 1H, J = 8.4 and 1.2 Hz,), 7.99 (d, 1H, J = 8.0 Hz,), 7.90 (dd, 1H, J = 7.2 and 1.2 Hz), 7.71 (dd, 1H, J =8.4 and 7.2 Hz), 7.70 (d, 1H, J =8.0 Hz), 6.70 (d, 1H, J = 8.4 Hz), 6.30 (d, 1H, J = 8.4 Hz), 4.66 (td, 1H, J = 8.4 and 5.9 Hz), 4.50 (td, 1H, J = 8.4 and 5.7 Hz), 1.90 – 1.76 (m, 6H), 1.47 (s, 9H), 1.03 (d, 3H, J = 6.0 Hz), 1.02 (d, 3H, J = 6.0 Hz), 0.92 (t, 6H, J = 6.0 Hz). ^{13}C NMR ($CDCl_3$, 100 MHz): 171.6 (C), 171.3 (C), 167.9 (C), 146.8 (C), 137.8 (C), 132.7 (C), 132.2 (C), 129.6 (CH), 127.8 (CH), 127.5 (CH), 125.6 (CH), 124.4 (CH), 122.8 (C), 81.8 (C), 52.0 (CH), 51.6 (CH), 41.5 ($2CH_2$), 27.9 ($3CH_3$), 24.8 (2CH), 23.0 (CH_3), 22.7 (CH_3), 22.3 (CH_3), 22.0 (CH_3). HRMS (ESI) m/z: $[M+Na]^+$ Calcd for $C_{27}H_{36}O_6N_3ClNa$ 556.2184; Found 556.2183. $[\alpha]_D^{20}$ - 71.3 (c 1, $CHCl_3$).

- ***tert*-butyl L-leucyl-L-leucinate (H-Leu-Leu-O*t*Bu)**

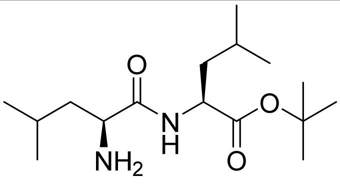

H-Leu-Leu-O*t*Bu

The protected dipeptide **20** (106 mg, 0.20 mmol) was dissolved in acetic acid (1.0 mL) and added to a preheated and well-stirred suspension of Zn (1.0 g) in AcOH (3.0 mL). Following the above-described procedure for deprotection (B), 45 mg of the dipeptide H-Leu-Leu-O*t*Bu was obtained as a colourless oil in 70% yield. The spectroscopic properties agreed with those published [11].

^1H NMR ($CDCl_3$, 400 MHz): 7.58 (d, 1H, J = 8.0 Hz), 4.48 (dt, 1H, J = 8.8 and 5.2 Hz) 3.40 (d, 1H, J = 8.0 Hz), 1.76-1.22 (m, 8H), 1.45 (s, 9H), 0.97-0.92 (m, 12H).^{13}C NMR ($CDCl_3$, 100 MHz):175.1 (C), 172.4 (C), 81.8 (C), 53.5(CH), 50.8 (CH), 44.1 (CH_2), 41.7 (CH_2), 28.0 ($3CH_3$), 25.0 (CH), 24.9 (CH), 23.4 (CH_3), 22.9 (CH_3), 22.1 (CH_3), 21.3 (CH_3). HRMS (ESI) m/z: $[M+H]^+$ Calcd for $C_{16}H_{33}O_3N_2$ 301.2486; Found 301.2480. $[\alpha]_D^{20}$ -21.5 (c 1, $CHCl_3$).

2.6. Orthogonal protection of lysine

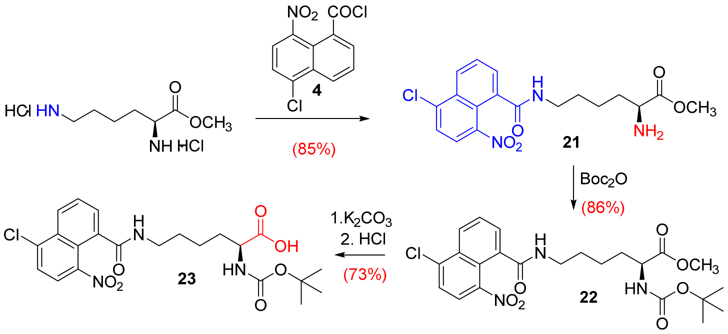

- **Methyl N^6-(5-chloro-8-nitro-1-naphthoyl)-L-lysinate (21)**

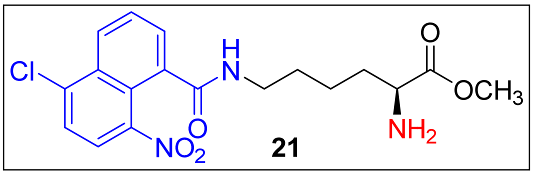

H-Lys-OMe•2HCl (2.20 g, 10.2 mmol) was suspended in a dichloromethane-*tert*-butanol (5:1) solvent mixture (60 mL) containing *i*-Pr_2Et (7.0 mL, 40.1 mmol) and stirred at room temperature until total dissolution. Next, a solution of the acid chloride **4** (920 mg, 3.4 mmol) in CH_2Cl_2 (10 mL) was added. The reaction mixture was stirred for 10 minutes until it was completed. The reaction mixture was worked-up, extracting with ethyl acetate (50 mL) and aqueous sodium carbonate 4% (w/v). The combined organic layers were dried over anhydrous Na_2SO_4, filtered, and concentrated under reduced pressure. The crude residue (1.3 g) was purified by crystallization to yield 1.1 g of the desired compound **21** in 85% yield as a pale yellow crystalline solid.

m.p. = 155ºC-157ºC. IR (neat, ν in cm^{-1}) 3260, 2952, 1636, 1533, 1351, 1179. ^{1}H NMR (CD_3OD, 400 MHz): 8.55 (dd, 1H, J = 8.4 and 1.2 Hz), 8.08 (d, 1H, J = 8.0 Hz,), 7.97 (dd, 1H, J = 7.2 and 1.2 Hz), 7.83 (dd, 1H, J = 8.4 and 7.2 Hz), 7.83 (d, 1H, J = 8.0 Hz), 4.08 (t, 1H, J = 6.4 Hz), 3.83 (s, 3H), 3.37 (t, 2H, J = 6.8 Hz), 2.08 – 1.91 (m, 2H), 1.76 – 1.68 (m, 2H), 1.67 – 1-49 (m, 2H).^{13}C NMR (CD_3OD, 100 MHz): 171.0 (C), 170.9 (C),148.3 (C), 138.6 (C), 134.1 (C), 133.2 (C), 131.5 (CH), 129.1 (CH), 128.3 (CH), 127.0 (CH), 125.8 (CH), 123.9 (C), 53.9 (CH_3), 53.7 (CH), 40.40 (CH_2), 31.2 (CH_2), 39.8 (CH_2), 23.4 (CH_2). HRMS (ESI) m/z: [M+H]$^+$ Calcd for $C_{18}H_{21}O_5N_3Cl$ 394.1164; Found 394.1161. $[\alpha]_D^{20}$ +7.9 (c 1, MeOH).

- **Methyl *N*^{2}-(*tert*-butoxycarbonyl)-*N*^{6}-(5-chloro-8-nitro-1-naphthoyl)-L-lysinate (22)**

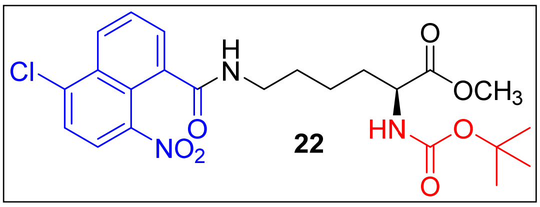

The lysine derivative **21** (1.30 g, 3.30 mmol) was dissolved in CH_2Cl_2 (30 mL) and a solution of Boc_2O (720 mg, 3.30 mmol) in 5 mL CH_2Cl_2 was added. The reaction mixture was stirred for 20 minutes and the solvent was removed under vacuum. The crude residue was purified by column chromatography on silica gel using CH_2Cl_2: AcOEt mixtures of increasing polarity (9:1 to 7:3) to afford 1.4 g (86% yield) of the desired compound **22** as a yellow powder.

IR (neat, ν in cm^{-1}) 3306, 2981, 1644, 1528, 1359, 1211, 1169. ^{1}H NMR (CD_3OD, 400 MHz): 8.49 (dd, 1H, J = 8.4 and 1.2 Hz), 8.02 (d, 1H, J = 8.0 Hz,), 7.89 (dd, 1H, J = 7.2 and 1.2 Hz), 7.84 – 7.78 (m, 2H), 4.09 (dd, 1H, J = 8.8 and 5.2 Hz), 3.67 (s, 3H), 3.28 – 3.25 (m, 2H), 1.85 – 1.71 (m, 1H), 1.55 – 1.43 (m, 3H), 1.85 – 1.71 (m, 2H), 1.37 (s, 9H). ^{13}C NMR (CD_3OD, 100 MHz): 175.0 (C), 171.0 (C),158.2 (C), 148.3 (C), 138.4 (C), 134.4 (C), 133.4 (C), 131.6 (CH), 129.3 (CH), 128.2 (CH), 127.0 (CH), 125.7 (CH), 123.9 (C), 80.4 (C), 55.1 (CH), 52.6 (CH_3), 40.5 (CH_2), 31.4 (CH_2), 29.6 (CH_2), 28.7 ($3CH_3$), 24.3 (CH_2). HRMS (ESI) m/z: $[M+Na]^+$ Calcd for $C_{23}H_{28}O_7N_3ClNa$ 516.1508; Found 516.1506. $[\alpha]_D^{20}$ -31 (c 0.5, $CHCl_3$).

- ***N*²-(*tert*-butoxycarbonyl)-*N*⁶-(5-chloro-8-nitro-1-naphthoyl)-L-lysine (23)**

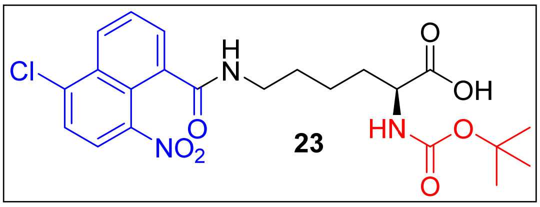

The methyl ester **22** (210 mg, 0.43 mmol) was dissolved in methanol (5 mL) containing water (500 mg). Potassium carbonate (210 mg, 1.52 mmol) was added, and the reaction mixture was heated in a water bath at 35ºC for 4 hours. The reaction mixture was then poured on 2N HCl and a solid precipitated. The solid product was then filtered and recrystallized to give 150 mg of the compound **23** in 73% yield as a pale yellow crystalline solid.

^{1}H NMR (CD_3OD, 400 MHz): 8.54 (dd, 1H, J = 8.4 and 1.2 Hz), 8.07 (d, 1H, J = 8.0 Hz,), 7.94 (dd, 1H, J = 7.2 and 1.2 Hz), 7.86 – 7.78 (m, 2H), 4.12 – 4.08 (m, 1H), 3.34 (t, 2H, J = 6.8 Hz), 3.28 – 3.25 (m, 2H), 1.92 – 1.47 (m, 6H), 1.42 (s, 9H). ^{13}C NMR (CD_3OD, 100 MHz): 176.3 (C), 170.9 (C),158.2 (C), 148.3 (C), 138.5 (C), 134.3 (C), 133.2 (C), 131.4 (CH), 128.9 (CH), 128.0 (CH), 127.1 (CH), 125.7 (CH), 123.8 (C), 80.45 (C), 54.9 (CH), 40.8 (CH_2), 32.6 (CH_2), 29.8 (CH_2), 28.6 ($3CH_3$), 24.6 (CH_2). HRMS (ESI) m/z: $[M+Na]^+$ Calcd for $C_{22}H_{26}O_7N_3{}^{35}Cl^{23}Na$ 502.1352; Found 502.1347. $[\alpha]_D^{20}$ -34.6 (c 1, $CHCl_3$).

2.7. Reaction with 1-amino-2-indanol (11)

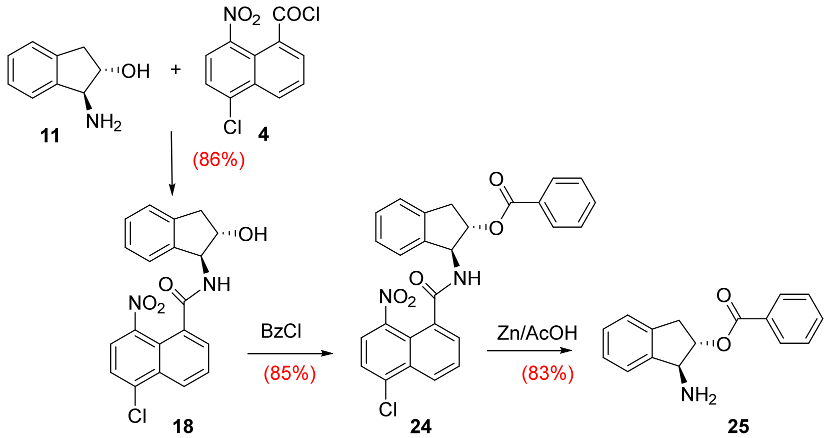

- **(1*S*,2*S*)-1-(5-chloro-8-nitro-1-naphthamido)-2,3-dihydro-1*H*-inden-2-yl benzoate (24)**

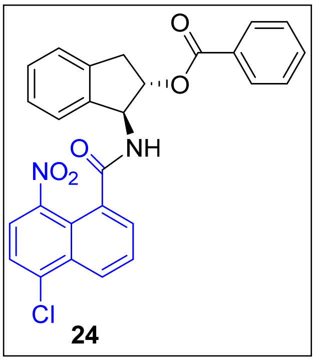

Compound **18** (500 mg, 1.31 mmol) was dissolved in a mixture of benzoyl chloride (155 µL, 1.31 mmol) and pyridine (4 mL) and the reaction mixture was stirred vigorously for 1 hour at room temperature. Then, water (around 5 drops) was added and stirring was maintained. The reaction mixture was treated with ethyl acetate (40 mL) and 2N HCl (50 mL) and the two phases were separated. The organic layer was dried over anhydrous Na_2SO_4, filtered, and the solvent evaporated under reduced pressure. The crude residue was purified by silica gel column chromatography, with methylene chloride: diethyl ether (99:1) as eluent, to afford 540 mg (85% yield) of the compound **24** as a white powder.

m.p.>200ºC. IR (neat, ν in cm^{-1}) 3261, 1722, 1642 ,1517, 1462, 1353 ,1269 ,1114 .^1H NMR (DMSO-d_6, 400 MHz): 9.50 (d, 1H, J = 8.0 Hz), 8.49 (dd, 1H, J = 8.0 and 1.2 Hz), 8.17 (d, 1H, J = 8.0 Hz), 8.07 (dd, 1H, J = 7.2 and 1.2 Hz,), 8.02 (dd, 2H, J = 8.4 and 1.2 Hz), 7.95 (d, 1H, J = 8.0 Hz), 7.92 (dd, 1H, J = 8.0 and 7.2 Hz), 7.68 – 7.63 (m, 1H), 7.53 – 7.48 (m, 3H) 7.34 – 7.30 (m, 3H), 5.72 – 5.64 (m, 2H), 3.57 (dd, 1H, J = 16 and 6.8 Hz), 3.05 (dd, 1H, J = 16 and 6.8 Hz). ^{13}C NMR (DMSO-d_6, 100 MHz): 167.2 (C), 165.7 (C), 146.6 (C), 140.0 (C), 139.0 (C), 136.4 (C), 133.3 (CH), 132.6 (C), 131.1 (C), 130.7 (CH), 129.6 (C), 129.3 (2CH), 128.6 (2CH), 128.3 (CH), 128.3 (CH), 127.2 (CH), 126.7 (CH), 126.0 (CH), 124.9 (CH), 124.8 (CH), 124.3 (CH), 122.1 (C), 80.4 (CH), 58.5 (CH), 36.1 (CH_2). HRMS (ESI) m/z: $[M+H]^+$ Calcd for $C_{27}H_{20}O_5N_2Cl$ 487.1055; Found 487.1055. $[\alpha]_D^{20}$ +11.6 (c 1, $CHCl_3$).

- **(1*S*,2*S*)-1-amino-2,3-dihydro-1*H*-inden-2-yl benzoate (25)**

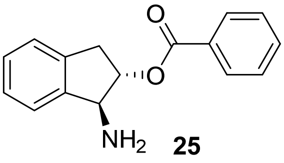

25

Compound **24** (90 mg, 0.19 mmol) was dissolved in acetic acid (3.0 mL) and a preheated (60ºC) suspension of Zn (1.0 g) in acetic acid (4.0 mL) was added under vigorous stirring. The reaction mixture was stirred for 20 minutes, and the Zn removed by filtration or careful decantation. Following the above-described procedure for deprotection B, 40 mg of the compound **25** was obtained in 83% yield.

^{1}H NMR ($CDCl_3$, 400 MHz): 7.88 (dd, 2H J = 8.4 and 1.2 Hz), 7.38 (t, 1H, J = 7.6 Hz), 7.29 – 7.19 (m, 3H), 7.13 – 7.03 (m, 3H), 5.17 – 5.11 (m, 1H), 4.35 (d, 1H, J = 5.2 Hz), 3.38 (dd, 1H, J = 16.4 and 7.6 Hz), 2.83 (dd, 1H, J = 16.4 and 6.4 Hz). ^{13}C NMR ($CDCl_3$,, 100 MHz): 166.8 (C), 143.1 (C), 138.9 (C), 133.0 (CH), 130.0 (C), 129.6 (2CH), 128.3 (2CH), 128.1 (CH), 127.3 (CH), 124.8 (CH), 124.0 (CH), 84.7 (CH), 62.4 (CH), 36.4 (CH_2). HRMS (ESI) m/z: $[M+H]^+$ Calcd for $C_{16}H_{16}O_2N$ 254.1176; Found 254.1174. $[\alpha]_D^{20}$ +20.5 (c 1, $CHCl_3$).

2.8. NMR spectra

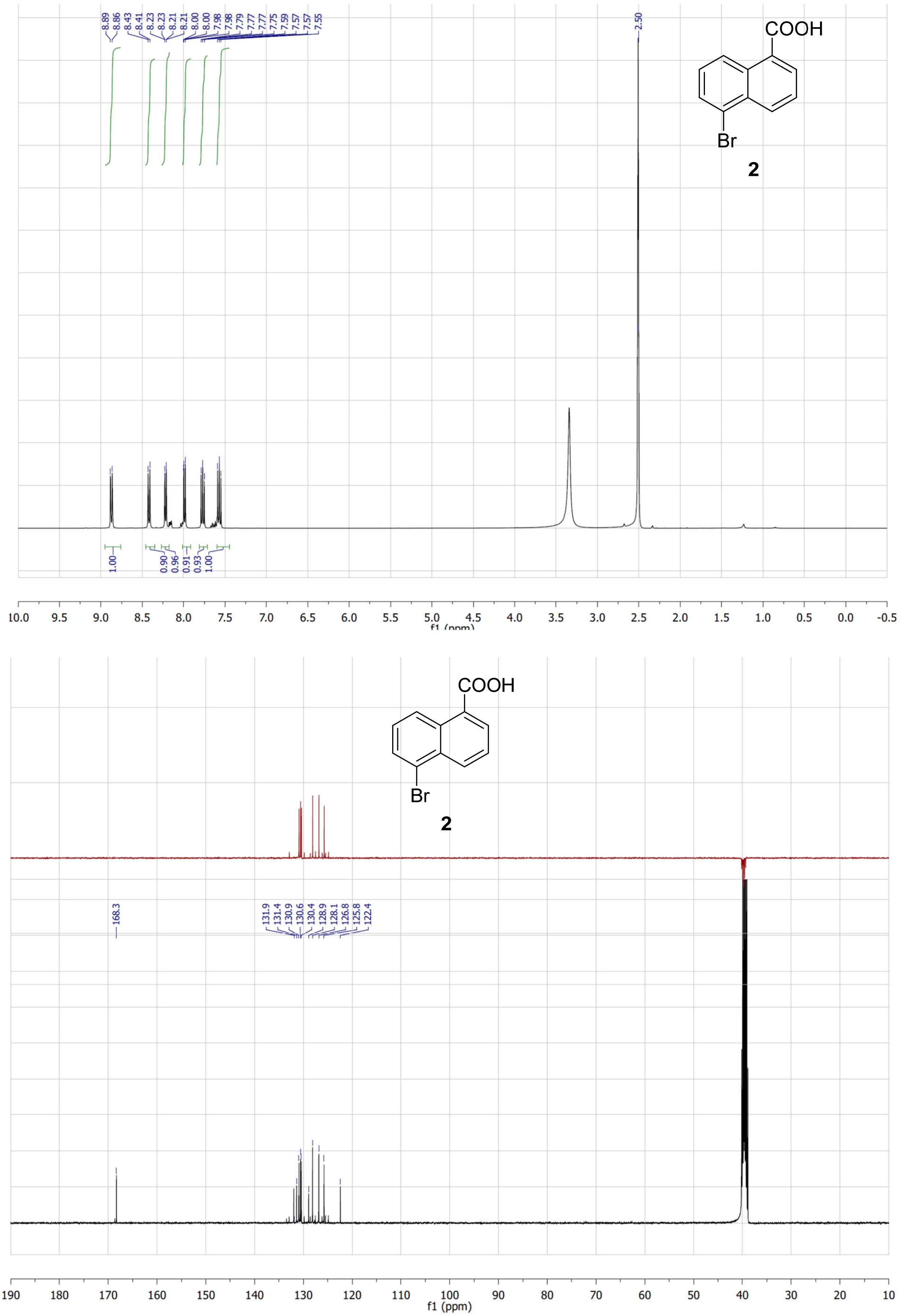

Figure S1. *^{1}H* NMR (*DMSO-d_6*, 400 MHz) *and ^{13}C* (*DMSO-d_6*, 100 MHz) *spectra of* (**2**).

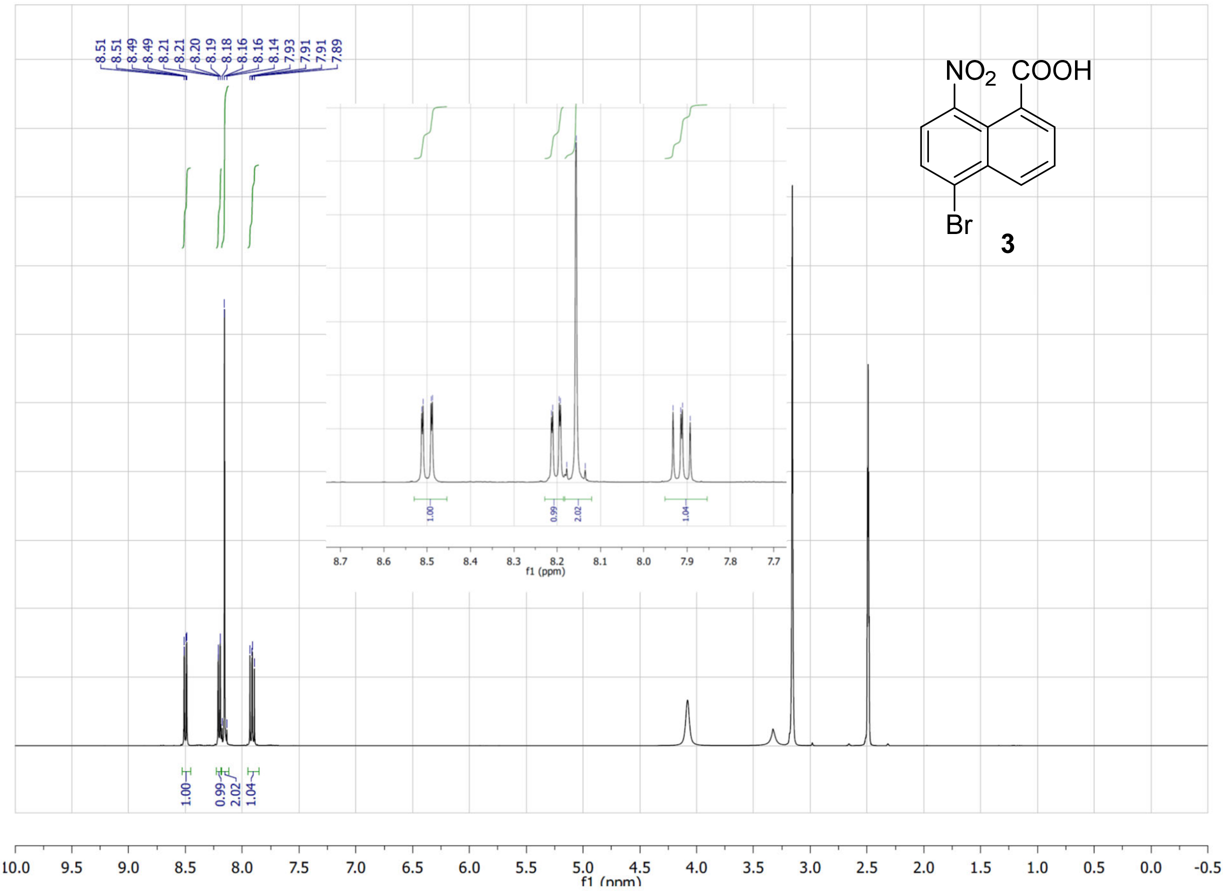

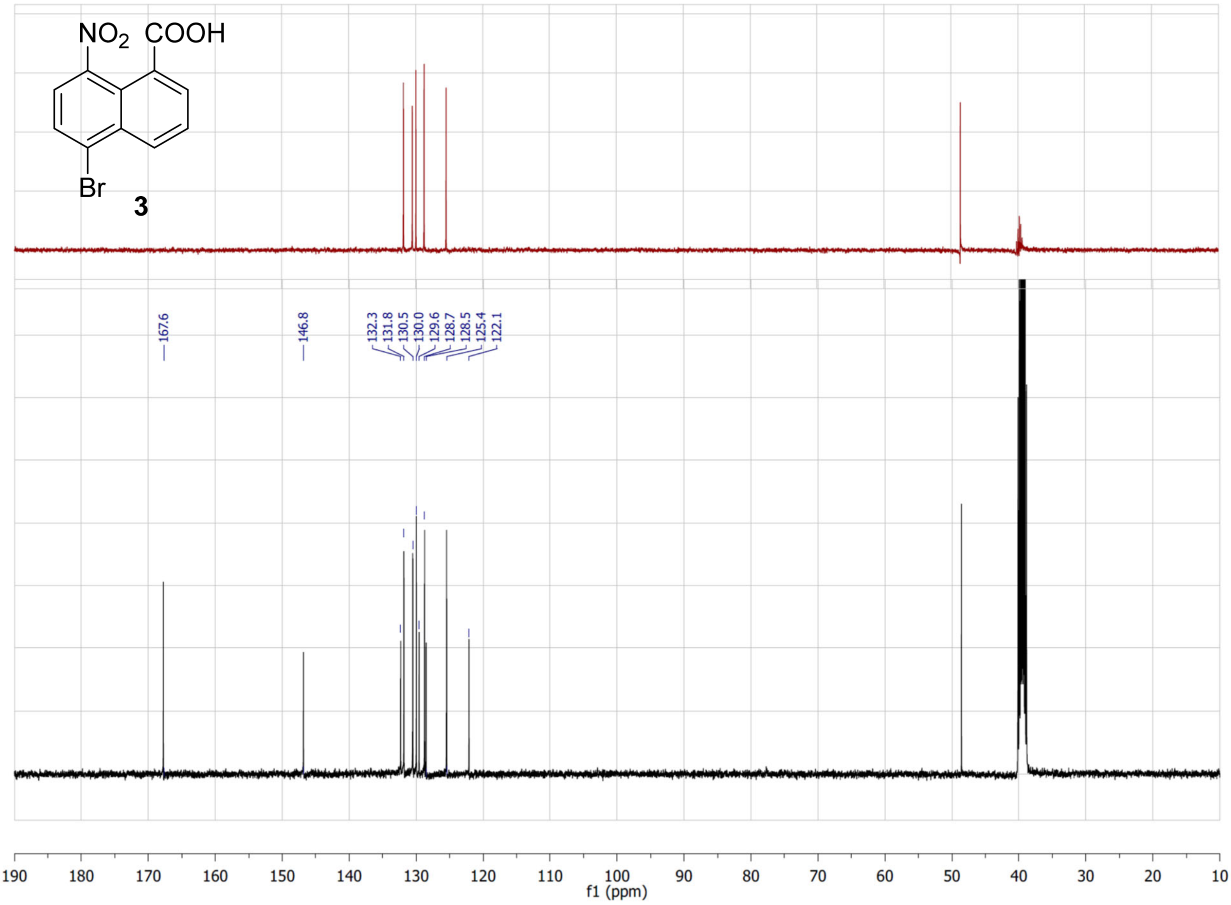

Figure S2. *^1H* NMR (*DMSO-d_6*, 400 MHz) *and ^{13}C* (*DMSO-d_6*, 100 MHz) *spectra of* (**3**).

Signals corresponding to traces of MeOH are visible.

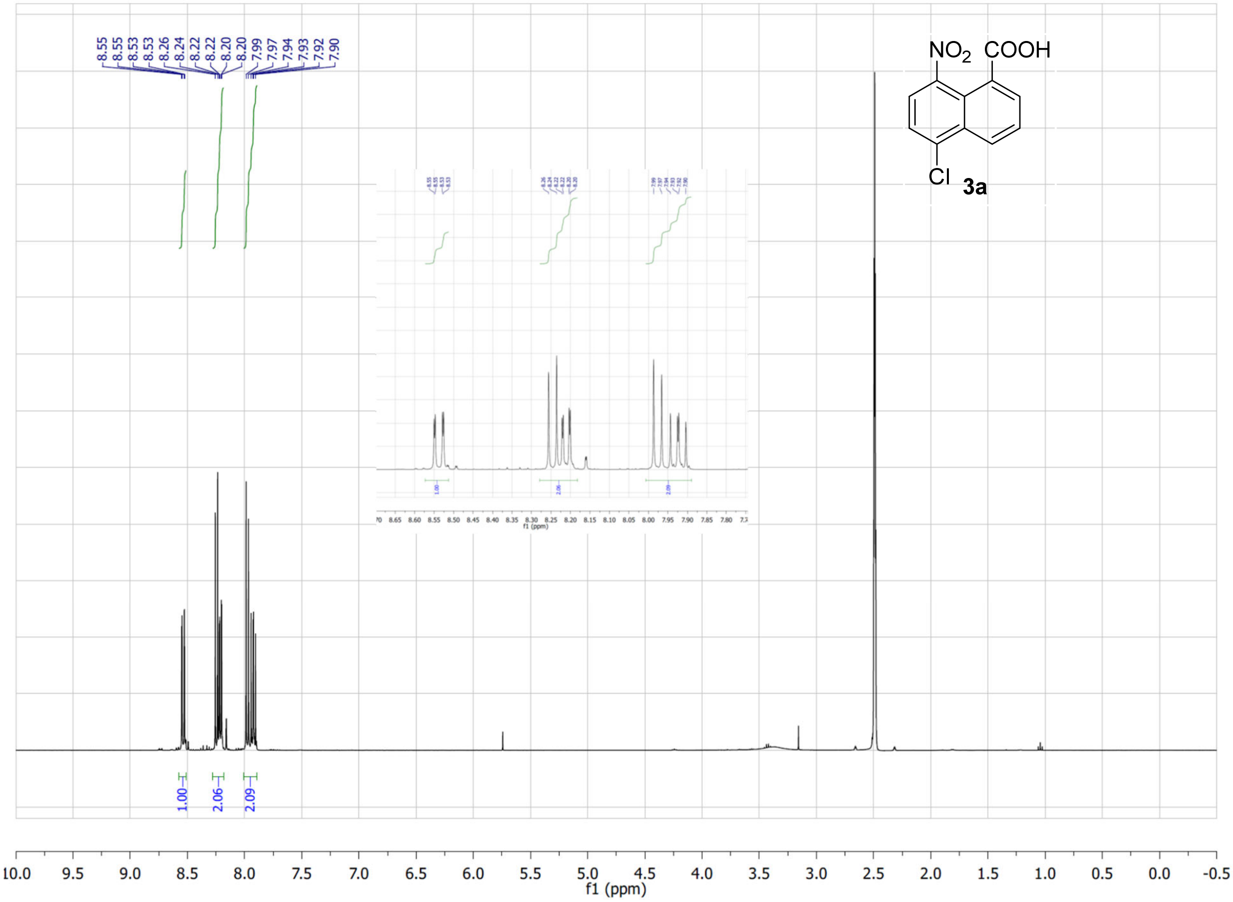

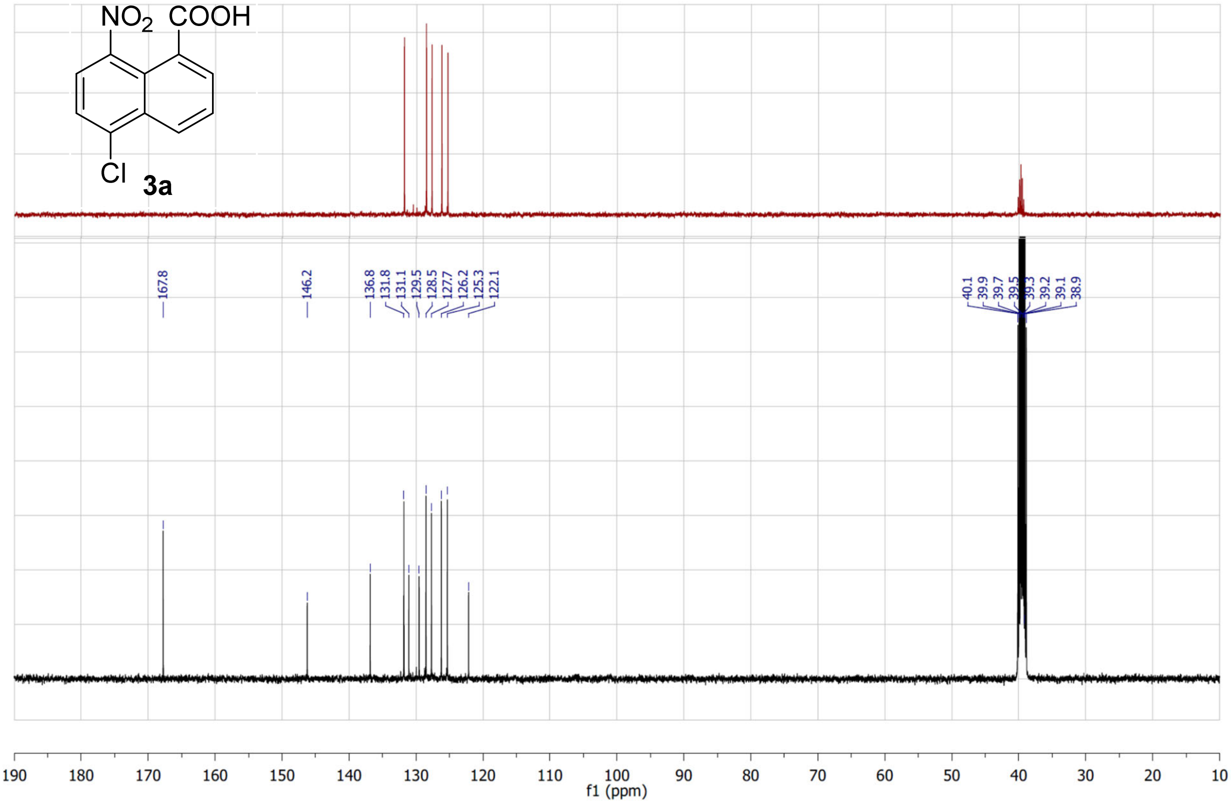

Figure S3. *^{1}H* NMR (*DMSO-d_6*, 400 MHz) *and ^{13}C* (*DMSO-d_6*, 100 MHz) *spectra of* ***(3a)***.

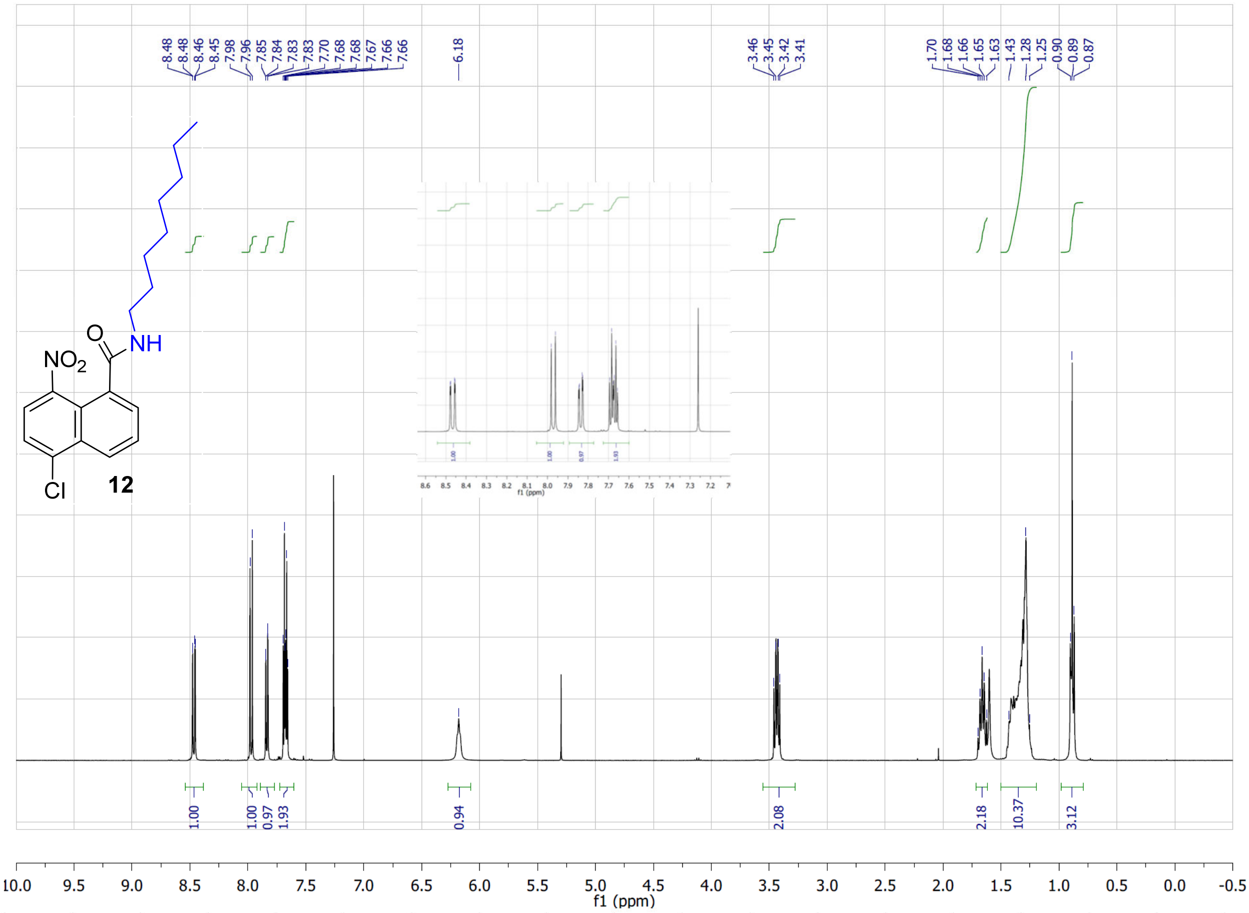

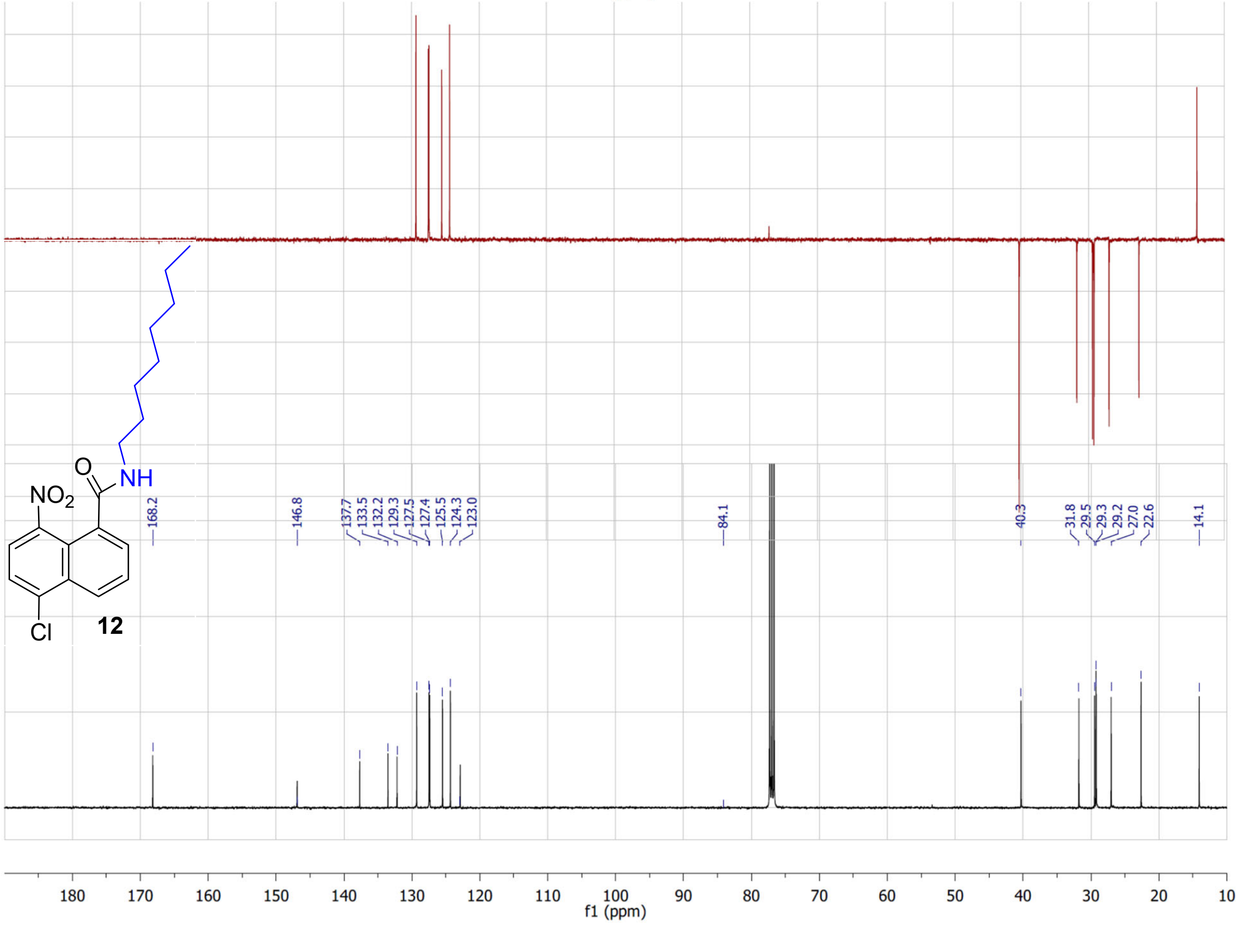

Figure S4. *¹H* NMR (*CDCl₃*, 400 MHz) *and ¹³C* (*CDCl₃*, 100 MHz) *spectra of* (***12***).

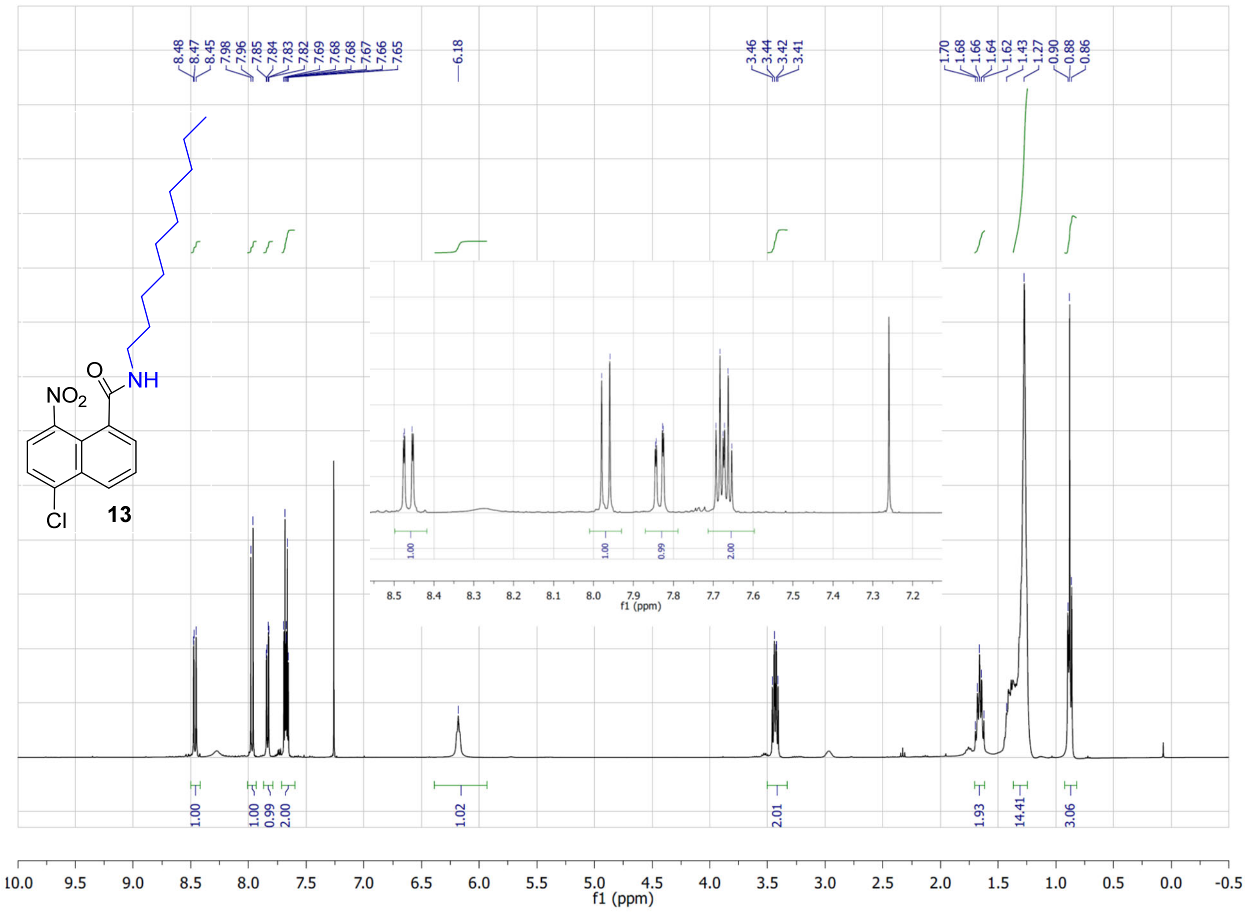

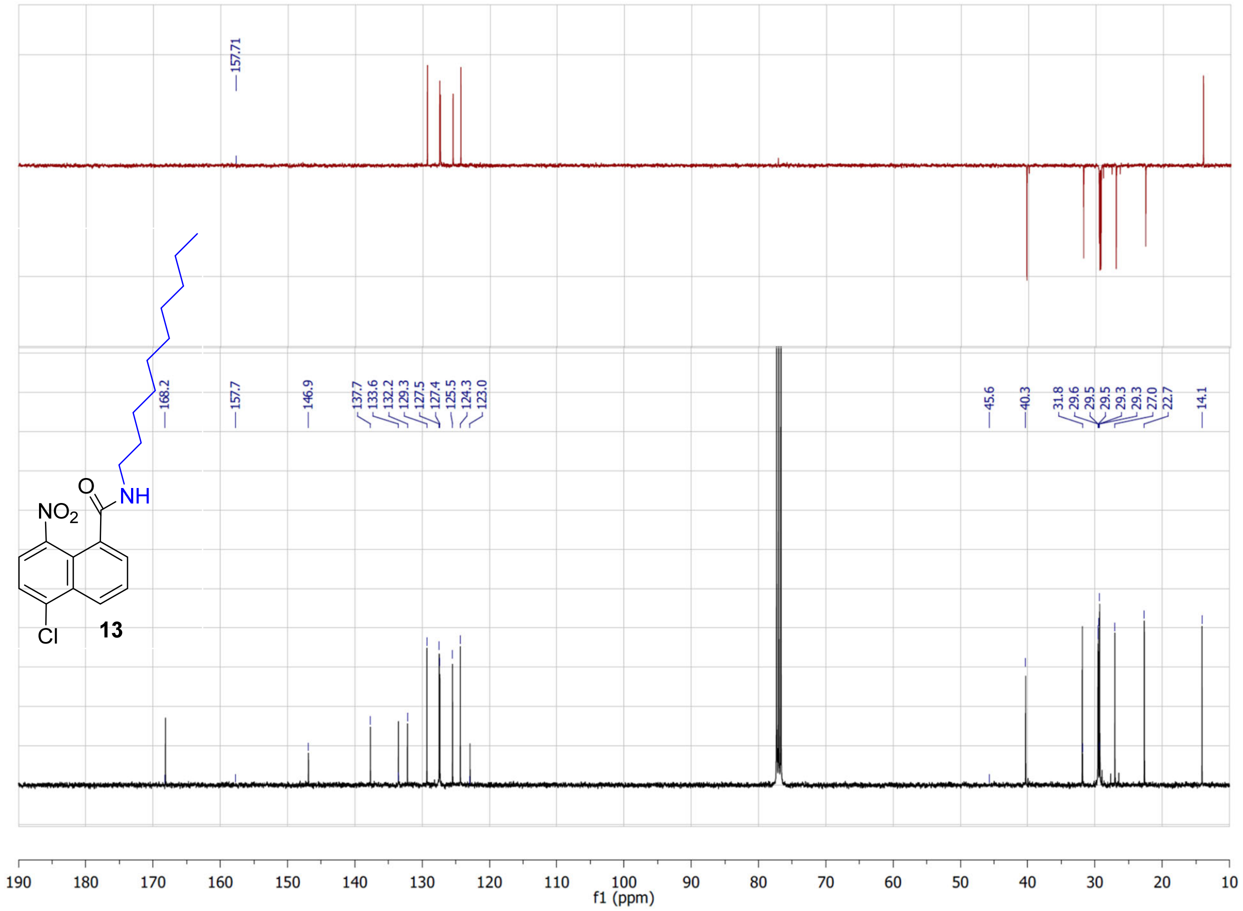

Figure S5. *^{1}H* NMR (*$CDCl_3$*, 400 MHz) *and ^{13}C* (*$CDCl_3$*, 100 MHz) *spectra of* (***13***).

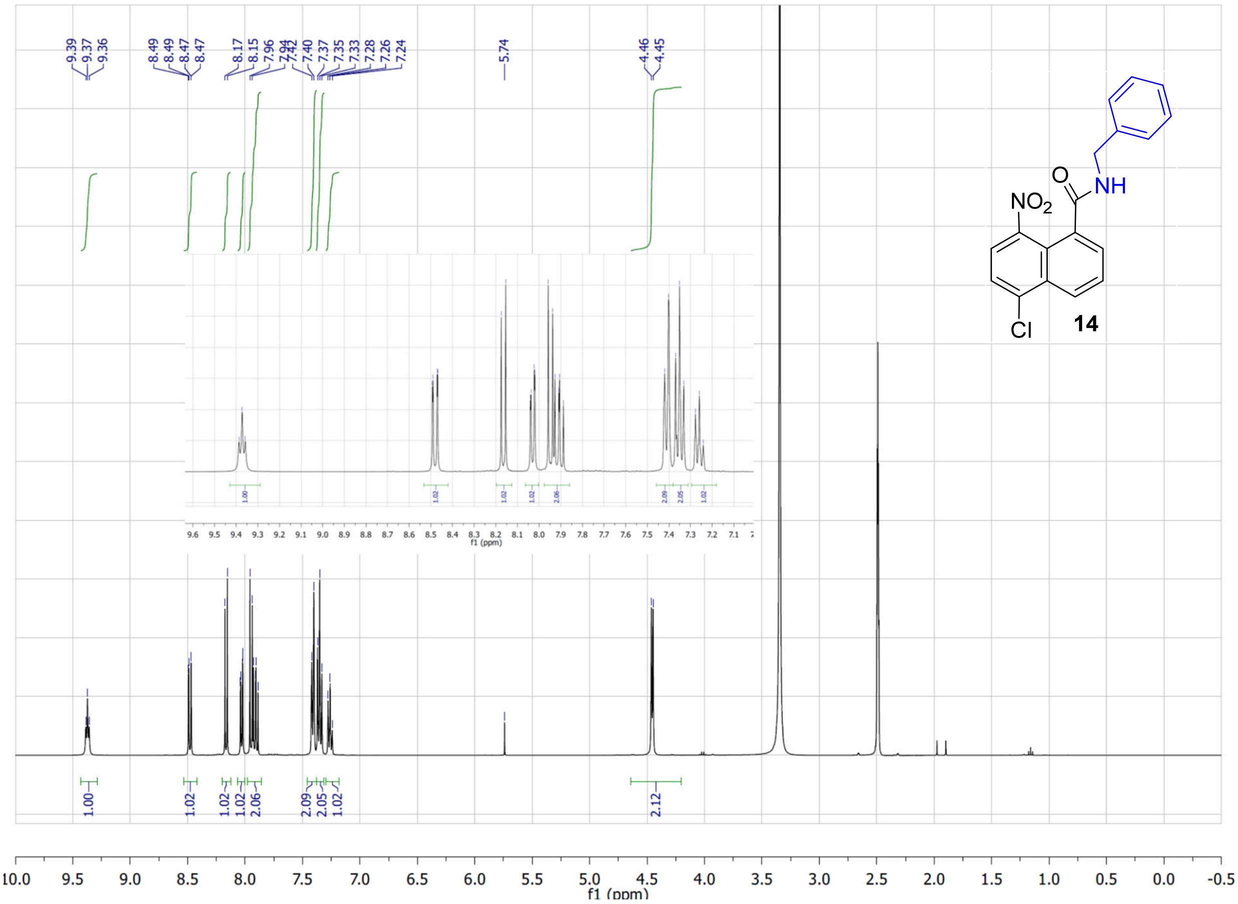

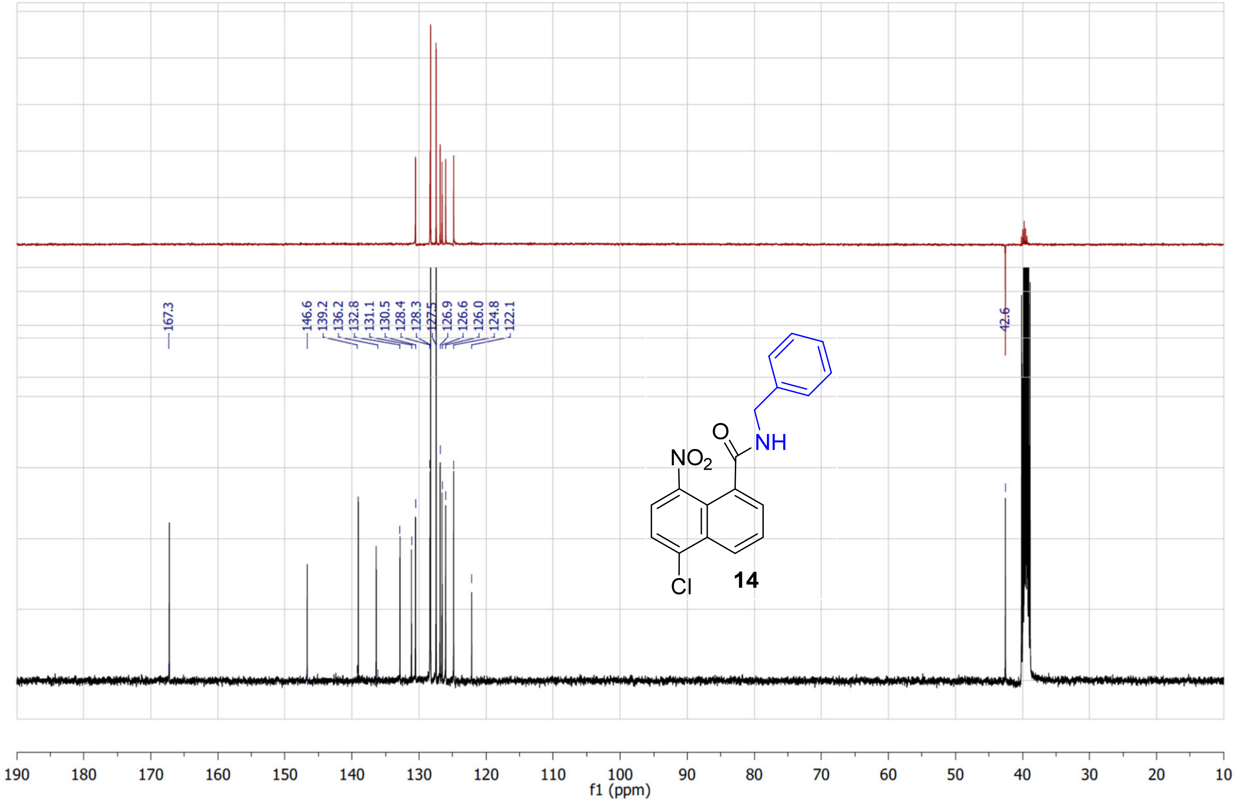

Figure S6. *^{1}H* NMR (*DMSO-d$_6$*, 400 MHz) *and ^{13}C* (*DMSO-d$_6$*, 100 MHz) *spectra of* (***14***).

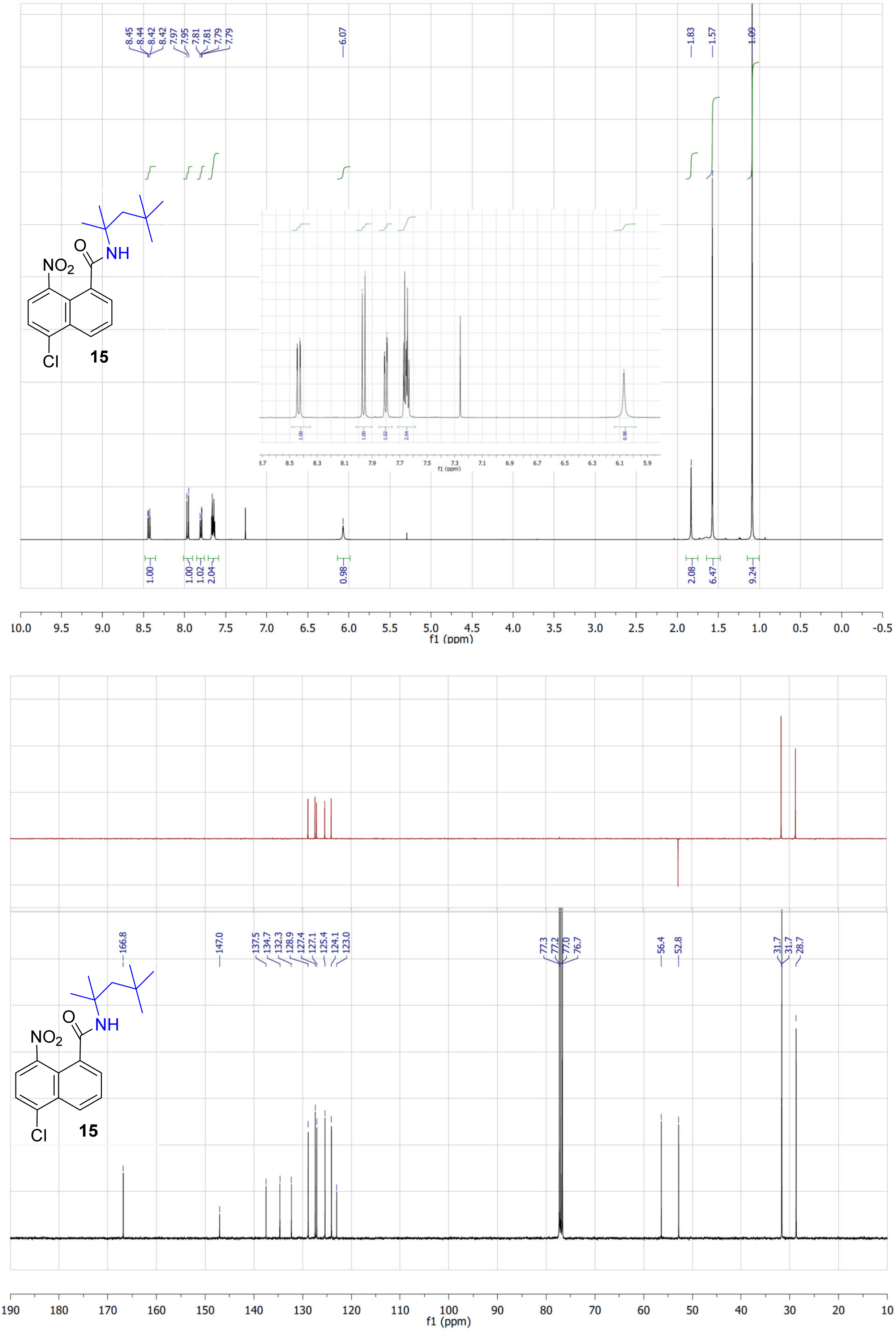

Figure S7. *^1H* NMR (*$CDCl_3$*, 400 MHz) *and* *^{13}C* (*$CDCl_3$*, 100 MHz) *spectra of* (***15***).

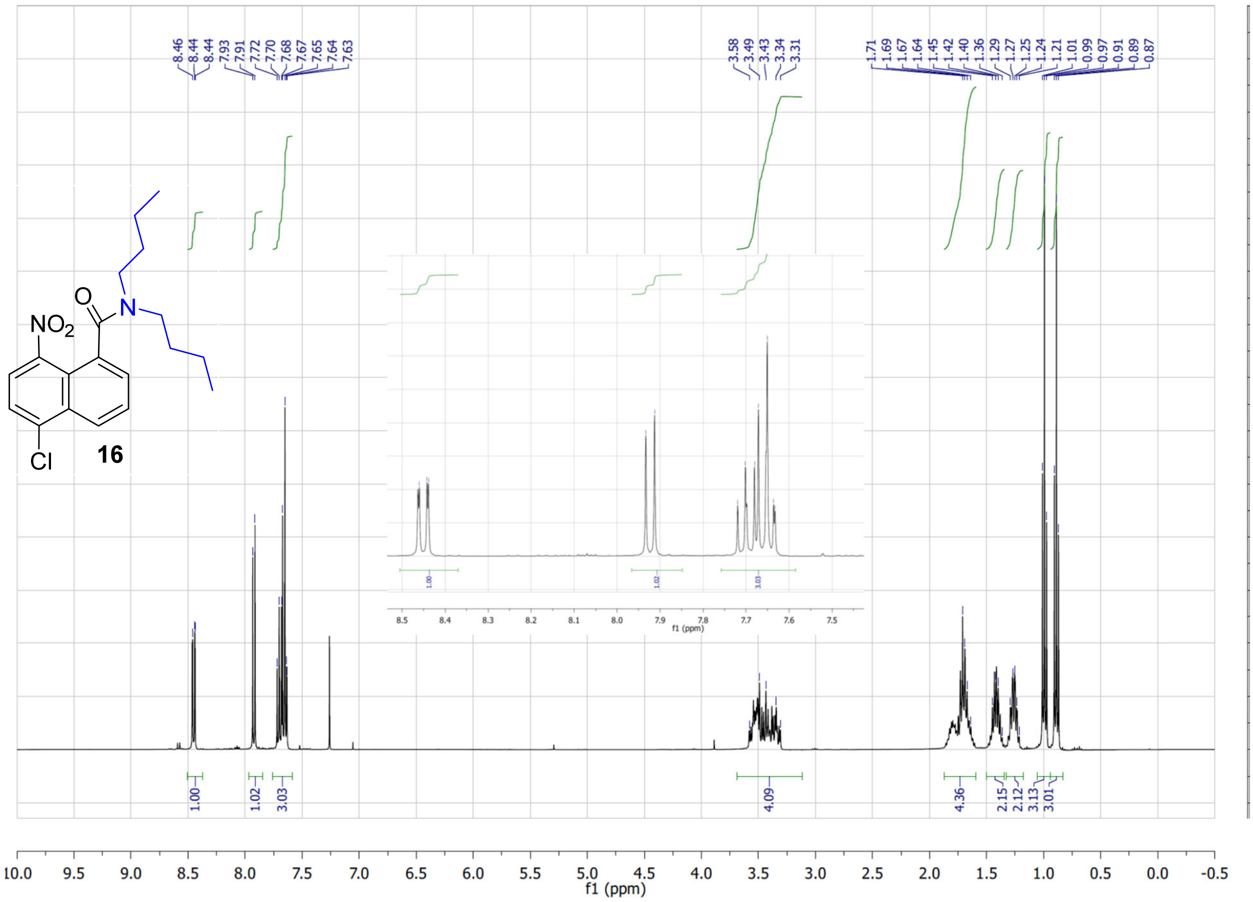

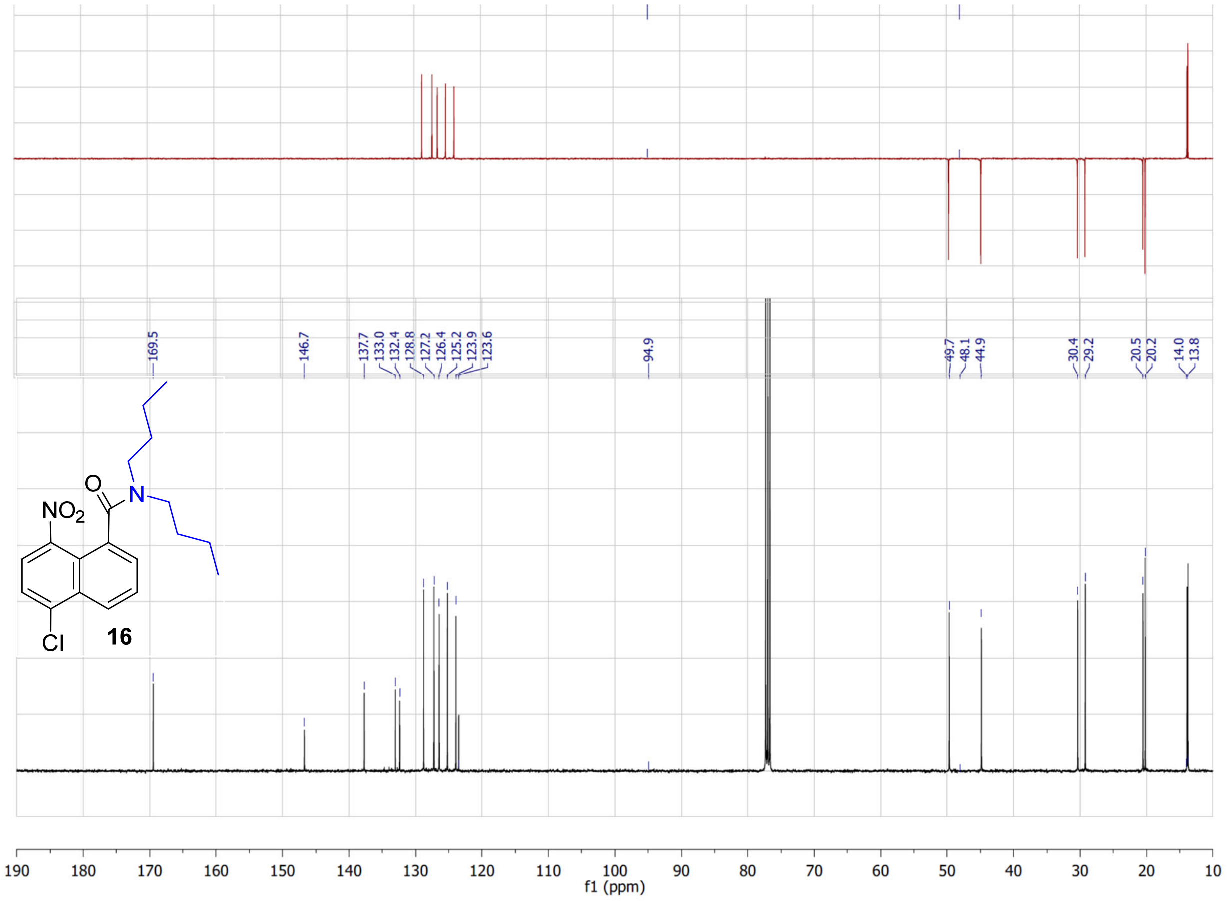

Figure S8. *^{1}H* NMR (*$CDCl_3$*, 400 MHz) *and ^{13}C* (*$CDCl_3$*, 100 MHz) *spectra of* (***16***).

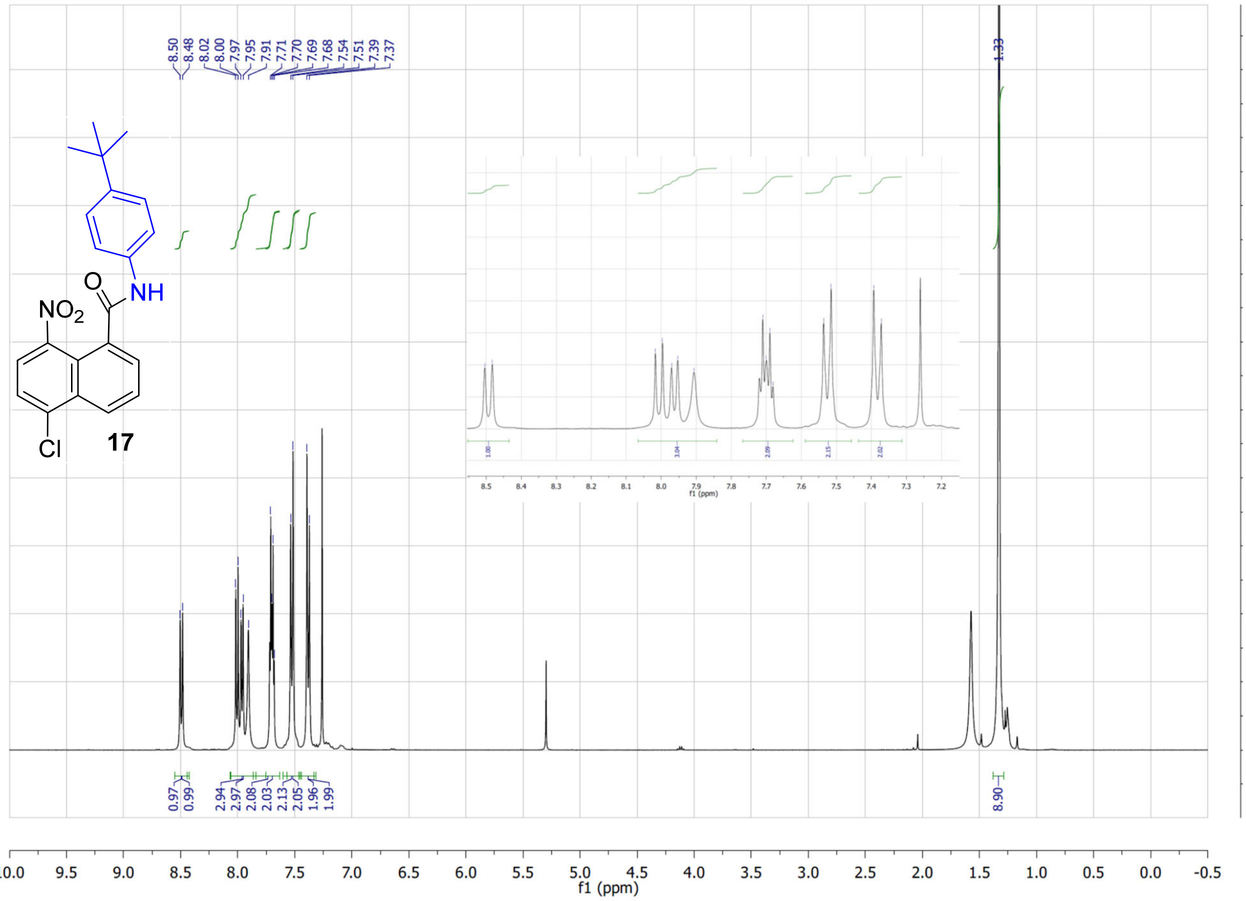

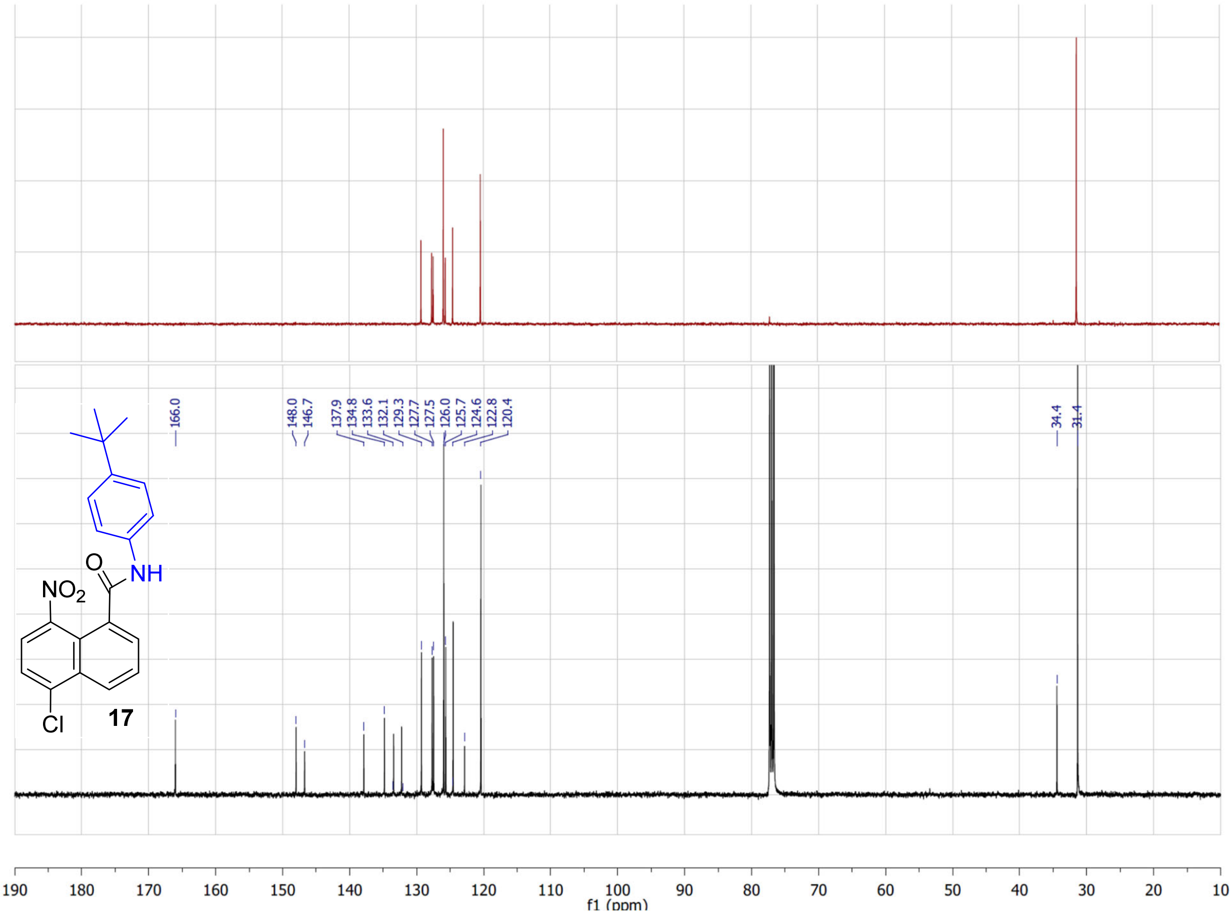

Figure S9. *^{1}H* NMR (*CDCl$_3$*, 400 MHz) *and ^{13}C* (*CDCl$_3$*, 100 MHz) *spectra of* (**17**).

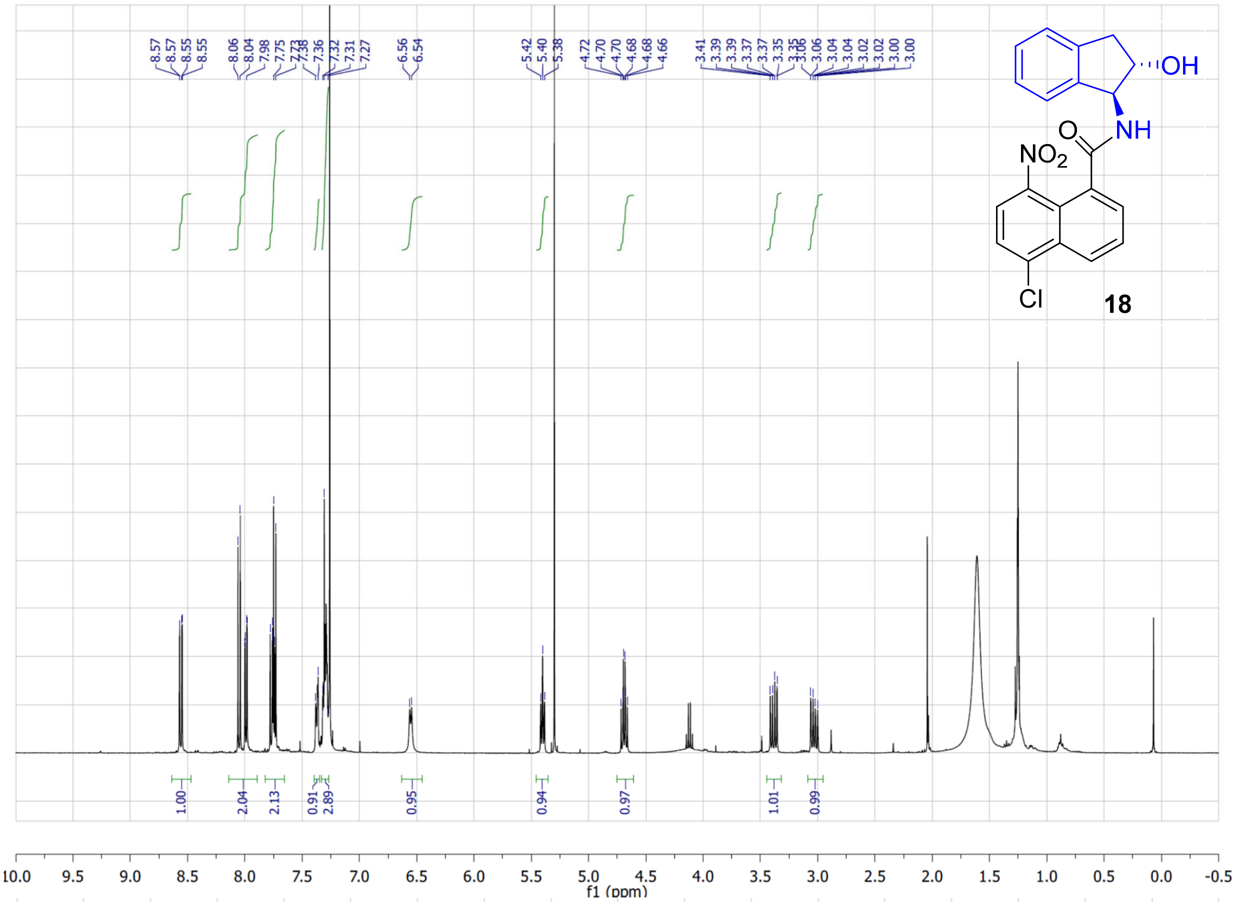

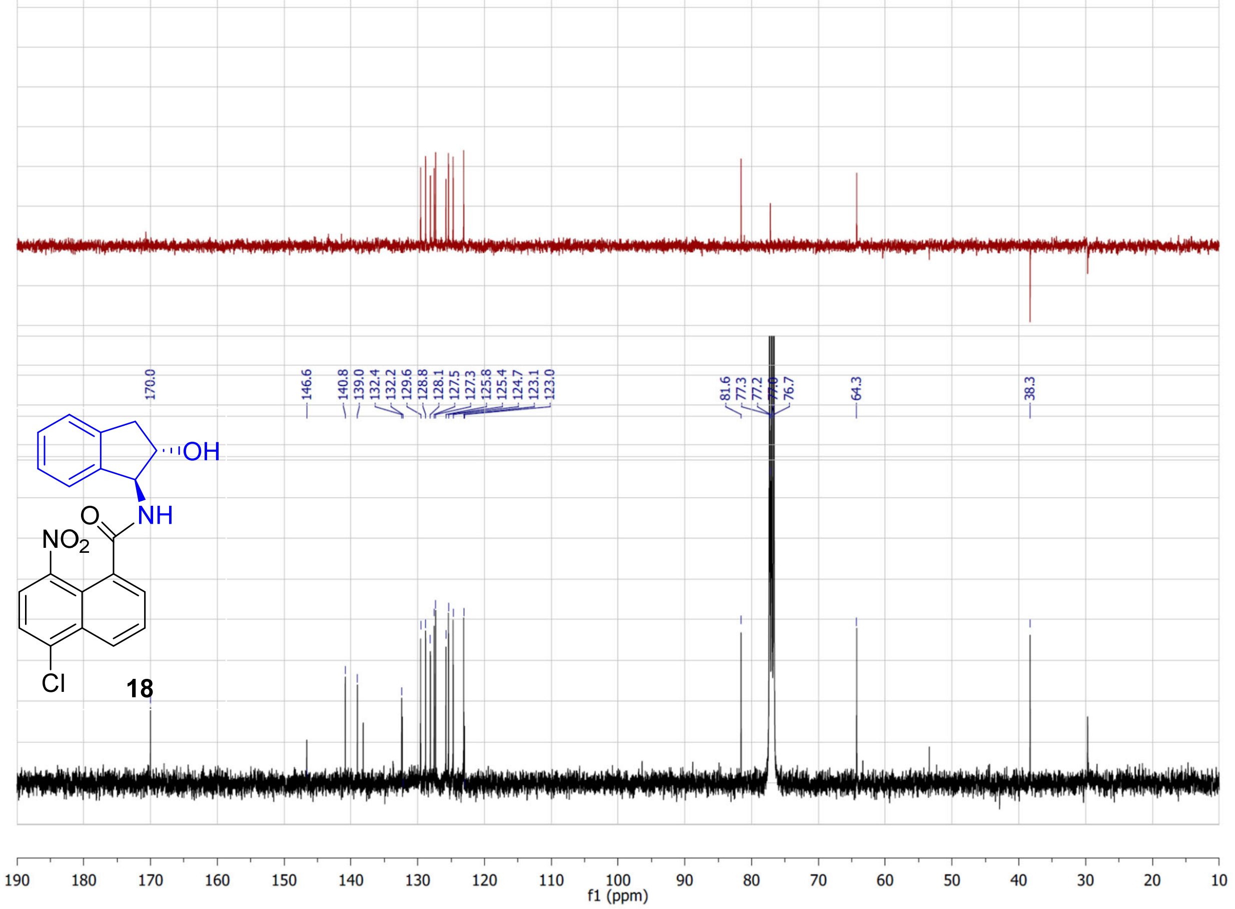

Figure S10. *¹H* NMR (*CDCl₃*, 400 MHz) *and* *¹³C* (*CDCl₃*, 100 MHz) *spectra of* (**18**).
Signals corresponding to traces of ethyl acetate are visible.

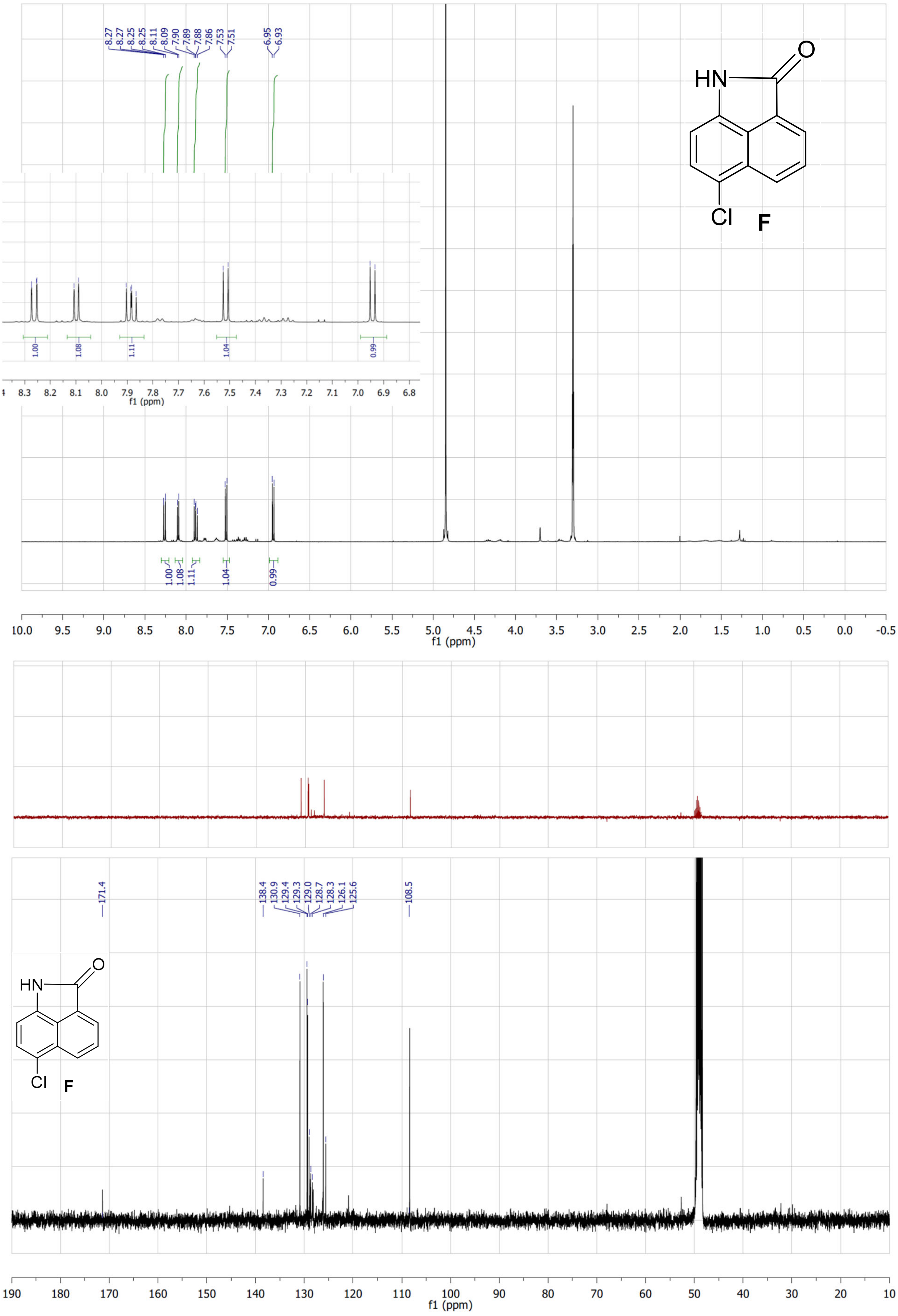

Figure S11. *^{1}H* NMR (*CD_3OD*, 400 MHz) *and ^{13}C* (*CD_3OD*, 100 MHz) *spectra of* (***F***).

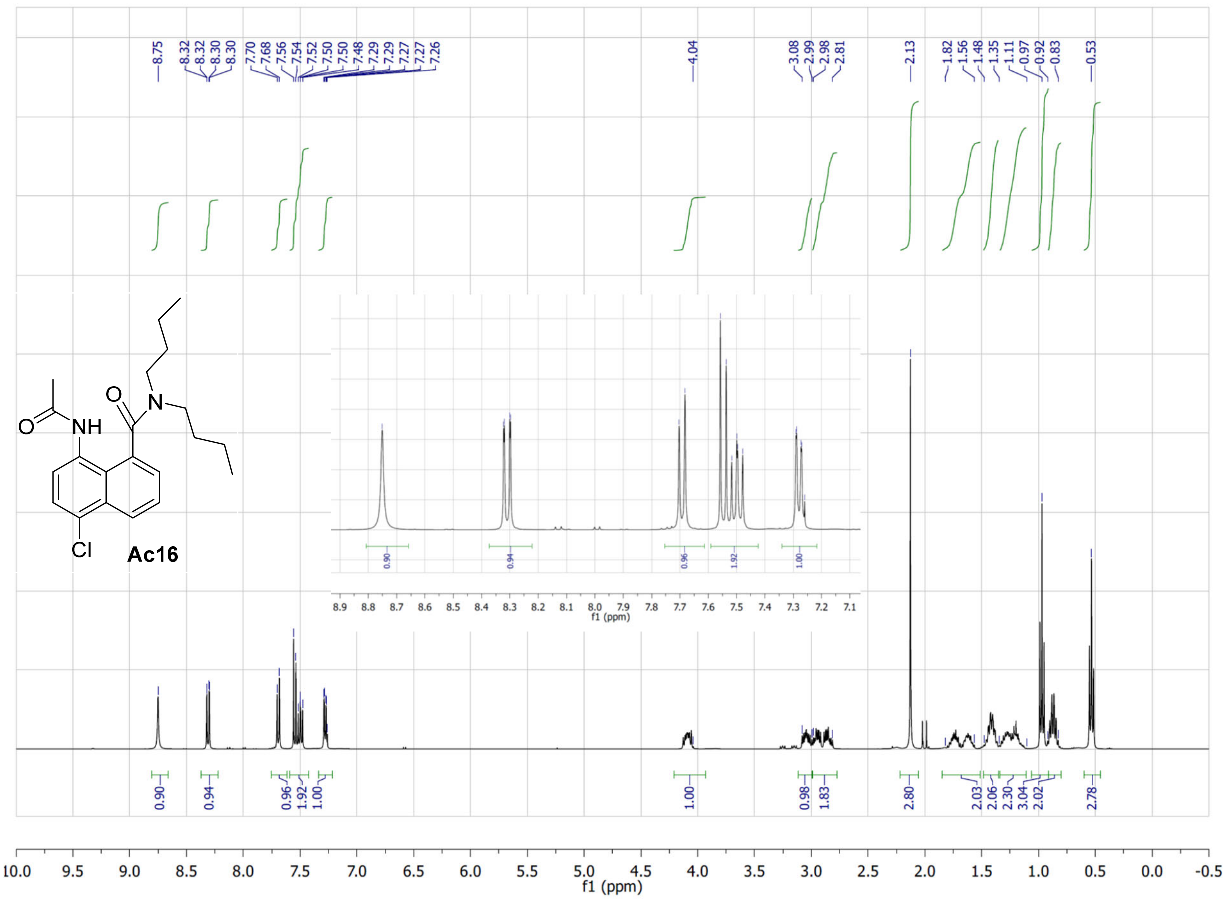

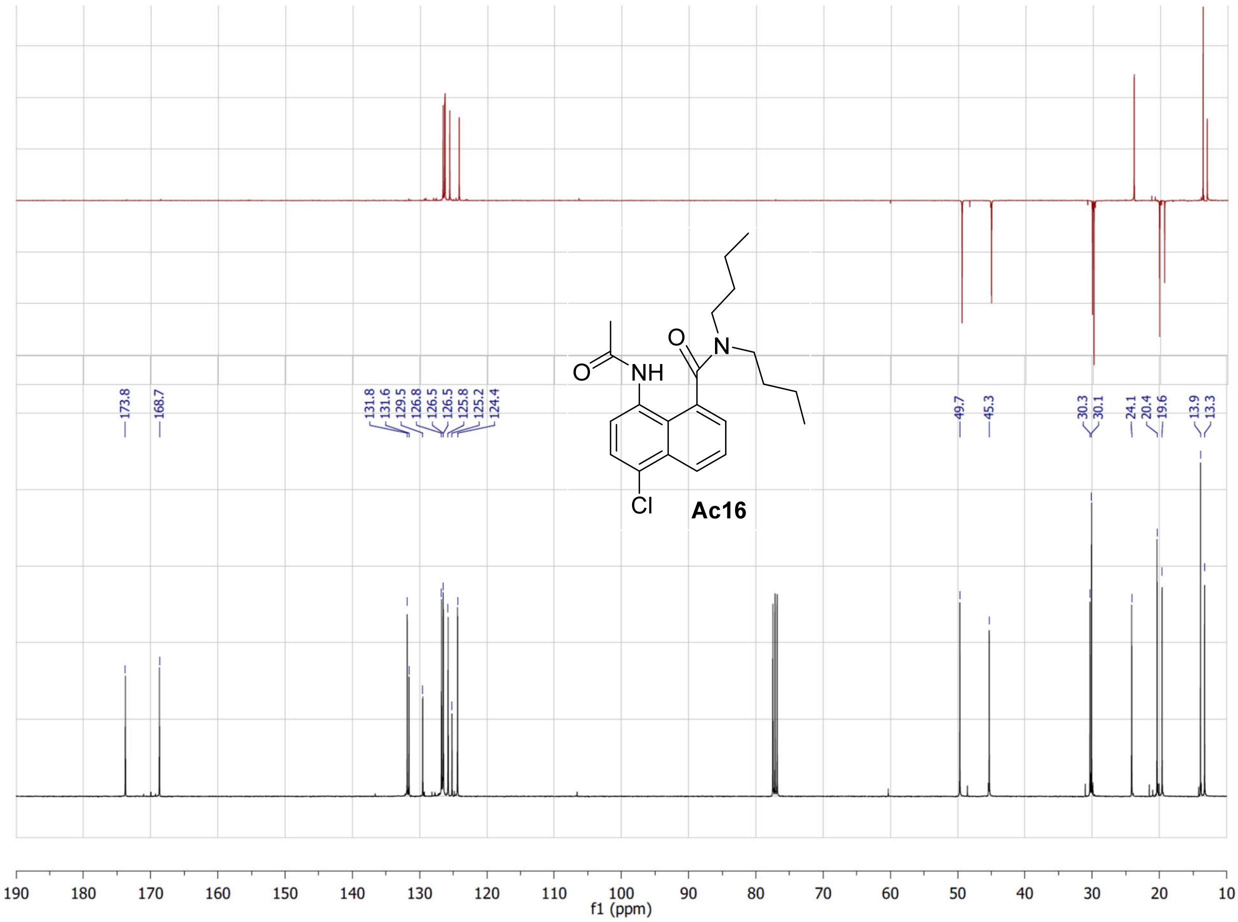

Figure S12. *^{1}H* NMR (*$CDCl_3$*, 400 MHz) *and ^{13}C* (*$CDCl_3$*, 100 MHz) *spectra of* (***Ac16***).

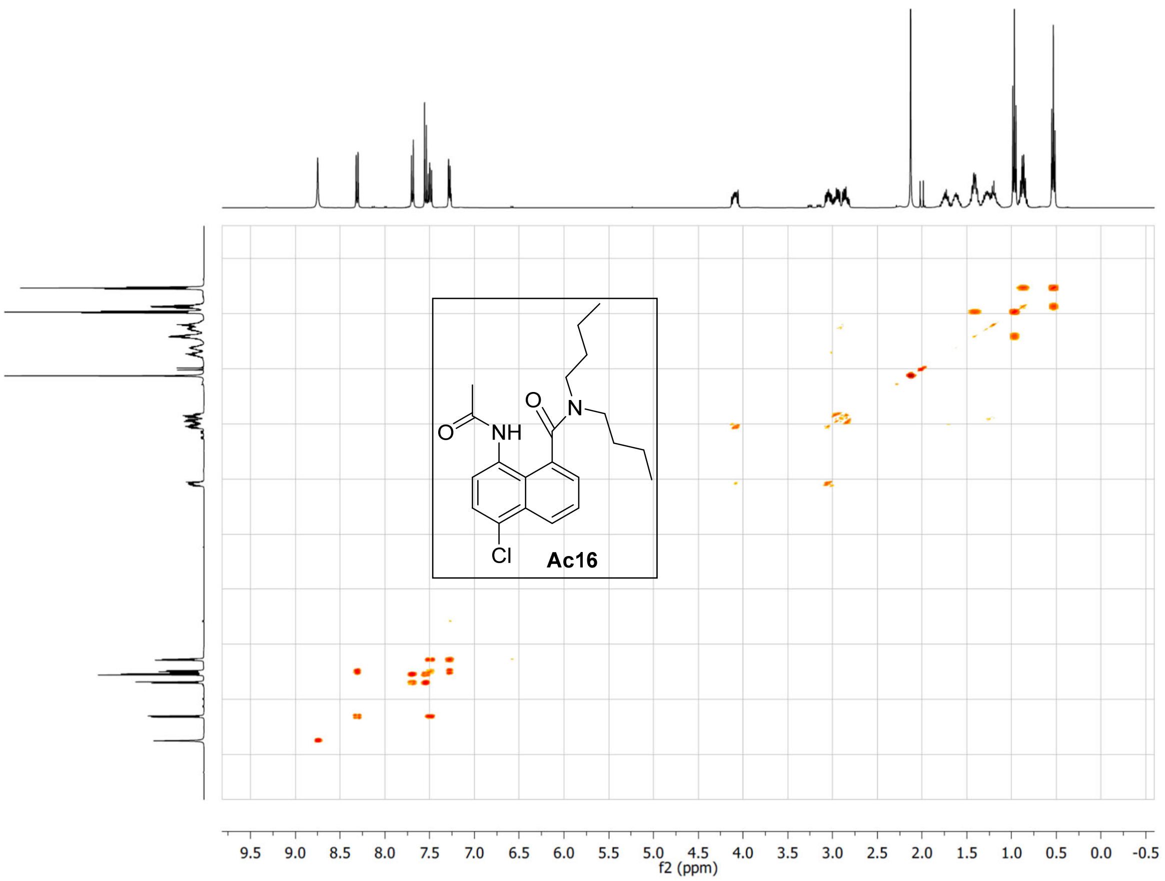

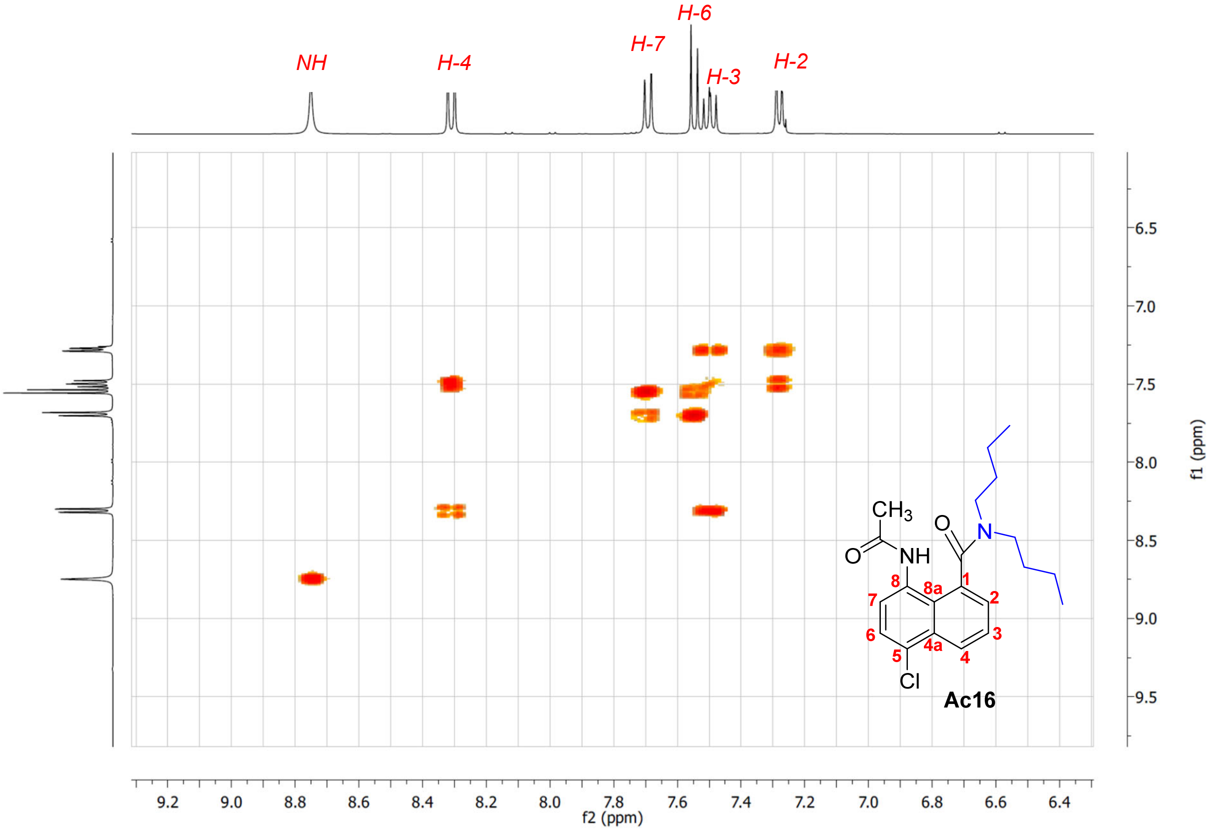

Figure S13. *COSY* (*CDCl*$_3$, 400 MHz) *spectrum (and expansion of the aromatic region) of (**Ac16**).*

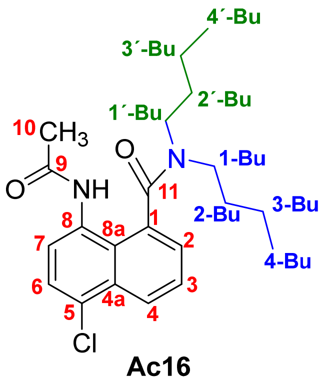

Ac16

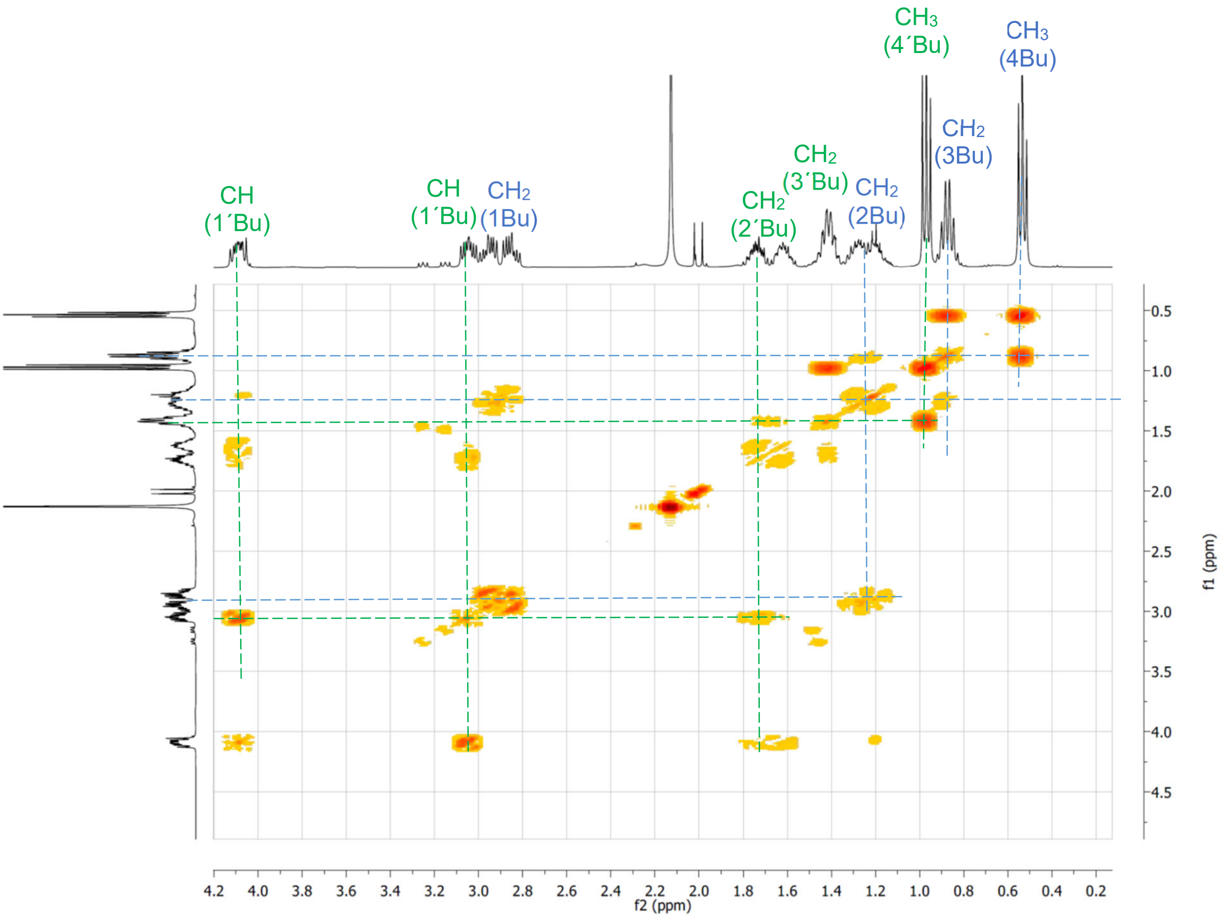

Figure S14. *COSY* (*CDCl3*, 400 MHz) *spectrum (and expansion 0 - 4.2 ppm) of* (***Ac16***).

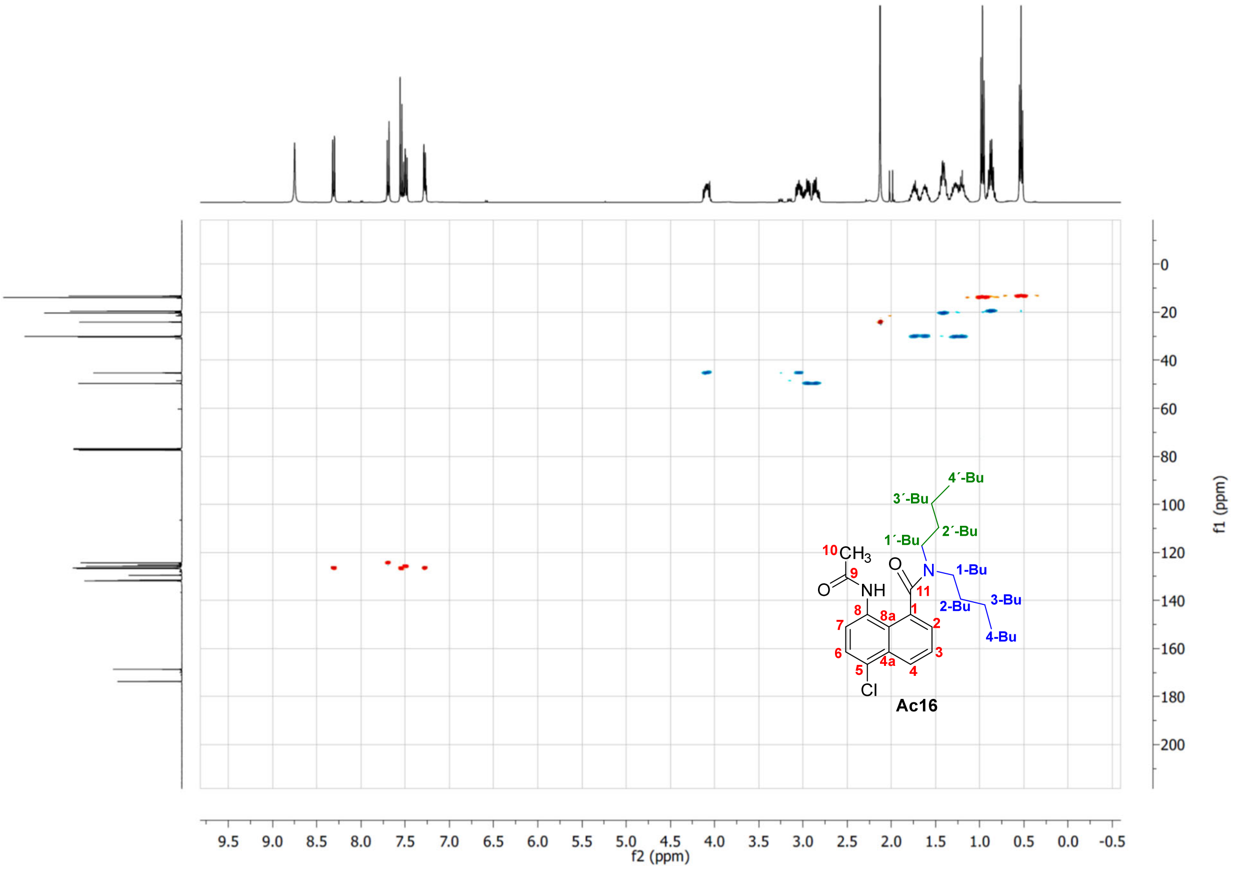

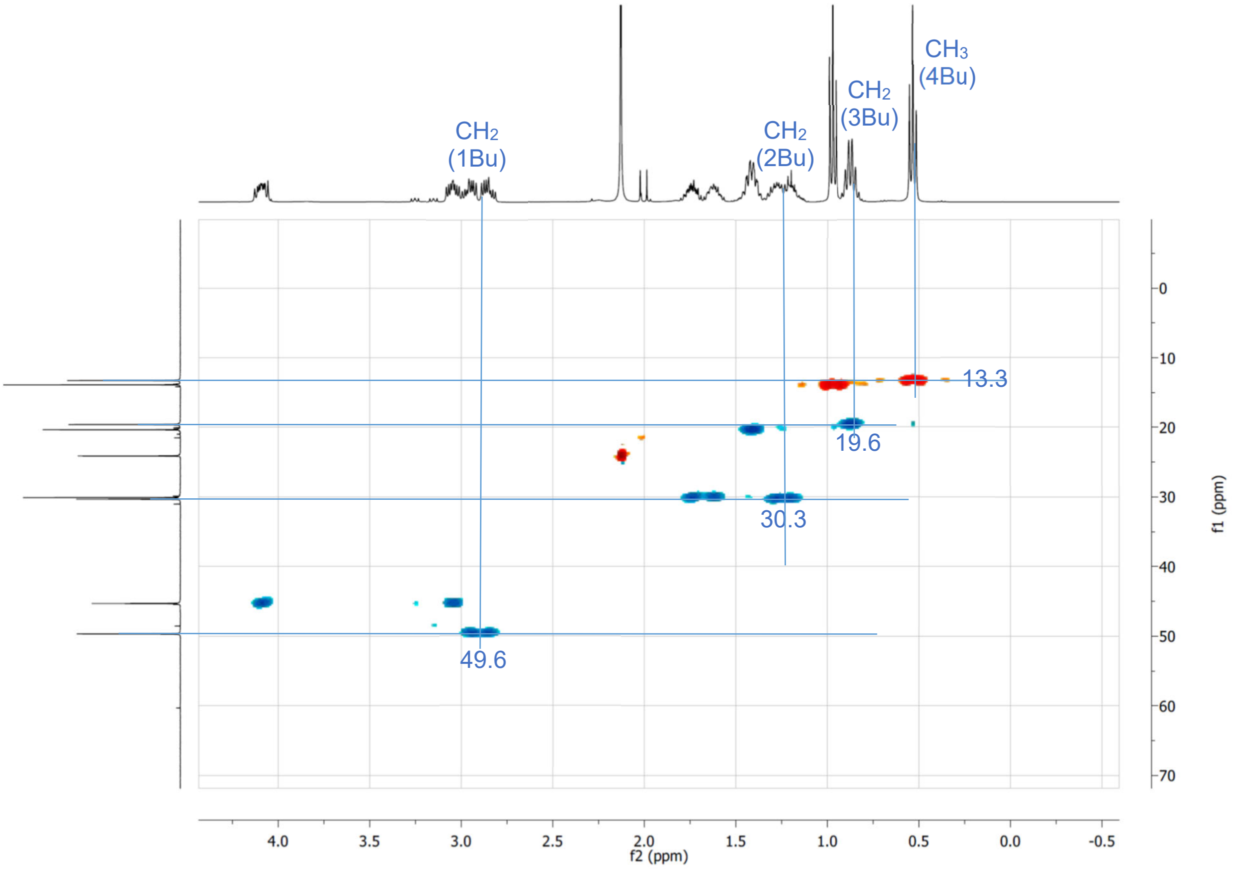

Figure S15. *HSQC 145 Hz (and expansion) of (**Ac16**) in CDCl$_3$.Spectra were recorded using a 400 MHz Bruker Avance NEO.*

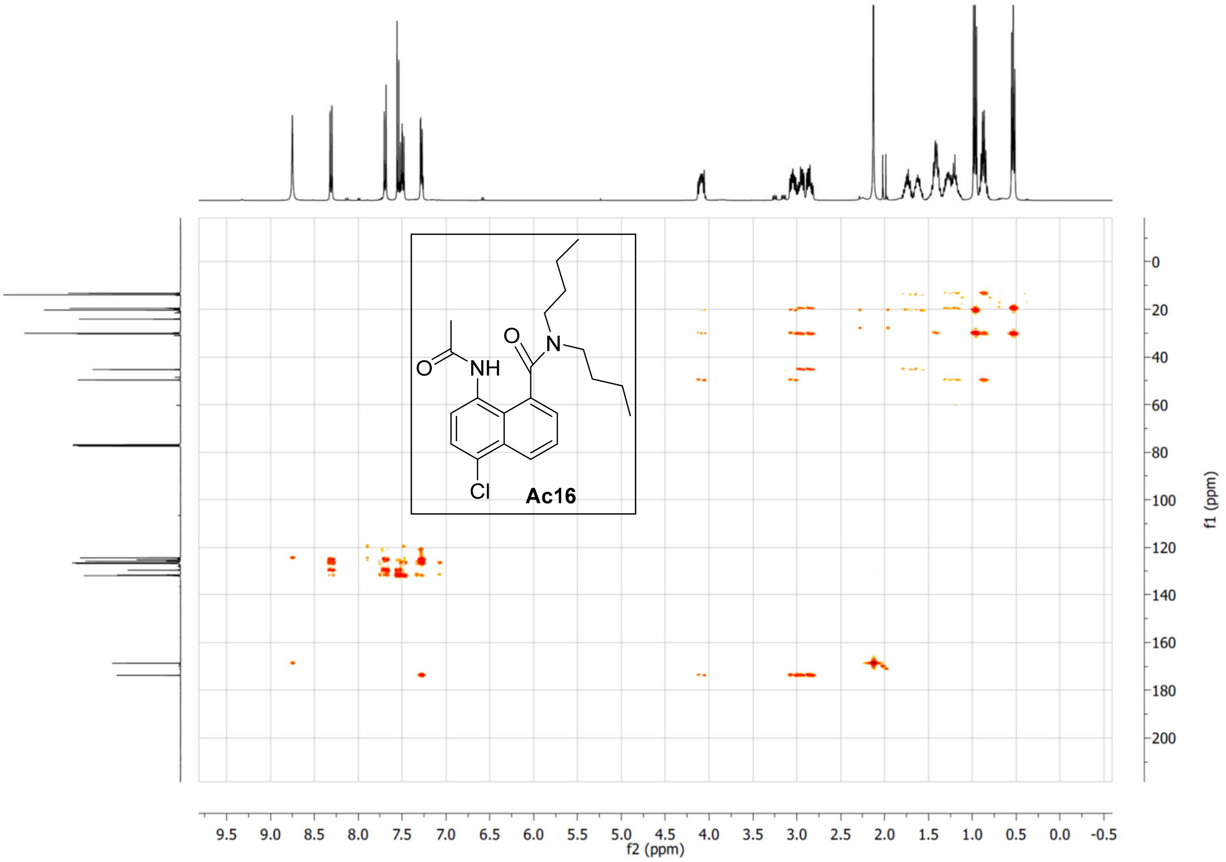

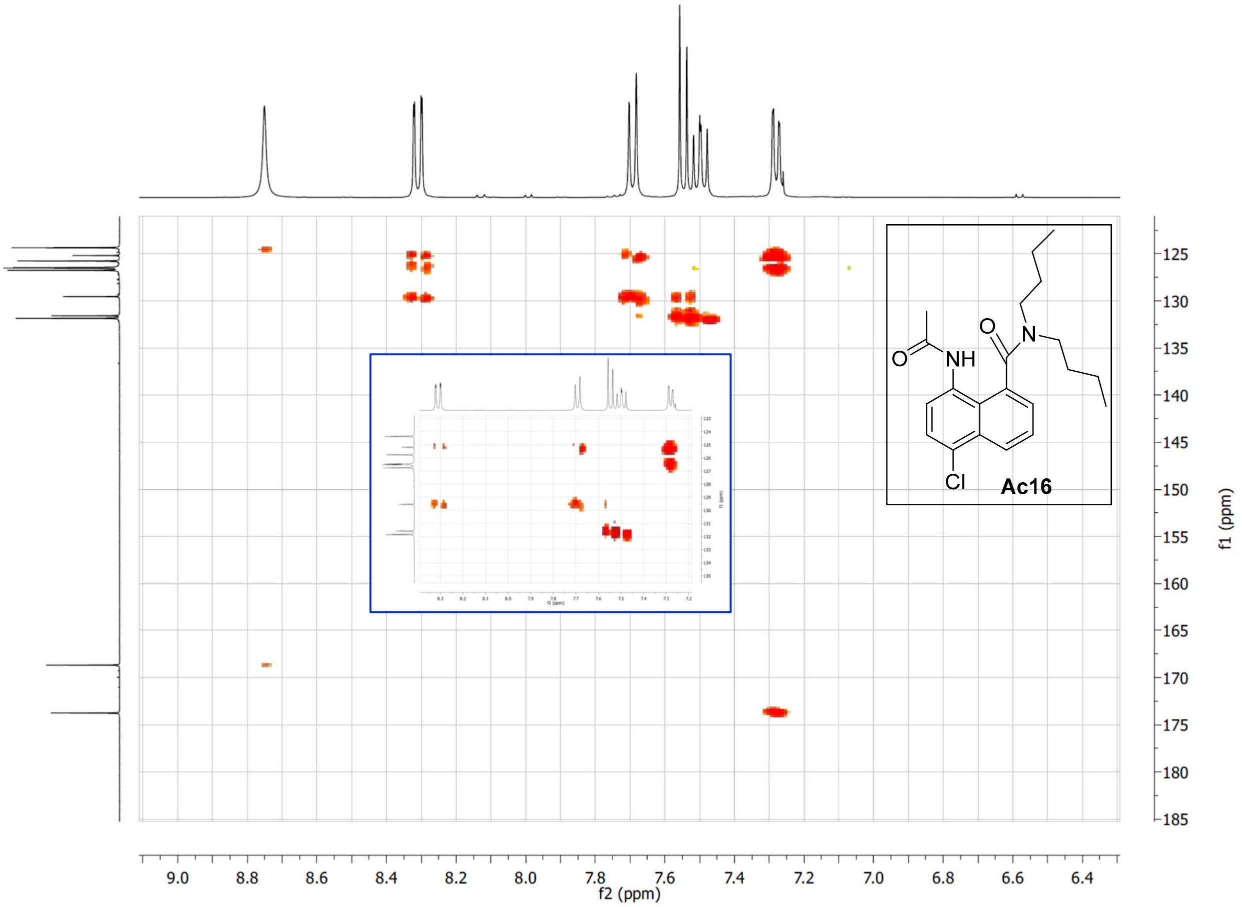

Figure S16. *HMBC CIGAR (and expansion) of (**Ac16**) in $CDCl_3$. Spectra were recorded using a 400 MHz Bruker Avance NEO.*

Table S1. *^{1}H* NMR (*$CDCl_3$*, 400 MHz) *and ^{13}C* (*$CDCl_3$*, 100 MHz) *chemical shift assignments for* ***Ac16***

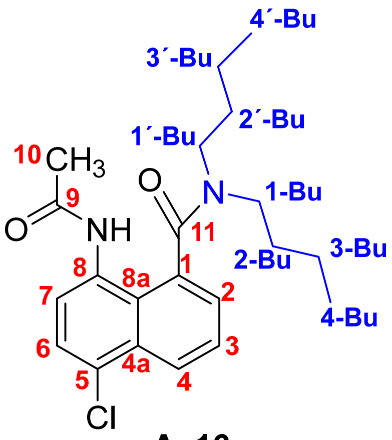

Ac16

	^{1}H NMR (δ)		^{13}C NMR (δ)
NH	8.75 (s, 1H)	C-9	168.7 (C)
H-4	8.31 (dd, 1H, J = 8.4 and 1.2 Hz)	C-4	126.5 (CH)
H-7	7.69 (d, 1H, J = 8.0 Hz)	C-7	124.4 (CH)
H-6	7.55 (d, J = 8.0 Hz, 1H)	C-6	126.8 (CH)
H-3	7.50 (dd, 1H, J = 8.4 and 7.2 Hz),	C-3	125.8 (CH)
H-2	7.28 (dd, 1H, J = 7.2 and 1.2 Hz,)	C-2	126.5 (CH)
H-1´-Bu$_{(\alpha)}$	4.13 – 4.01 (m, 1H)	C-1´-Bu	45.3 (CH_2)
H-1´-Bu$_{(\beta)}$	3.08 – 2.99 (m, 1H)		
H-1- Bu	2.98 – 2.81 (m, 2H)	C-1-Bu	49.6 (CH_2)
H-CH_3	2.13 (s, 3H)	C-10	24.1(CH_3)
H-2´-Bu	1.82 – 1.56 (m, 2H)	C-2´-Bu	30.1(CH_2)
H-3´-Bu	1.48– 1.35 (m, 2H)	C-3´-Bu	20.4 (CH_2)
H-2-Bu	1.35 – 1.11 (m, 2H)	C-2-Bu	30.3 (CH_2)
H- 4´- Bu	0.97 (t, 3H, J = 7.2 Hz)	C-4´- Bu	13.9 (CH_3)
H-3-Bu	0.92 – 0.83 (m, 2H)	C-3-Bu	19.6 (CH_2)
H-4-Bu	0.53 (t, 3H J = 7.2 Hz).	C-4-Bu	13.3 (CH_3)
		C-11	173.8 (C)
		C-1	131.8 (C)
		C-4a	131.8 (C)
		C-5	129.5 (C)
		C-8	131.6 (C)
		C-8a	125.2 (C)

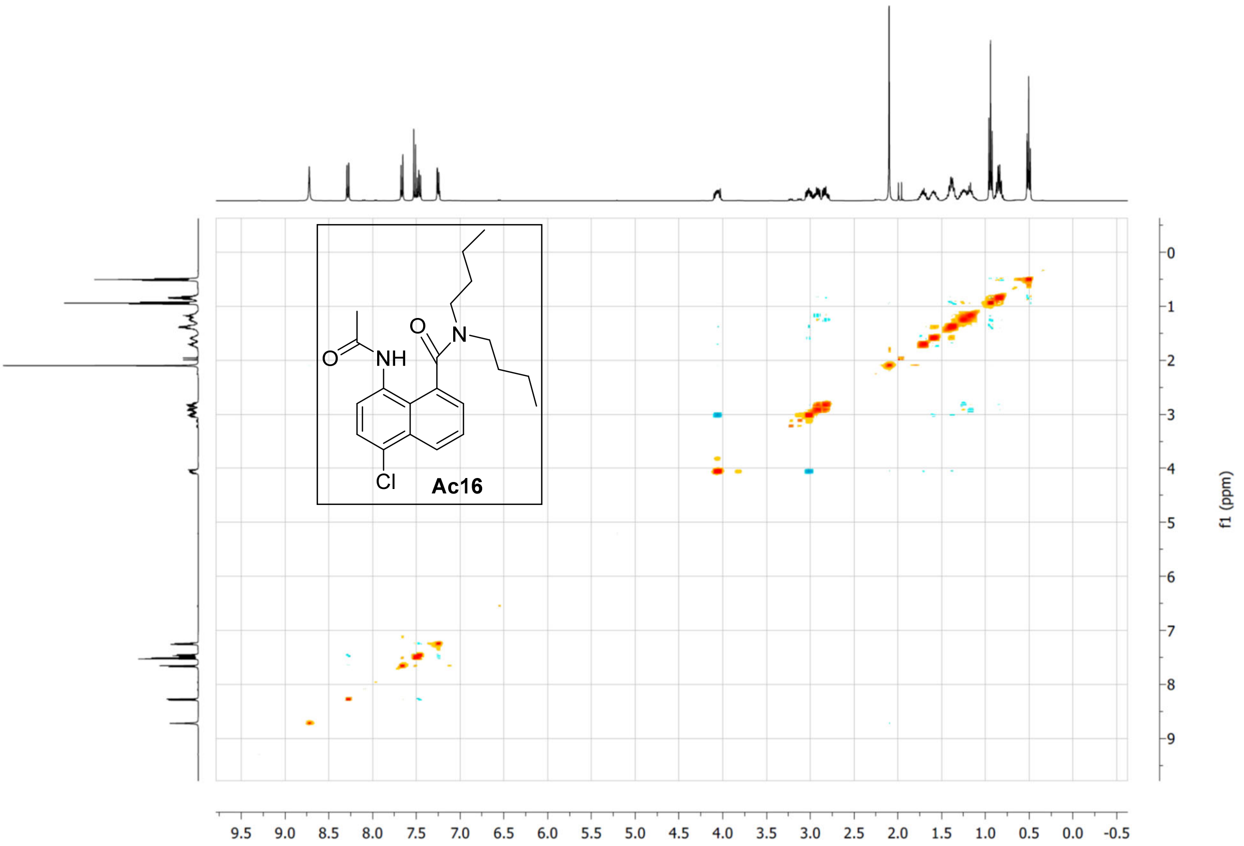

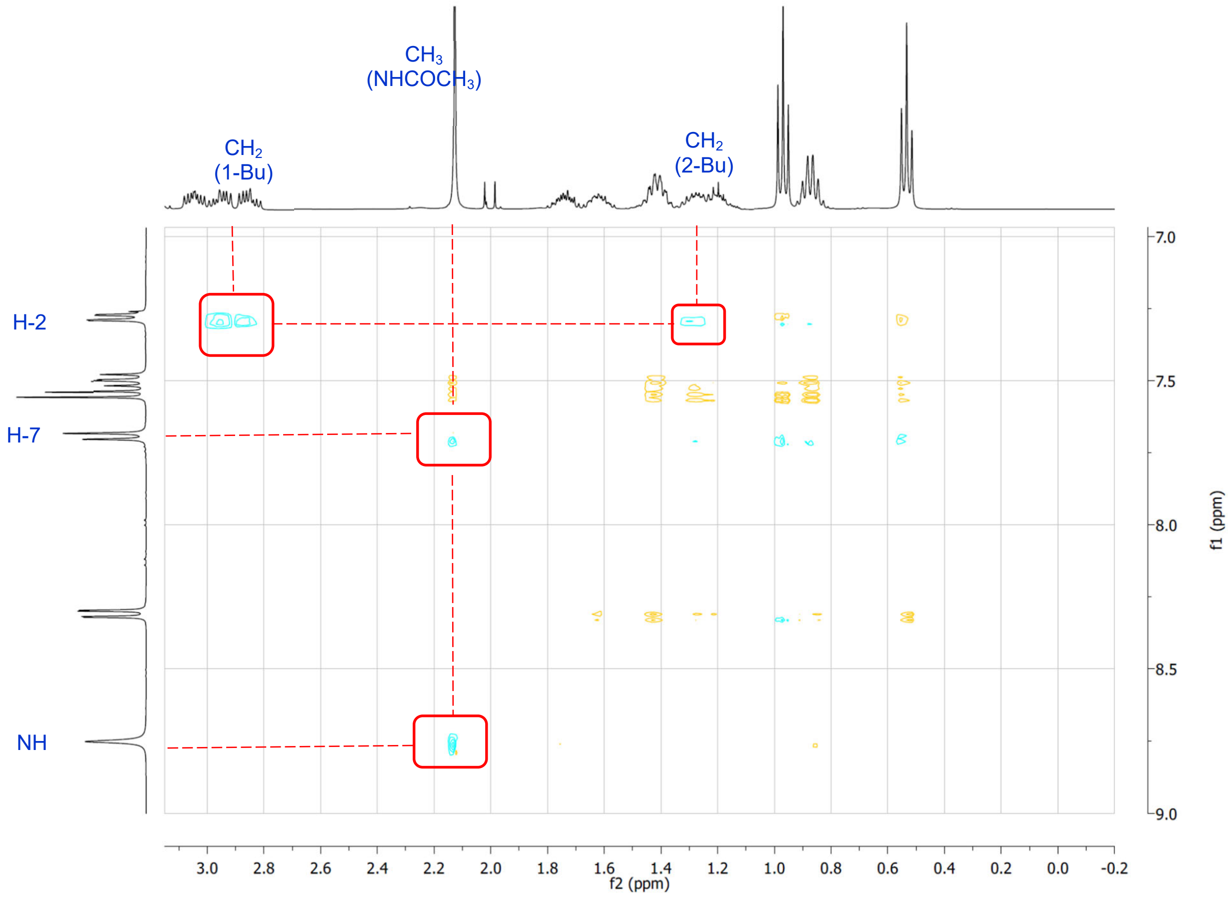

Figure S17. *ROESY (of (**Ac16**) in $CDCl_3$ showing the main correlations. Spectra were recorded using a 400 MHz Bruker Avance NEO.*

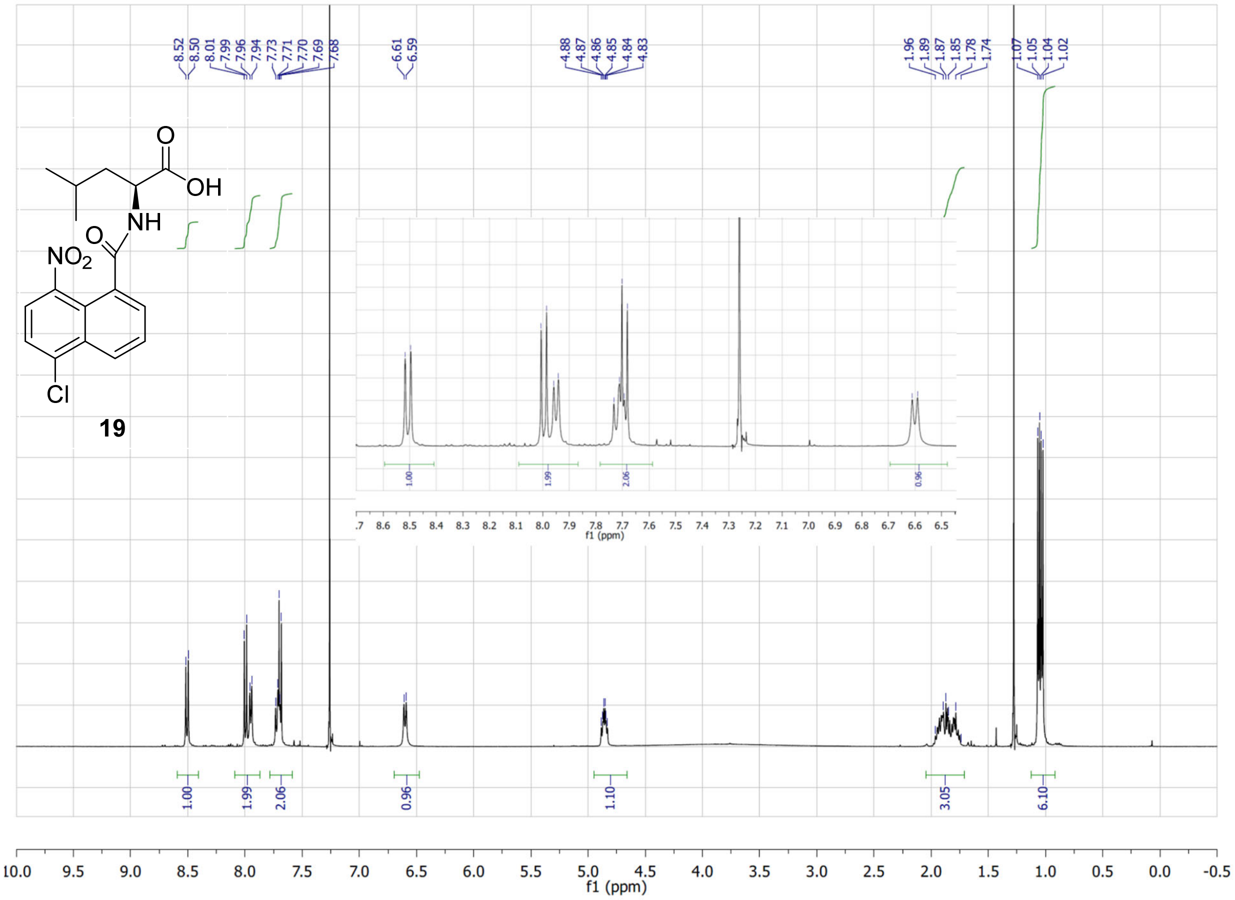

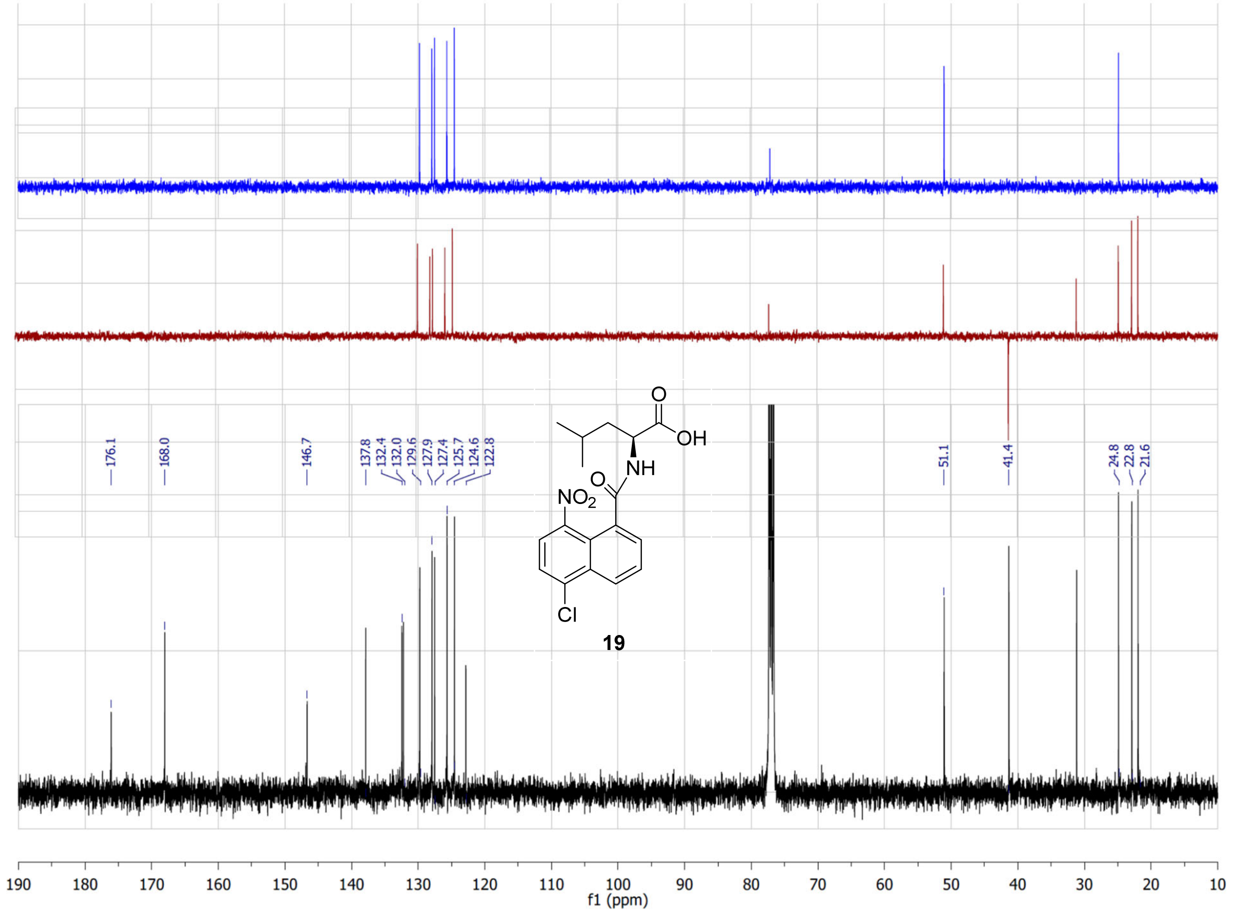

Figure S18. *¹H* NMR (*CDCl₃*, 400 MHz) *and* *¹³C* (*CDCl₃*, 100 MHz) *spectra of* (***19***).
Signals corresponding to traces of tBuOH are visible.

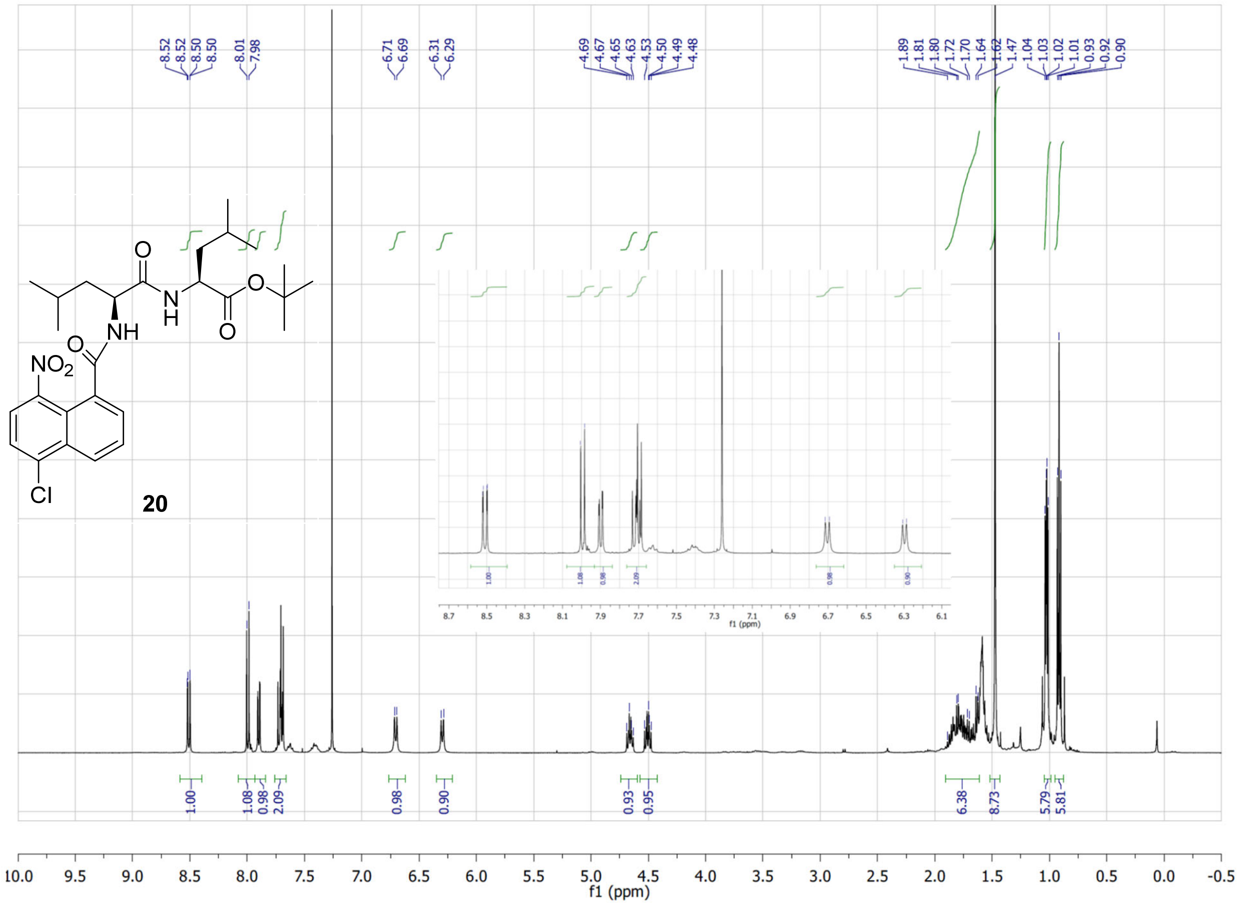

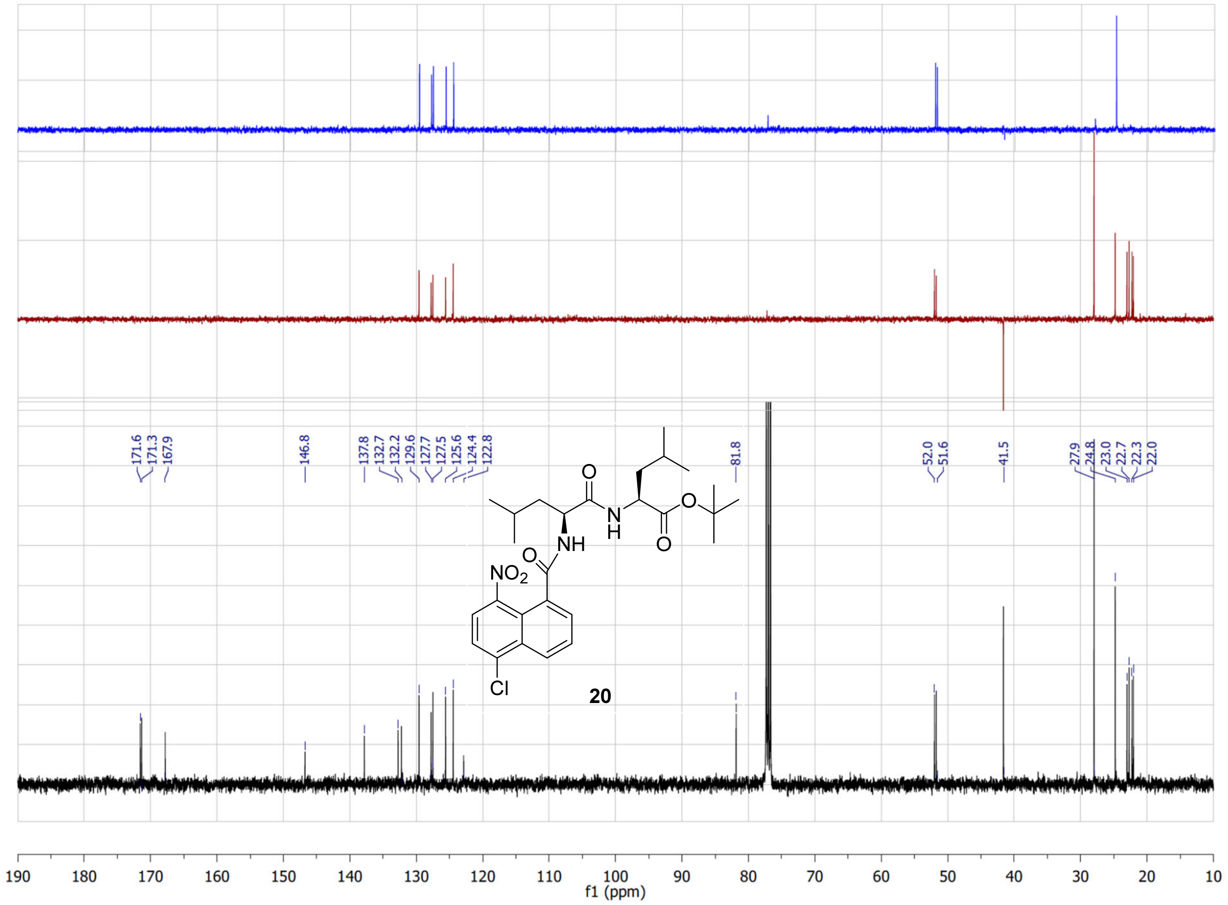

Figure S19. ^{1}H NMR ($CDCl_3$, 400 MHz) *and* ^{13}C ($CDCl_3$, 100 MHz) *spectra of* (**20**).

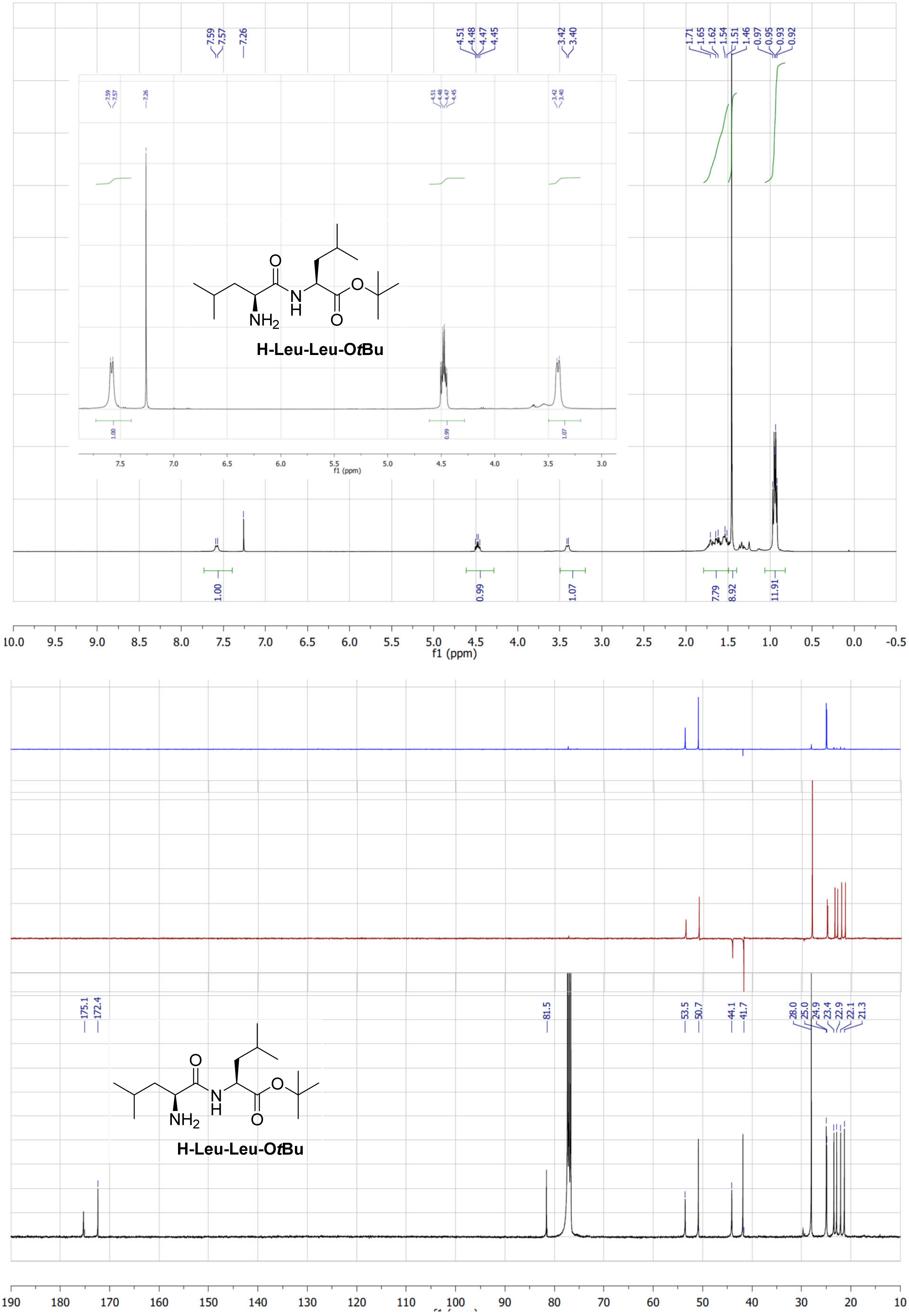

Figure S20. *^{1}H* NMR (*CDCl$_3$*, 400 MHz) *and ^{13}C* (*CDCl$_3$*, 100 MHz) *spectra of* (***H-Leu-Leu-OtBu***).

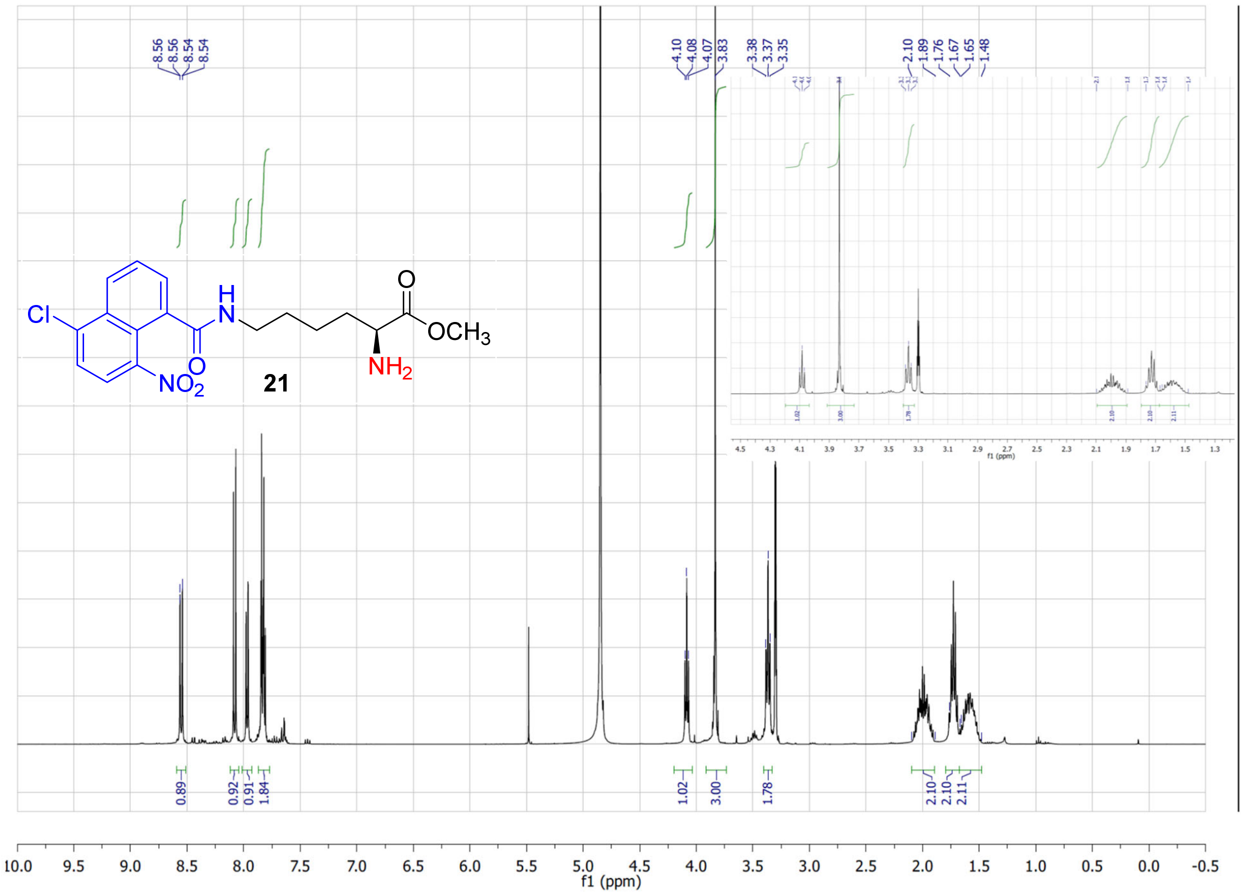

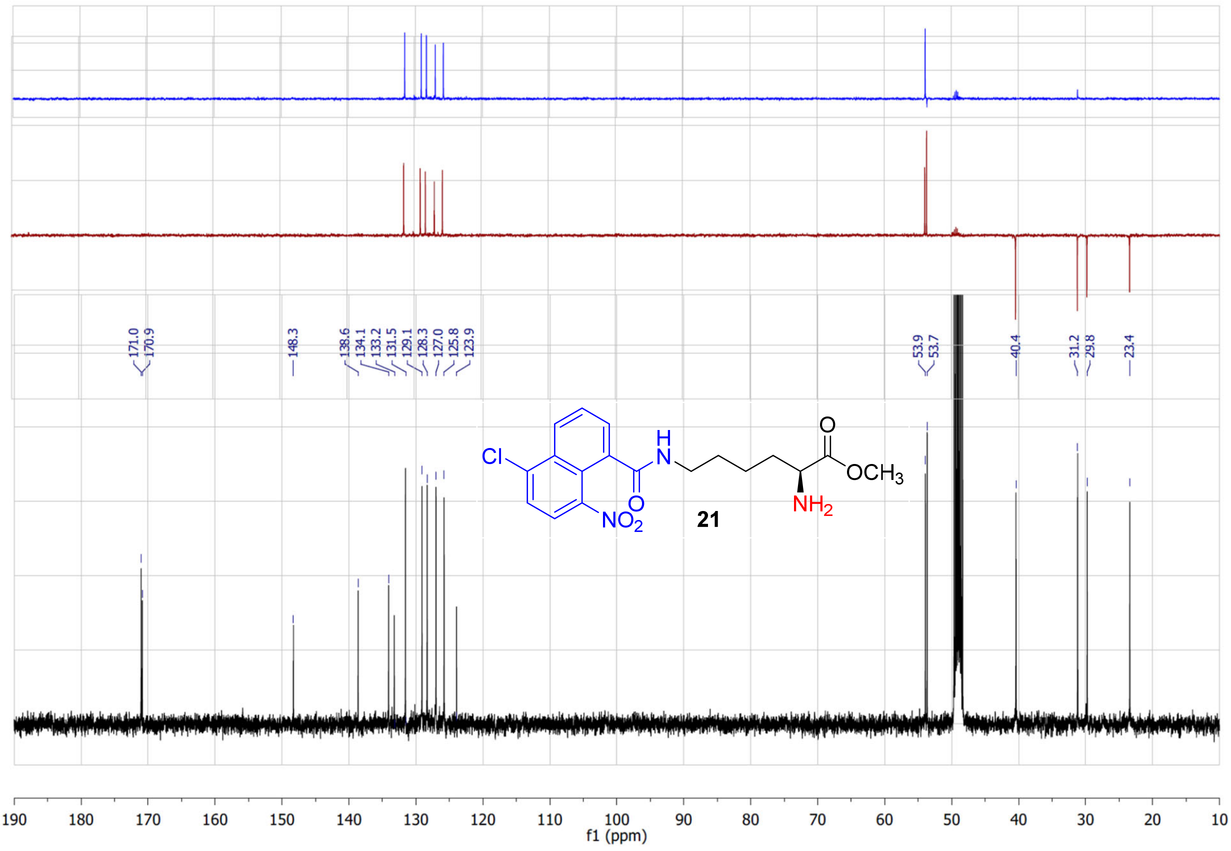

Figure S21. *^{1}H* NMR (*CD_3OD*, 400 MHz) *and* *^{13}C* (*CD_3OD*, 100 MHz) *spectra of* (***21***).

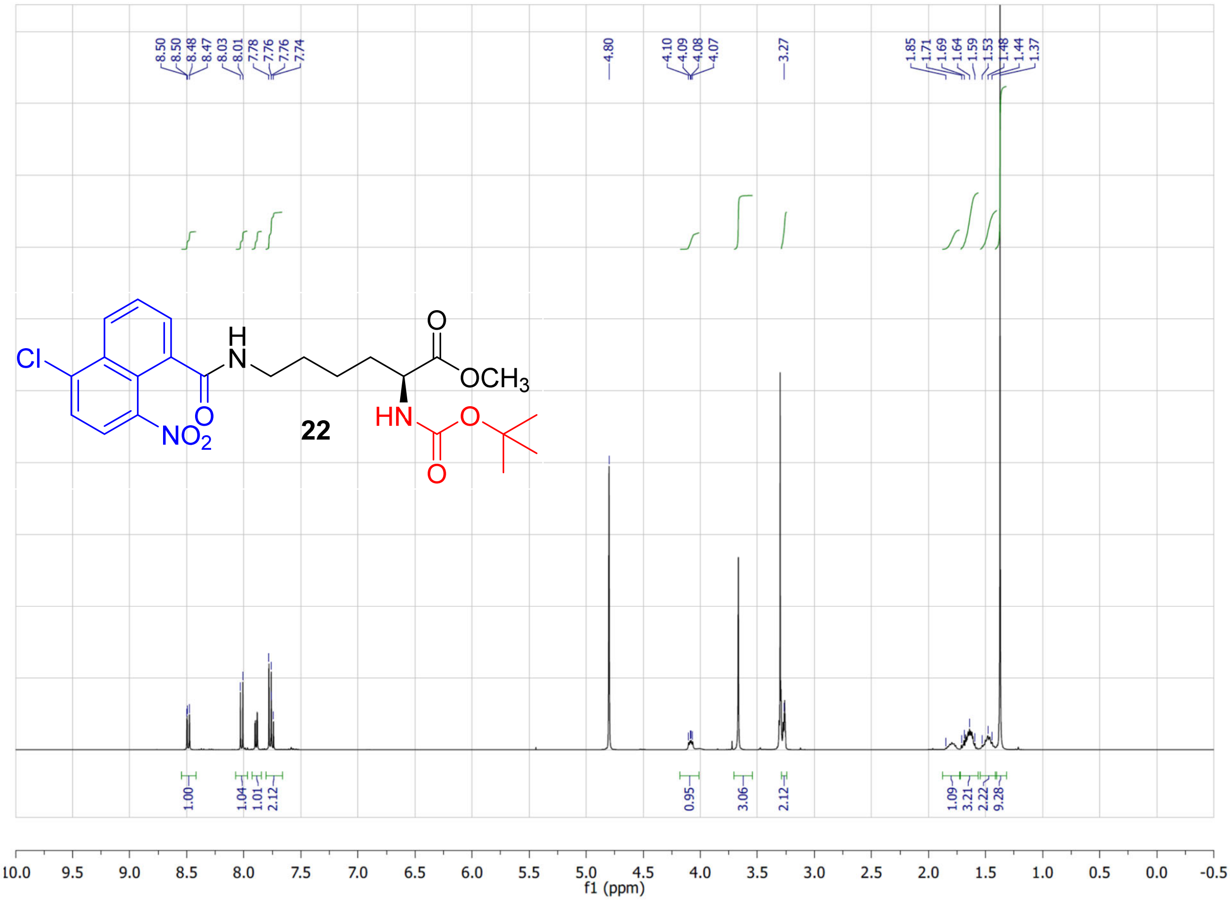

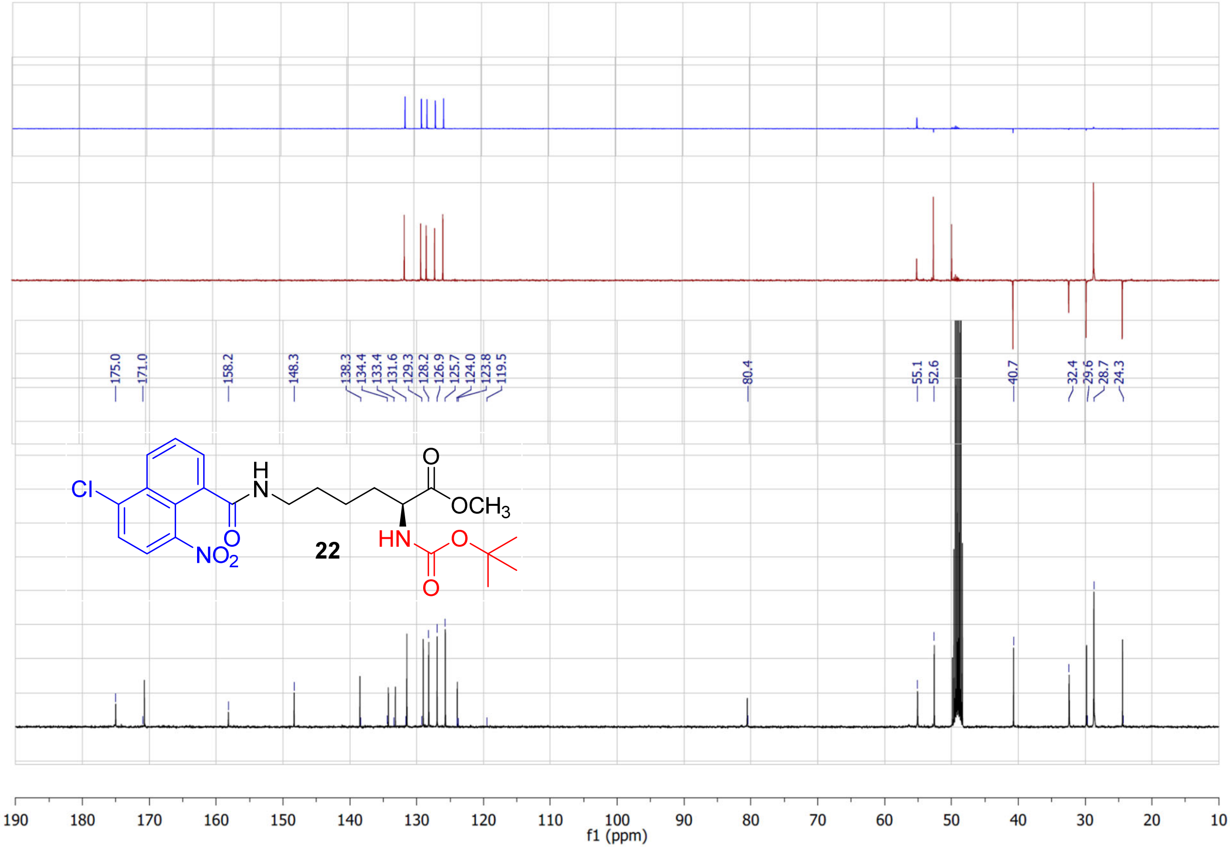

Figure S22. *^{1}H* NMR (*CD_3OD*, 400 MHz) *and ^{13}C* (*CD_3OD*, 100 MHz) *spectra of* (**22**).

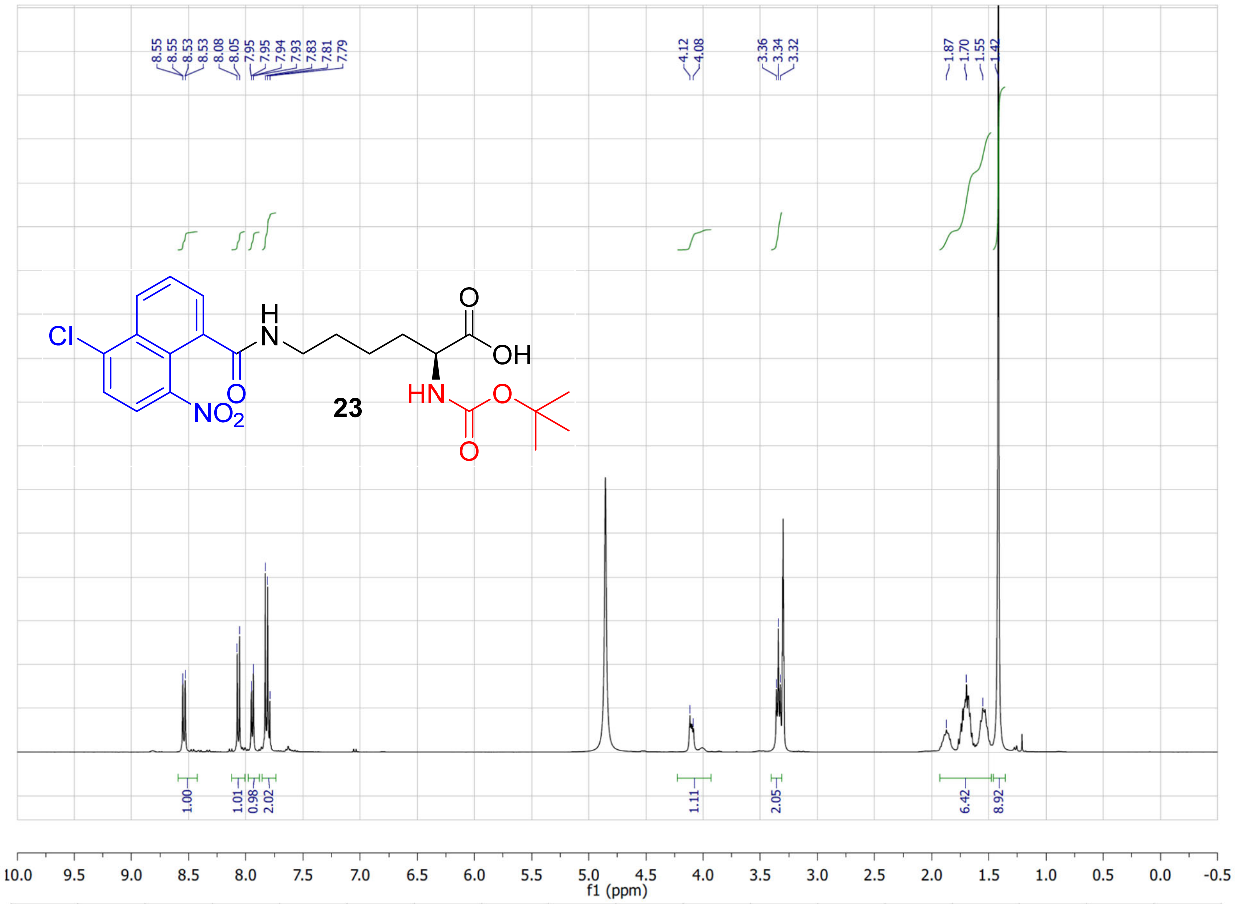

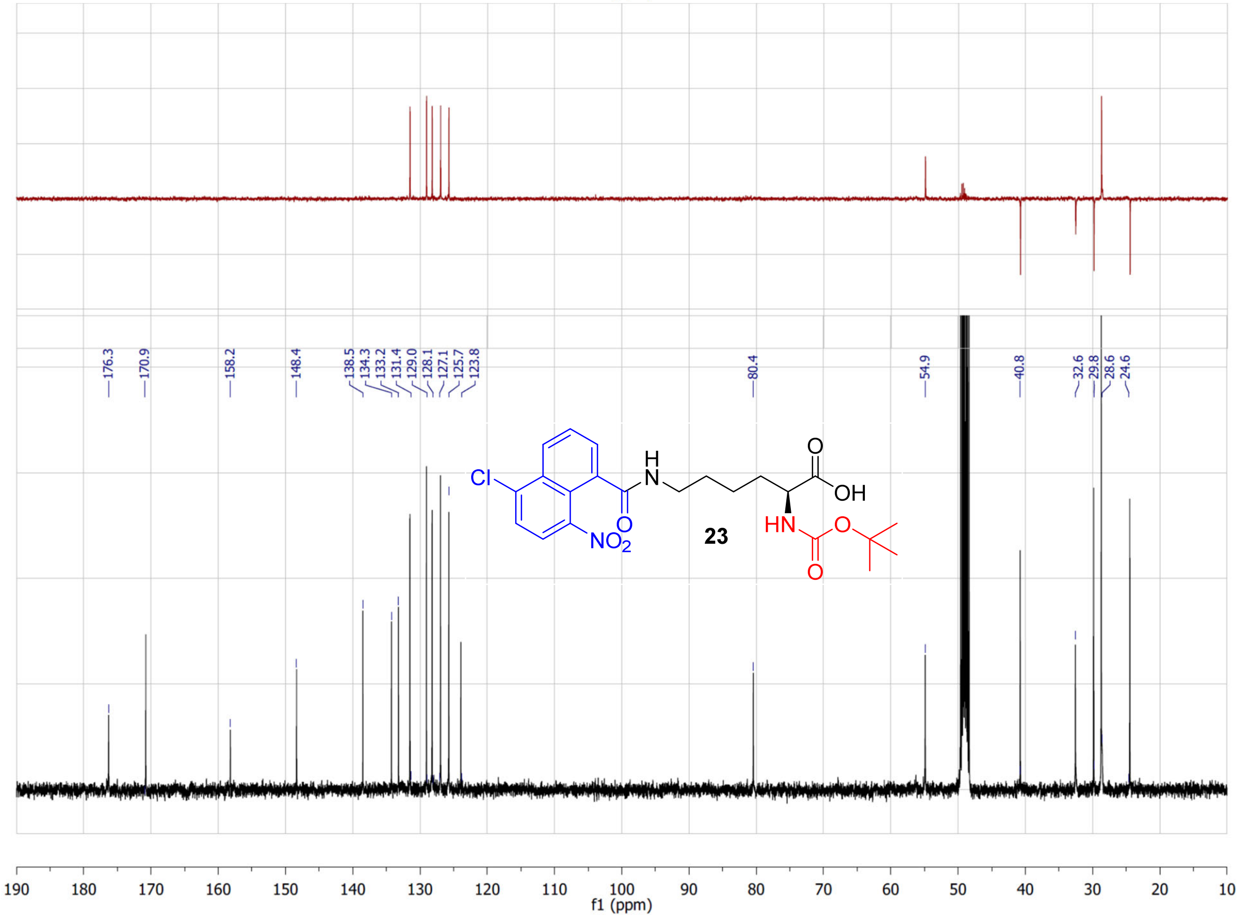

Figure S23. *^{1}H* NMR (*DMSO-d$_6$*, 400 MHz) *and ^{13}C* (*DMSO-d$_6$*, 100 MHz) *spectra of* (***23***).

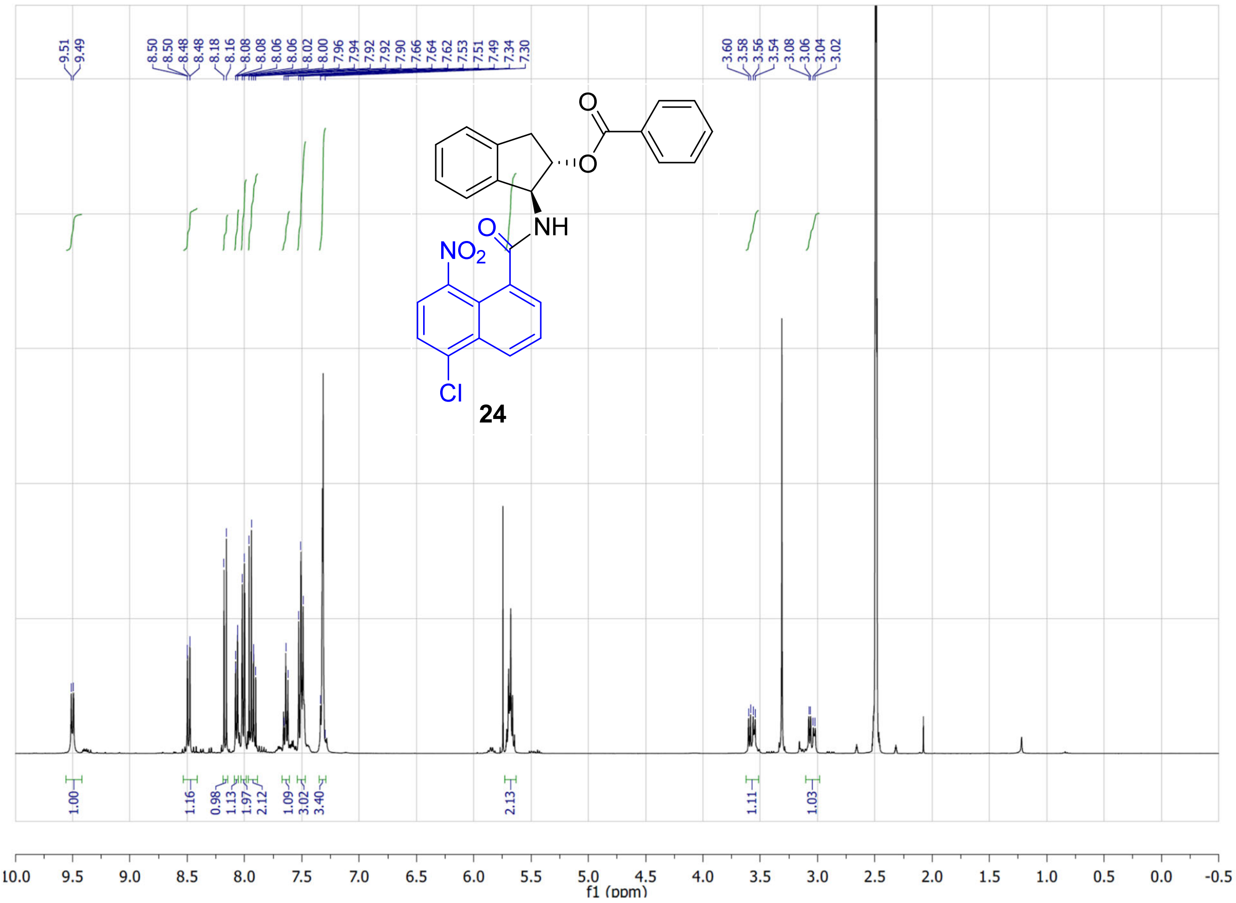

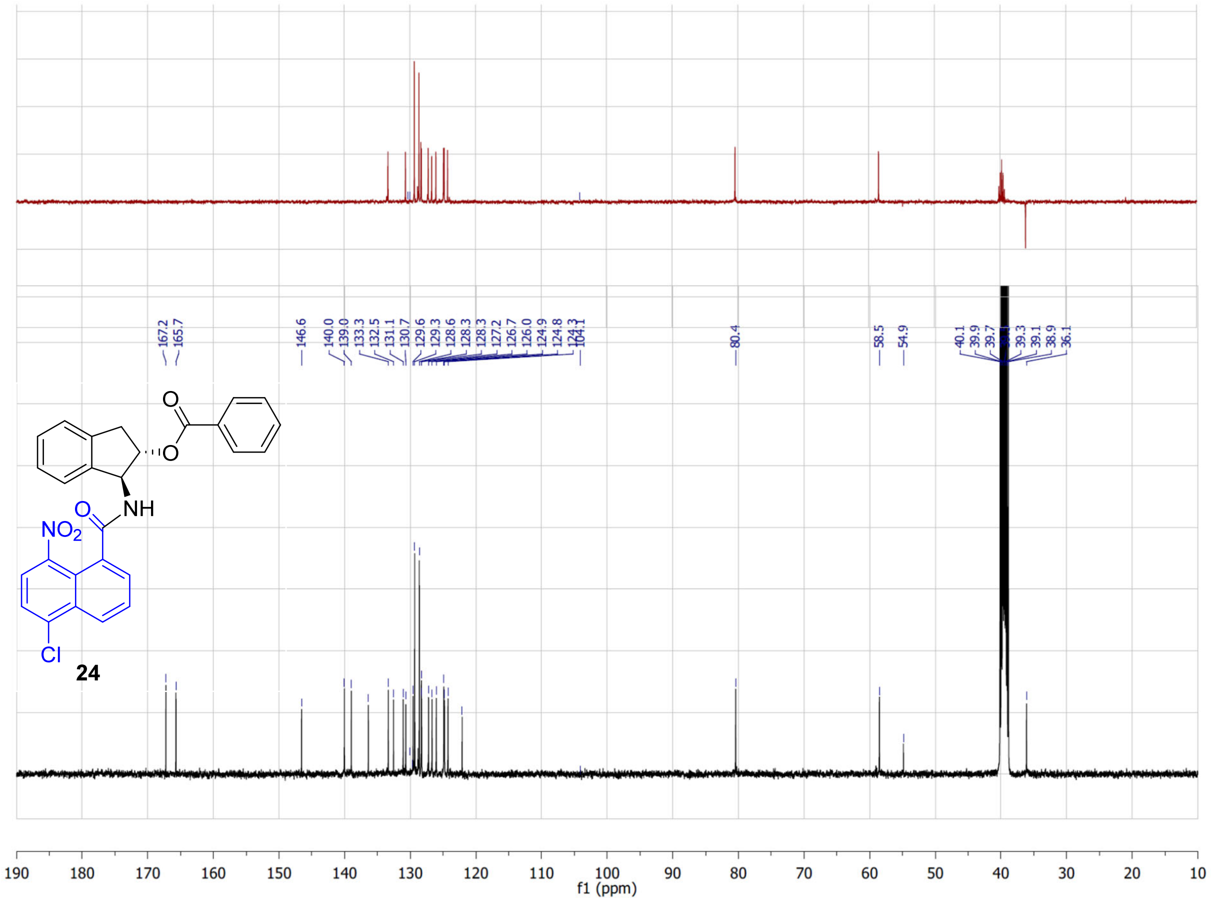

Figure S24. ^{1}H NMR (*DMSO-d$_6$*, 400 MHz) *and* ^{13}C (*DMSO-d$_6$*, 100 MHz) *spectra of* (***24***).

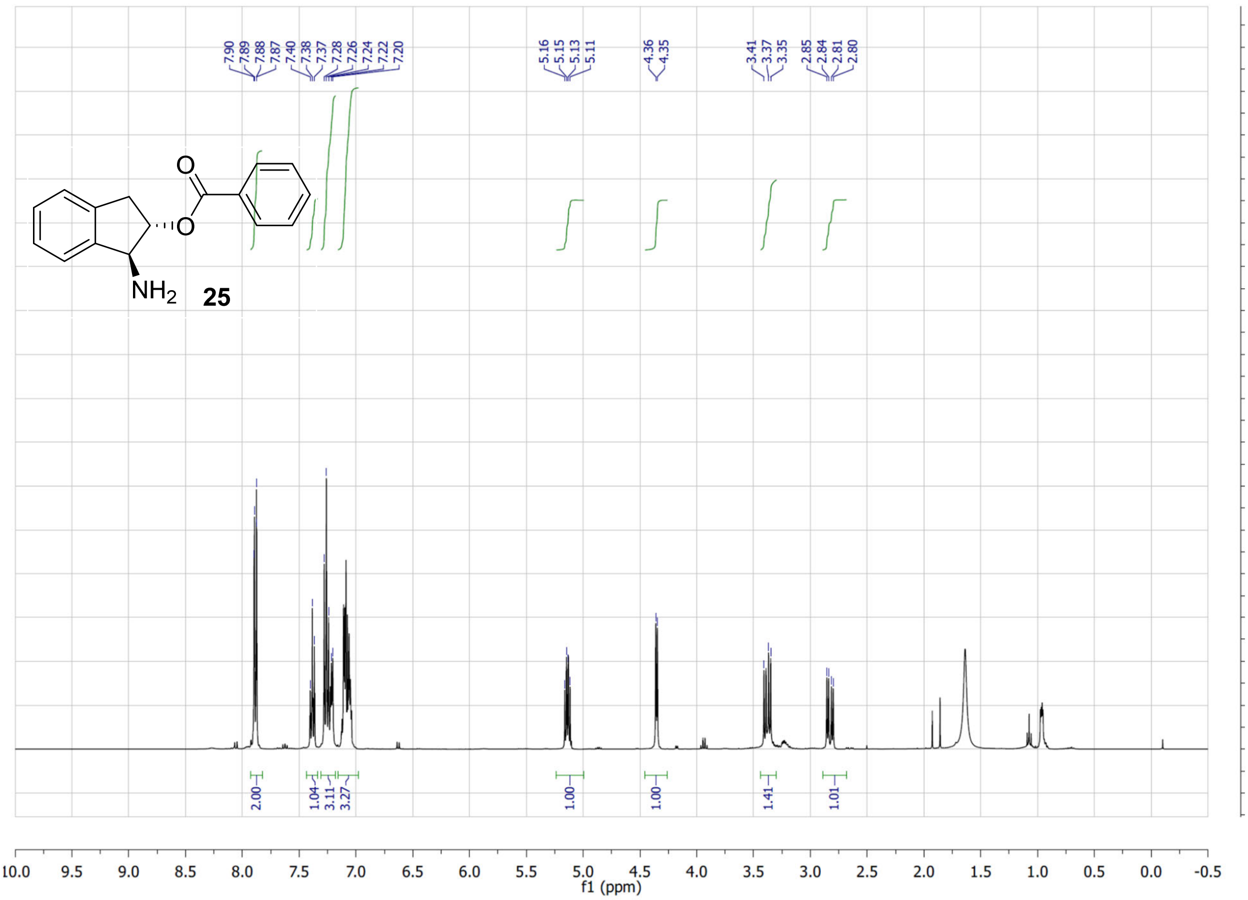

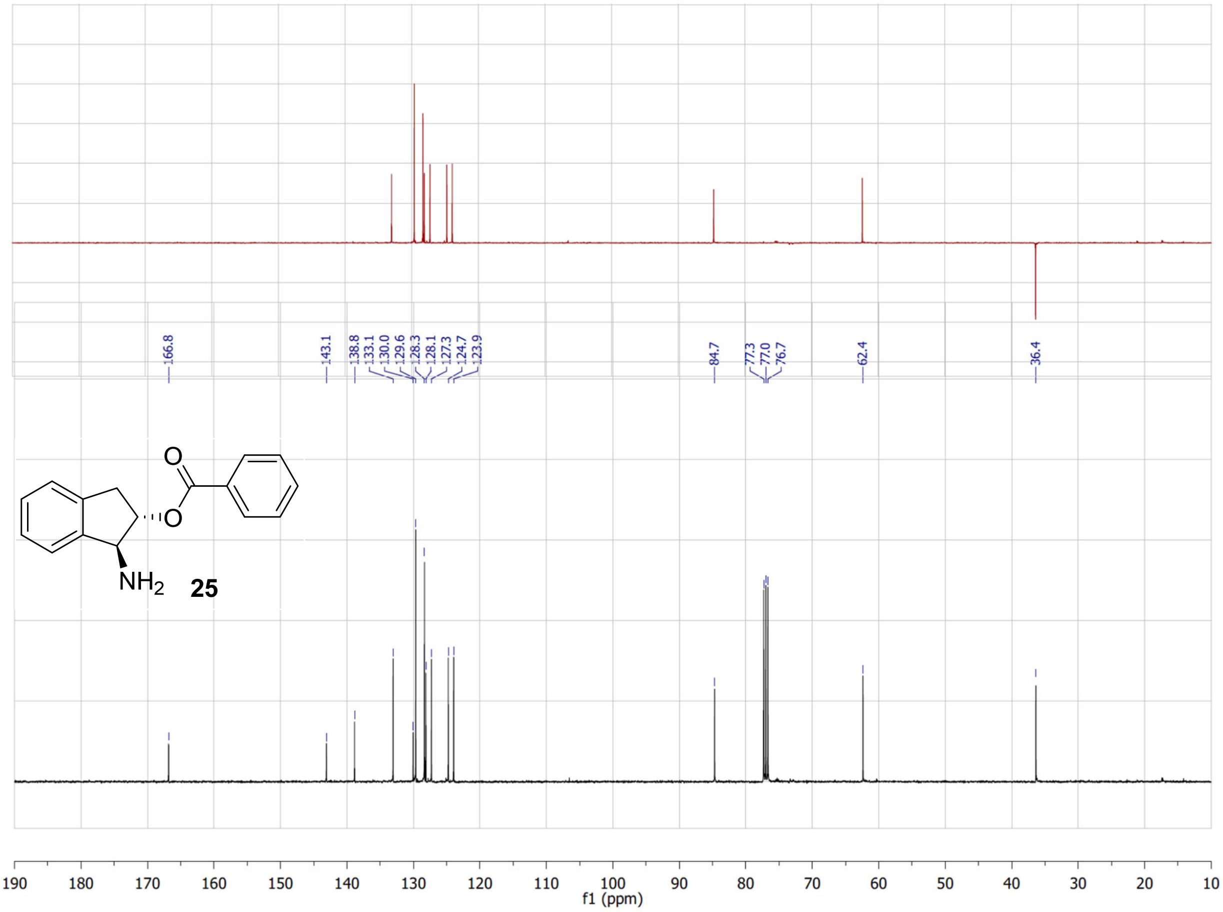

Figure S25. *^{1}H* NMR (*$CDCl_3$*, 400 MHz) *and ^{13}C* (*$CDCl_3$*, 100 MHz) *spectra of* (**25**).

3. Crystallographic data

3.1. *Potassium carboxylate* (K•3)

Table S2. *Crystal data and structure refinement for Potassium carboxylate* (**K•3**) *(CCDC 2252216)*

Empirical formula	$C_{11}H_5BrKNO_5$
Formula weight	350.17
Temperature (K)	298(2)
Wavelength (Å)	1.54178
Crystal system	Monoclinic
Space group	$P2_1/c$
Unit cell dimensions:	
a (Å)	17.5678(7)
b (Å)	6.0655(3)
c (Å)	12.3732(6)
α [º], β[º], γ[º]	90.00,107.532(2), 90.00
Volume	1257.21(10)
Z, Density (calculated) (Mg/m^3)	4, 1.850
Absorption coefficient (mm^{-1})	7.599
F(000)	688
Crystal size (mm)	0.20 x 0.18 x 0.14
2ϴ range for data collection(°)	7.17 to 66.78
Limiting indices	-19≤ h ≤20, -7≤ k ≤6, -14≤ l ≤14
Reflections collected / Independent	7547/2032 (Rint = 0.0602)
Refinement method	Full-matrix least-squares on F^2
Data / restraints / parameters	2032 / 0 / 173
Goodness-of-fit on F^2	2.342
Final R indices [I>2σ(I)]	R1 = 0.1322, wR2 = 0.2947
R indices (all data)	R1 = 0.1363, wR2 = 0.2987
Largest diff. peak and hole ($eÅ^{-3}$)	2.048 and -3.878

$C_{11}H_5BrKNO_5$

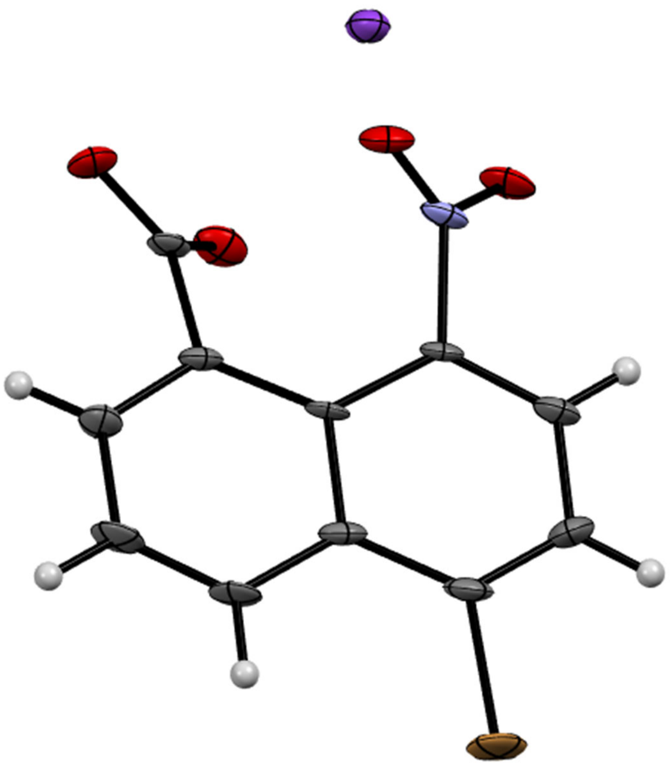

Figure S26. *Molecular structure of compound* **K•3**. *Displacement ellipsoids are drawn at 50 % probability level. Hydrogen atoms are shown as spheres of arbitrary radius.*

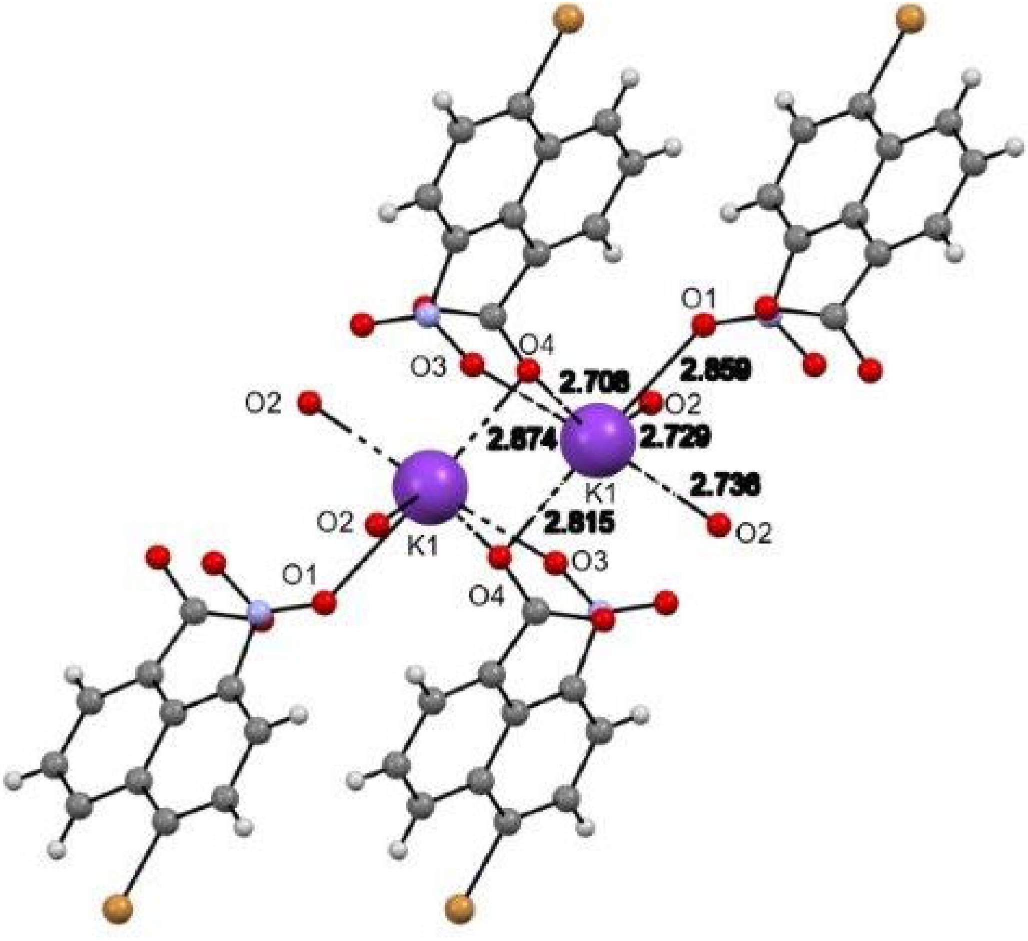

Figure S27. *Views showing the octahedral coordination of the K atom, with the K-O distances (in Å).*

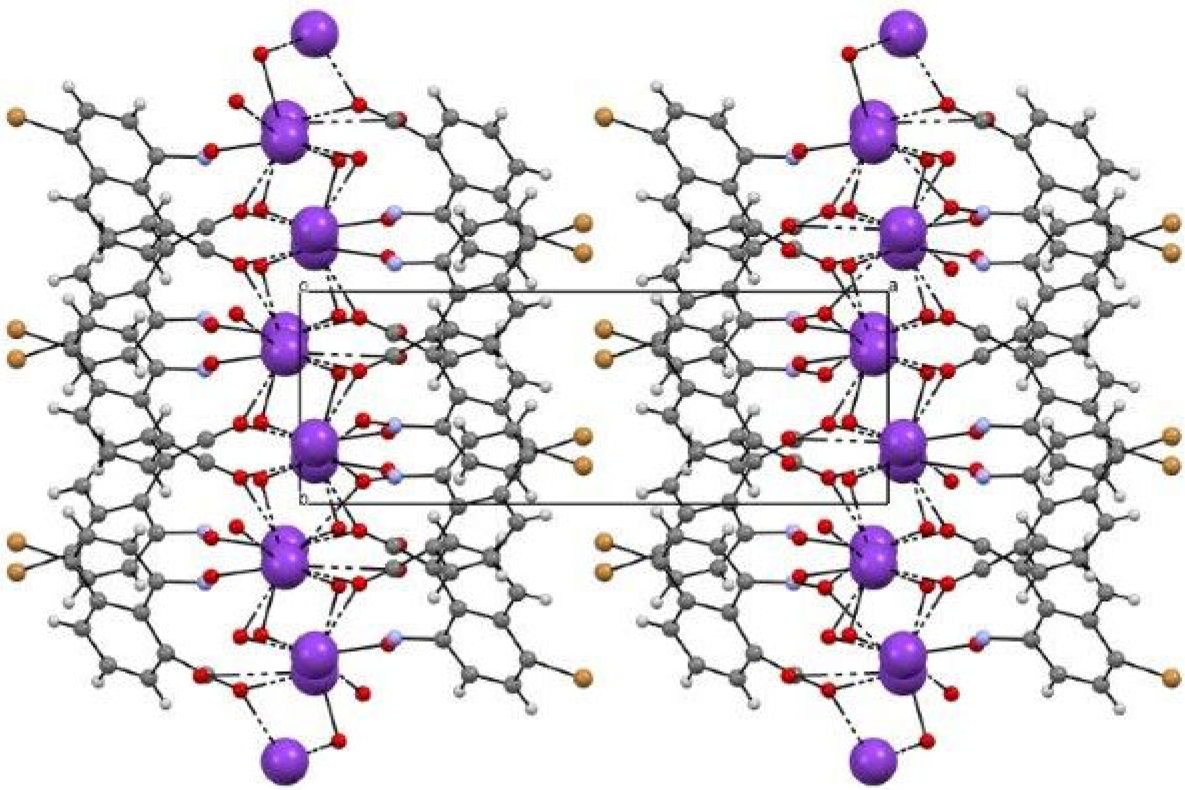

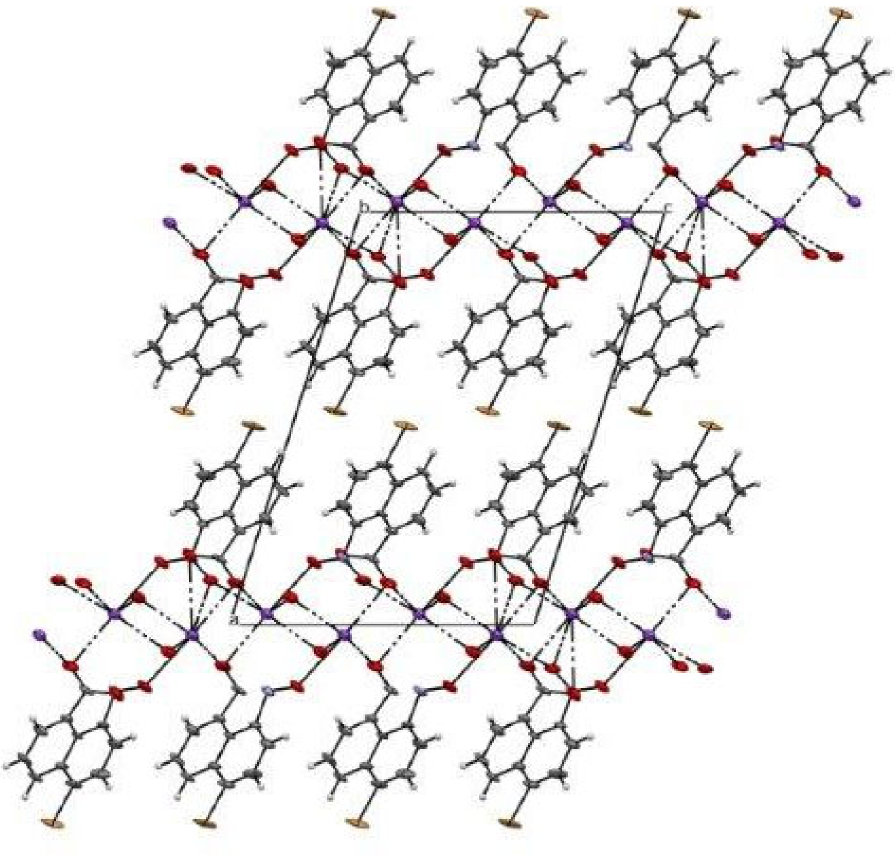

Figure S28. *Crystal packing along b and c axes.*

3.2. Amide 17

Table S3. *Crystal data and structure refinement for amide* ***17*** *(CCDC 2252212)*

Empirical formula	$C_{43}H_{42}Cl_2N_4O_7$
Formula weight	797.71
Temperature (K)	298(2)
Wavelength (Å)	1.54178
Crystal system	Monoclinic
Space group	$P2_1/c$
Unit cell dimensions:	
a (Å)	17.1860(5)
b (Å)	14.3114(5)
c (Å)	35.0105(10)
α [º], β[º], γ[º]	90.00,103.027(2), 90.00
Volume	8389.4(5)
Z, Density (calculated) (Mg/m^3)	8, 1.263
Absorption coefficient (mm^{-1})	1.830
F(000)	3344
Crystal size (mm)	0.16 x 0.12 x 0.10
2Θ range for data collection(°)	2.64 to 67.57
Limiting indices	-20≤ h ≤16, -15≤ k ≤16, -41≤ l ≤41
Reflections collected / Independent	53709/14182 (Rint = 0.0576)
Refinement method	Full-matrix least-squares on F^2
Data / restraints / parameters	14182/ 0 / 1026
Goodness-of-fit on F^2	1.020
Final R indices[I>2σ(I)]	R1 = 0.0660, wR2 = 0.1751
R indices (all data)	R1 = 0.0973, wR2 = 0.1975
Largest diff. peak and hole ($eÅ^{-3}$)	0.435 and -0.327

$C_{43}H_{42}Cl_2N_4O_7$

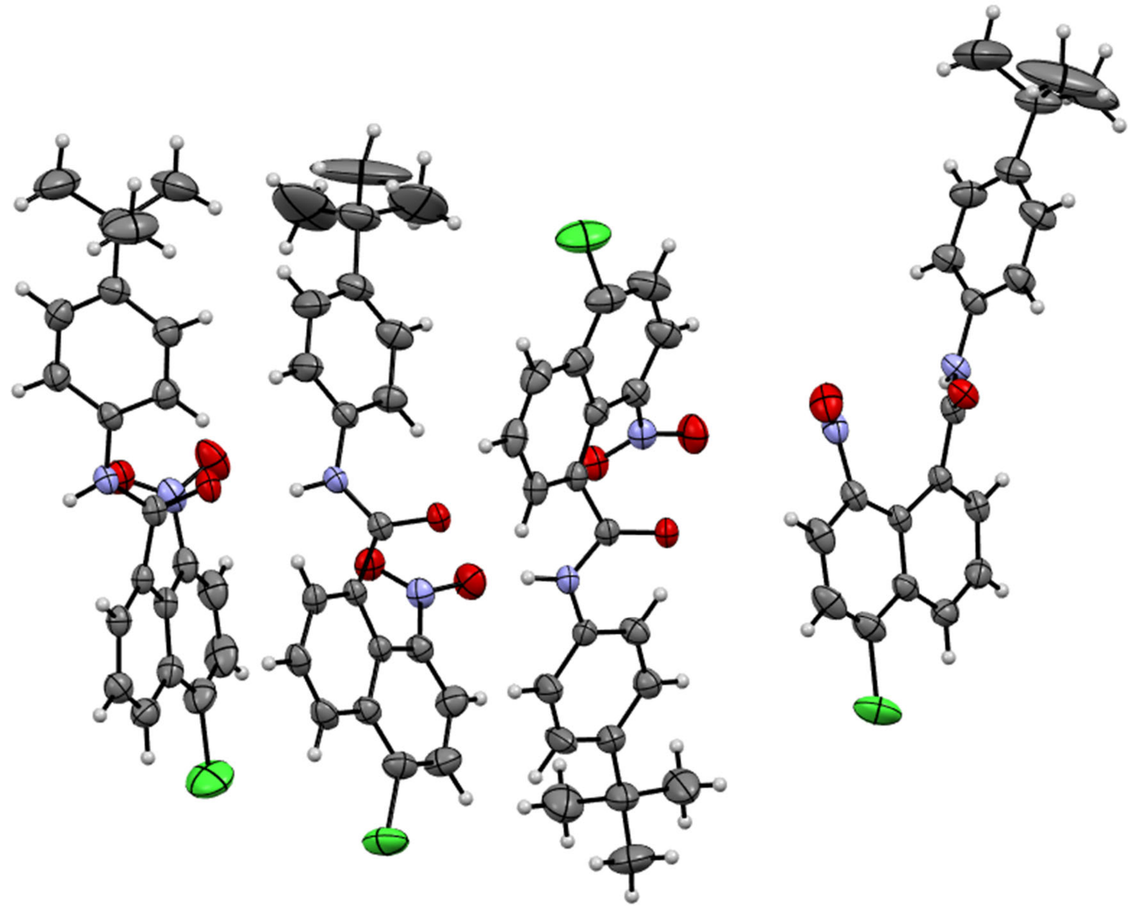

Figure S29. *Molecular structure of amide* ***17****. Displacement ellipsoids are drawn at 50 % probability level. Hydrogen atoms are shown as spheres of arbitrary radius. Solvent molecules have been omitted for clarity.*

4. Modelling studies

Theorical studies were carried out using GAMESS interface for Chem3D 19.1 software [9] using the RHF/3-21G. No imaginary frequencies were observed, and the calculated total energy was - 954413.1802 kcal/mol.
The figure shows a possible geometry for the compound **Ac16**, considering the information from the NMR spectra.

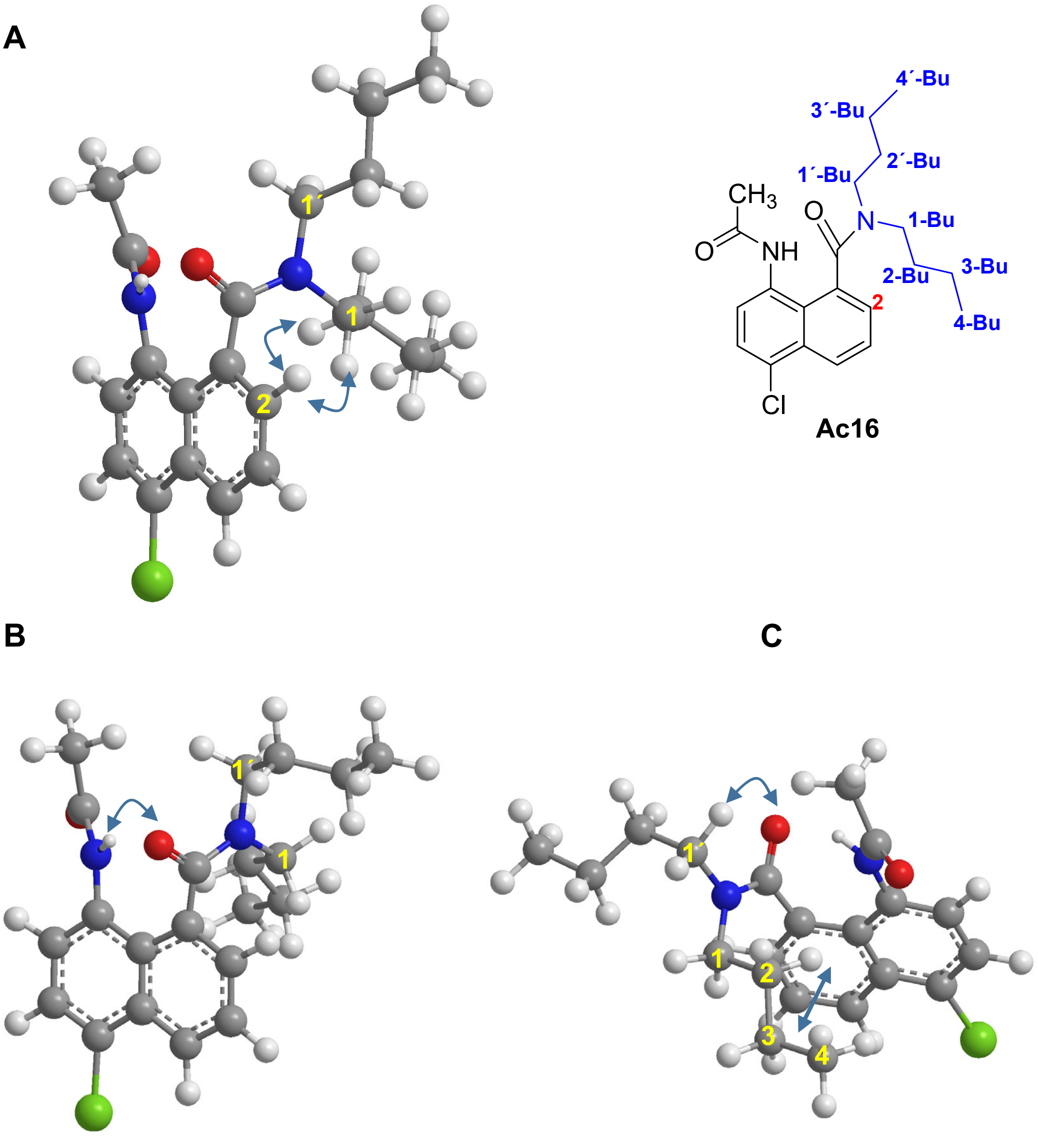

Figure S30. *Methylene protons (1-Bu and 2-Bu) are close to aromatic proton C-2 (**A**). An angular intramolecular H-bond is set between acetamide NH and dibutylamide carbonyl group (**B**). One of the H of the methylene group (1´-Bu) is highly deshielded due to proximity to dibutylamide carbonyl group. Methylene 3-Bu and methyl 4-Bu are over the naphthalene ring.*

Coordinates for the model of **Ac16**

C(1)	-6.028	0.651	1.787
C(2)	-6.263	-0.083	2.877
C(3)	-5.233	-0.489	3.630

C(4)	-3.962	-0.217	3.257
C(5)	-3.704	0.511	2.135
C(6)	-4.771	0.980	1.441
C(7)	-2.940	-0.696	3.994
C(8)	-1.666	-0.521	3.627
C(9)	-1.405	0.134	2.493
C(10)	-2.405	0.633	1.745
N(11)	-4.641	1.816	0.395
C(12)	-5.355	1.944	-0.755
C(13)	-5.026	3.203	-1.534
O(14)	-6.138	1.109	-1.130
C(15)	-1.972	1.199	0.582
O(16)	-1.942	2.401	0.458
N(17)	-1.634	0.349	-0.459
C(18)	-1.378	0.844	-1.820
C(19)	-1.520	-1.111	-0.321
C(20)	-2.611	-1.918	-1.043
C(21)	-2.352	-3.428	-0.873
C(22)	-3.413	-4.293	-1.567
C(23)	0.036	0.549	-2.353
C(24)	0.297	1.250	-3.698
C(25)	1.700	0.932	-4.233
Cl(26)	-5.660	-1.398	5.047
H(27)	-6.904	1.031	1.237
H(28)	-7.309	-0.305	3.154
H(29)	-3.086	-1.283	4.916
H(30)	-0.843	-0.947	4.225
H(31)	-0.350	0.225	2.176
H(32)	-3.824	2.374	0.492
H(33)	-5.071	4.096	-0.867
H(34)	-3.999	3.134	-1.960
H(35)	-5.749	3.361	-2.365
H(36)	-2.130	0.416	-2.520
H(37)	-1.541	1.942	-1.885
H(38)	-0.512	-1.437	-0.665
H(39)	-1.538	-1.440	0.737
H(40)	-3.609	-1.650	-0.619
H(41)	-2.627	-1.687	-2.133
H(42)	-1.349	-3.680	-1.298
H(43)	-2.327	-3.691	0.212
H(44)	-3.183	-5.377	-1.439
H(45)	-4.426	-4.107	-1.142
H(46)	-3.450	-4.082	-2.661
H(47)	0.792	0.884	-1.604
H(48)	0.178	-0.545	-2.513
H(49)	-0.464	0.928	-4.449
H(50)	0.194	2.354	-3.571
H(51)	1.885	1.441	-5.207
H(52)	2.482	1.272	-3.516
H(53)	1.831	-0.163	-4.394

5. References

[1] Armarego, W. L. F.; Perrin, D. D. Purification of Laboratory Chemicals; 3th ed.; Pergamon Press: Oxford, 1988.
[2] Gottlieb, H. E.; Kotlyar, V.; Nudelman, A. *J. Org. Chem.* **1997**, *62*, 7512-7515.
[3] SAINT-NT Version 6.0, Madison, Wisconsin, USA: Bruker–AXS, 2001.
[4] SADABS 2008/1, Krause, L.; Herbst-Irmer, R.; Sheldrick, G. M.; Stalke. D. *J. Appl. Crystallogr*. **2015**, *48*, 3-10.
[5] SHELXL 2014/4, Sheldrick, G. M. *Acta Cryst. A* **2015**, *71*, 3-8.
[6] SHELXL 2014/7, Sheldrick, G. M. *Acta Cryst. A,* **2015**, *71*, 3-8.
[7] Shmueli, U. ed. International Tables for Crystallography; Springer: New York, 2006.
[8] Macrae, C. F.; Edgington, P. R.; McCabe, P.; Pidcock, E.; Shields, G. P. Taylor, R.; Towler, M.; Van de Streek, J. J. *J. Appl. Cryst.* **2006**, *39*, 453-457.
[9] Barca, G. M. J.; Bertoni, C.; Carrington, L.; Datta, D.; De Silva, N.; Deustua, J. E.; Fedorov, D. G.; Gour, J. R.; Gunina, A. O.; Guidez, E.; Harville, T.; Irle, S.; Ivanic, J.; Kowalski, K.; Leang, S. S.; Li, H.; Li, W.; Lutz, J. J.; Magoulas, I.; Mato, J.; Mironov, V.; Nakata, H.; Pham, B. Q.; Piecuch, P.; Poole, D.; Pruitt, S. R.; Rendell, A. P.; Roskop, L. B.; Ruedenberg, K.; Sattasathuchana, T.; Schmidt, M. W.; Shen, J.; Slipchenko, L.; Sosonkina, M.; Sundriyal, V.; Tiwari, A.; Galvez Vallejo, J. L.; Westheimer, B.; Włoch, M.; Xu, P.; Zahariev, F.; Gordon, M. S. *J. Chem. Phys.* **2020**, *152*, 154102.
[10] Repine, J. T.; Johnson, D. S.; White, A. D.; Favor, D. A.; Stier, M. A.; Yip, J.; Rankin, T.; Ding, Q.; Maiti, S. N. *Tetrahedron Lett.* **2007**, *48*, 5539–5541.
[11] van der Linden, W. A.; Willems, L. I.; Shabaneh, T. B.; Li, N.; Ruben, M.; Florea, B. I.; van der Marel, G. A.; Kaiser, M.; Kisselev, A. F.; Overkleeft, H. S. *Org. Biomol. Chem.* **2012**, *10*, 181-194.
